# Interaction, immune infiltration characteristics and prognostic modeling of efferocytosis-related subtypes in glioblastoma

**DOI:** 10.1186/s12920-023-01688-4

**Published:** 2023-10-18

**Authors:** Songyun Zhao, Qi Wang, Yuankun Liu, Pengpeng Zhang, Wei Ji, Jiaheng Xie, Chao Cheng

**Affiliations:** 1 Department of Neurosurgery, Affiliated Wuxi People’s Hospital of Nanjing Medical University, Wuxi, China; 2 Department of Gastroenterology, Affiliated Hospital of Jiangsu University, Zhenjiang, China; 3 Department of Lung Cancer Surgery, Tianjin Medical University Cancer Institute and Hospital, Tianjin, China; 4 The First Affiliated Hospital of Nanjing Medical University, Nanjing, China; 5 Department of Plastic Surgery, Xiangya Hospital, Central South University, Changsha, China

**Keywords:** GBM, Efferocytosis, PCA, TME, PCR, Immunotherapy, Risk signature

## Abstract

**Background:**

Efferocytosis is a biological process in which phagocytes remove apoptotic cells and vesicles from tissues. This process is initiated by the release of inflammatory mediators from apoptotic cells and plays a crucial role in resolving inflammation. The signals associated with efferocytosis have been found to regulate the inflammatory response and the tumor microenvironment (TME), which promotes the immune escape of tumor cells. However, the role of efferocytosis in glioblastoma multiforme (GBM) is not well understood and requires further investigation.

**Methods:**

In this study, we conducted a comprehensive analysis of 22 efferocytosis-related genes (ERGs) by searching for studies related to efferocytosis. Using bulk RNA-Seq and single-cell sequencing data, we analyzed the expression and mutational characteristics of these ERGs. By using an unsupervised clustering algorithm, we obtained ERG clusters from 549 GBM patients and evaluated the immune infiltration characteristics of each cluster. We then identified differential genes (DEGs) in the two ERG clusters and classified GBM patients into different gene clusters using univariate cox analysis and unsupervised clustering algorithms. Finally, we utilized the Boruta algorithm to screen for prognostic genes and reduce dimensionality, and the PCA algorithm was applied to create a novel efferocytosis-related scoring system.

**Results:**

Differential expression of ERGs in glioma cell lines and normal cells was analyzed by rt-PCR. Cell function experiments, on the other hand, validated TIMD4 as a tumor risk factor in GBM. We found that different ERG clusters and gene clusters have distinct prognostic and immune infiltration profiles. The ERG signature we developed provides insight into the tumor microenvironment of GBM. Patients with lower ERG scores have a better survival rate and a higher likelihood of benefiting from immunotherapy.

**Conclusions:**

Our novel efferocytosis-related signature has the potential to be used in clinical practice for risk stratification of GBM patients and for selecting individuals who are likely to respond to immunotherapy. This can help clinicians design appropriate targeted therapies before initiating clinical treatment.

**Supplementary Information:**

The online version contains supplementary material available at 10.1186/s12920-023-01688-4.

## Introduction

Glioblastoma (GBM) is the most common primary brain malignancy, accounting for approximately 12%-15% of all brain tumors [1, 2]. Despite significant advancements in chemotherapy, radiation therapy, and surgical treatment, GBM patients’ 5-year survival rate remains less than 5% [3]. Although epidemiological studies suggest that ionizing radiation increases glioblastoma incidence [4], most GBM patients have no clear pathogenetic cause. The widespread heterogeneity within and between individuals is the root cause of GBM treatment failure, making it one of the most aggressive and treatment-resistant malignancies [5]. Therefore, discovering new biomarkers and establishing effective molecular staging systems to select appropriate treatments for GBM patients is crucial, as molecular alterations are increasingly important in glioma classification and grading [6, 7].

Phagocytes, such as macrophages and immature dendritic cells, play a vital role in efferocytosis, the process of recognizing and engulfing dying cells during apoptosis [8]. Unlike regular cytokinesis, efferocytosis preserves the membrane integrity of dead cells, preventing exposure to immunogenic substances and avoiding secondary cell damage caused by inflammatory responses [9, 10]. Although numerous genes that promote efferocytosis are involved in tumor development and metastasis and are frequently overexpressed in various cancers, including lung cancer, breast cancer, and leukemia [11, 12]. Current theories suggest that efferocytosis may contribute to tumor progression due to the unaccompanied release of inflammatory factors and production of killer effector T cells, resulting in a suppressive immune microenvironment and the immune escape of tumor cells [12, 13]. In contrast, uncleared apoptotic cells and secondary necrosis promote a proinflammatory environment and antitumor immunity [14]. The receptors for phagocytes, such as tumor-associated macrophages (TAM), have been extensively studied. For example, MerTK is involved in epidermal growth factor receptor (EGFR) inhibitor resistance in non-small-cell lung cancer [15], and blocking phagocytic receptors with the membrane-linked protein V can effectively slow tumor progression in prostate cancer [16]. Additionally, TIM-4 acts as a PS receptor on the surface of phagocytes and promotes angiogenesis in colorectal cancer by upregulating vascular endothelial growth factor (VEGF) [17].

The crucial role of efferocytosis in cancer development and progression is attributed to its effect on tumor cell growth, metastasis, EMT, and angiogenesis [18]. Although traditional oncology therapies such as chemotherapy and radiotherapy trigger apoptosis and efferocytosis, they also lead to tumor inflammation and limit antitumor immunity [19]. Studies have indicated that solely blocking efferocytosis cannot completely inhibit the production of tumor immunosuppressive cells and mediators [20]. However, combined inhibition of tumor cell apoptosis and efferocytosis has been observed to effectively suppress metastatic recurrence of tumors. Notably, the immunosuppressive microenvironment in GBM patients forms a comprehensive and self-sufficient system [21]. Additionally, the efferocytosis process may function as an immune checkpoint similar to PD-1/PD-L1, which could be targeted for therapeutic interventions [22]. Therefore, developing a combination therapy that targets both conventional oncology therapy and efferocytosis presents a significant technical challenge.

In this study, we categorized 20 genes associated with efferocytosis into different clusters and assessed their impact on the prognosis of GBM patients. To achieve this, we used an unsupervised consensus clustering method and combined three GBM cohorts. The univariate Cox analysis and Boruta algorithm were used to identify the differentially expressed genes among the ERG clusters. Then, a scoring system was established based on the PCA algorithm. The primary objective of the study was to determine whether this novel efferocytosis characteristic could accurately predict the prognosis of GBM patients and assist medical professionals in identifying potentially responsive patients for the development of effective immunotherapies.

## Materials and methods

### Acquisition of raw data

**Fig. 1:**
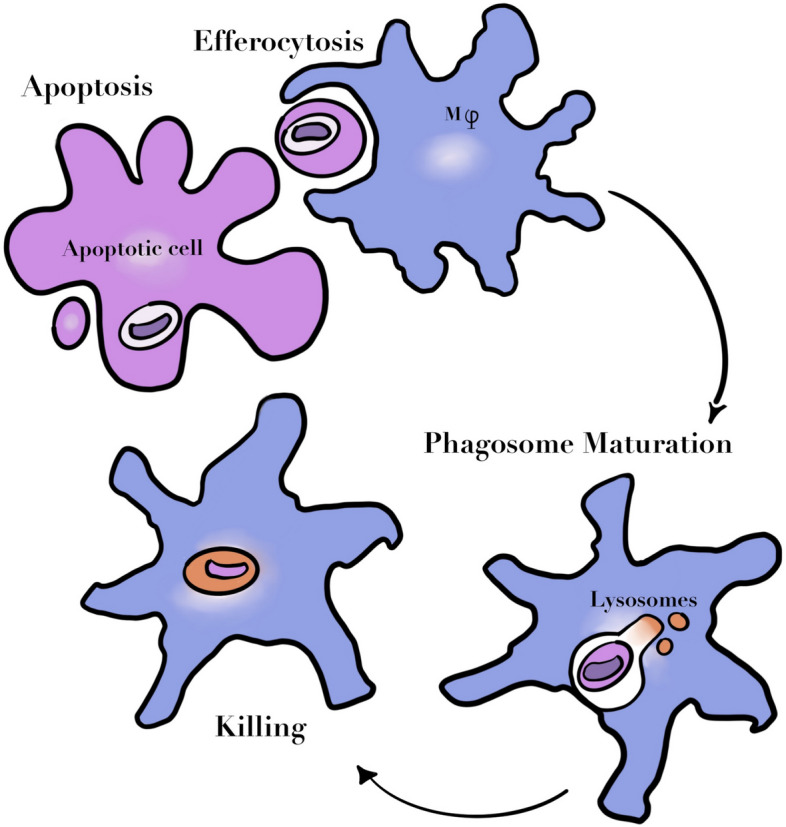
Brief process of efferocytosis

We collected RNA-seq data and clinical information of GBM patients from two databases, UCSC Xena (https://xena.ucsc.edu/) and CGGA (http://www.cgga.org.cn/). In addition, we downloaded mutation data of TCGA-GBM patients and removed 8 duplicates, leaving us with a TCGA cohort comprising 161 GBM tissue samples and 5 normal samples. The CGGA-325 cohort contained 139 GBM samples, while the CGGA693 cohort had 249 GBM samples. Gene expression profiles were measured using the transcript per million estimation and log2-based transformation. After combining the mRNA expression data of GBM patients from these three cohorts, we used the “sva” package [23] to perform batch correction. We identified 103 efferocytosis-related genes from the genecards portal (http://www.genecards.org/) using the keyword “efferocytosis”. After comparing these genes on the Pubmed website (https://pubmed.ncbi.nlm.nih.gov/) for their research applications in efferocytosis, we selected the most plausible 22 genes. These 22 efferocytosis-related genes are listed in Supplementary Table 1, and we have provided a brief illustration of the efferocytosis process in Fig. 1.

### Consensus unsupervised clustering

To identify distinct ERG clusters based on the expression of 20 ERGs, we employed consensus unsupervised cluster analysis using the “ConsensusClusterPlus” software program [24]. We used the “Pam” algorithm with “Euclid” as the distance measure, and resampled items with a rate of 80% for 1000 replications to determine the optimal k value based on the proportion of ambiguous clustering (PAC). Next, we used the “limma” package to identify differentially expressed genes among the various ERG clusters, where expression levels were considered significant if |log2 FC| exceeded 1 and the adjusted *P*-value was below 0.05 [25, 26].

### Functional enrichment analysis

To explore the biological functions of the differentially expressed genes in various ERG clusters, we conducted GO enrichment and KEGG pathway analyses. We used the clusterProfiler package and applied the BH method to adjust the *P*-value [27]. It is important to note that the GO enrichment functions include cellular components, biological processes, and molecular functions [28, 29].

### Establishment of the efferocytosis-related signature

After conducting one univariate Cox analysis (with a significance level of *P* < 0.05), we proceeded to a second clustering analysis on differentially expressed genes (DEGs) identified in the various ERG clusters. Among the DEGs associated with the ERG gene cluster, we defined genes positively associated with the ERG gene cluster as gene signature A and genes negatively associated with the ERG gene cluster as gene signature B. We utilized the “Boruta” software package to further screen significant genes among the candidate genes, applying the Boruta algorithm with a maxRuns value of 500. By using the Boruta algorithm, we retained the genes identified as “confirmed”. Principal component analysis (PCA) was applied to reduce the dimensionality of the ERG gene cluster. Subsequently, an ERG score was assigned to each patient by computing the score for each GBM sample using the following formula: score = ∑PCA A—∑PCA B. To classify GBM patients into high-risk (HR) and low-risk (LR) groups, we used the “surv_cutpoint” function from the “survminer” package to determine the optimal cutoff values [30].

### Prediction of immunotherapy

We aimed to evaluate the ability of the ERG signature to predict the response to immunotherapy in GBM patients, by utilizing the Tumor Immune Dysfunction and Exclusion (TIDE) algorithm that integrates both tumor immune dysfunction and exclusion factors [31]. We obtained a subset of genes associated with cancer and immunity by using the website developed by Xu et al. [32] (http://biocc.hrbmu.edu.cn/tip/) and selecting genes that were positively associated with anti-PD-L1 drug response based on Mariathasan’s study features [33]. We then applied the GSVA method to calculate the enrichment scores for gene signatures associated with the cancer immune cycle, considering *p*-values less than 0.05 to indicate statistically significant differences between the two groups. To assess the relationship between risk scores and the two genetic features mentioned above, we used the R package “ggcor”.

### Immune microenvironment-related analysis

We utilized various algorithms for immune cell infiltration analysis, including XCELL [34], TIMER [35], QUANTISEQ, MCPCOUNT, EPIC [36], CIBERSORT [37] and CIBERSORT- ABS. The results from these algorithms were compared and further analyzed using the “ComplexHeatmap” R package [38, 39]. We examined the correlation between immune cells and risk scores using Spearman correlation analysis. To differentiate GBM patients with low ERG scores from those with high scores based on their immune cell features, we employed the single-sample gene set enrichment analysis (ssGSEA) technique. We also estimated the immune and stromal scores of each glioma sample using the R program “ESTIMATE,” which provides an estimate of the quantities of immune and stromal components present in vivo [40]. To comprehensively investigate the tumor microenvironment’s heterogeneity in different datasets and cell types, we utilized the Tumor Immune Single-Cell Hub (TISCH) database. TISCH is an extensive single-cell RNA-seq database dedicated to the tumor microenvironment (http://tisch.comp-genomics.org).

### Drug sensitivity

The semi-inhibitory concentration, also known as IC50, represents the drug concentration that corresponds to a 50% ratio of apoptotic cells to the total number of cells and is often used to assess a drug’s ability to induce apoptosis. A lower IC50 value indicates a higher induction of apoptosis, whereas a higher value suggests that the cells are more tolerant to the medication. To assess the efficacy of our ERG signature in targeted chemotherapy, we used the “pRRophetic” tool to compute the IC50 values for various chemotherapeutic agents typically used in GBM treatment [41]. We compared the IC50 values between the high and low-scoring groups and evaluated the patients’ sensitivity to each drug.

### Transfection of cells and real-time PCR

U251MG, LN229, and SW1783 human glioma cells and human astrocytes (NHA) were cultured in Dulbecco’s Modified Eagle’s Medium (DMEM, Gibco, C11995500BT, Canada) supplemented with 10% fetal bovine serum (FBS, Gibco, 10091148, Canada) and 1 × penicillin/streptomycin (Gibco, 15,140–122, Canada). All cultures were maintained in a CO2 incubator (TFS3111, USA) at 37 °C with 5% CO2. TIMD4 gene knockdown was achieved using small interfering RNA (siRNA). The specific TIMD4 siRNA sequences can be found in Supplementary Table 2. In brief, cells were seeded at 50% confluency in 6-well plates and transfected with negative control (NC) and siBARD1 using Lipofectamine 3000 (Invitrogen, USA).

Total RNA was extracted from cell lines and tissues using TRIzol (Sigma-Aldrich, T9424, America) according to the manufacturer’s instructions. cDNA was synthesized using the PrimeScriptTM RT Reagent Kit (Takara, RR047, Japan). Real-time polymerase chain reaction (RT-PCR) was performed using SYBR Green Master Mix (Q111-02, Vazyme) to quantify mRNA expression levels normalized to GAPDH mRNA levels. The 2 − ΔΔCt method was used to calculate the expression levels. All primers were provided by Qingdao BioScience (Beijing, China), and the primer sequences can be found in Supplementary Table 2.

### Cell counting Kit8 assay and transwell assay

First, cells (1000 cells per well) were seeded into a 96-well plate and incubated at 37 °C for 4 h with CCK-8 reagent (10 μL) (Dojindo, CK18, Japan). The absorbance was measured at a wavelength of 450 nm using an ELx800 plate reader (Thermo, Multiskan Spectrum, USA) to count the cells. Cell growth was represented as fold change from day 0 to day 4 and presented in a graph.

Cell invasion and migration studies were performed using a transwell assay. The upper chambers of a 24-well plate were filled with treated SW1783 cells (2 × 10^5 cells) and incubated for 48 h. To evaluate the invasive and migratory abilities of the cells, the top surface of the plate was pre-coated with a matrix gel solution (BD Biosciences, USA) or left uncoated. The remaining cells at the bottom layer were fixed with 4% paraformaldehyde and stained with 0.1% crystal violet (Solarbio, China) after removing the surface cells.

### Statistical analysis

All analyses were performed using R version 4.1.1, 64-bit, and its support package. To assess prognostic value and compare patient survival in various subgroups within each data set, Kaplan–Meier survival analysis and log-rank tests were performed. For significance tests comparing the various groups, Kruskal–Wallis and Wilcoxon’s tests were performed. By displaying univariate and multivariate forest plots, we examined if this created ERG signature is an independent predictive factor in comparison to other clinical features. The “stats” package and “prcomp” function were used to perform principal component analysis. Spearman correlation analysis was used to investigate the correlation coefficients. In all statistical investigations, *P* < 0.05 was considered statistically significant.

## Results

### Genetic and transcriptional alterations of ERGs in GBM

**Fig. 2:**
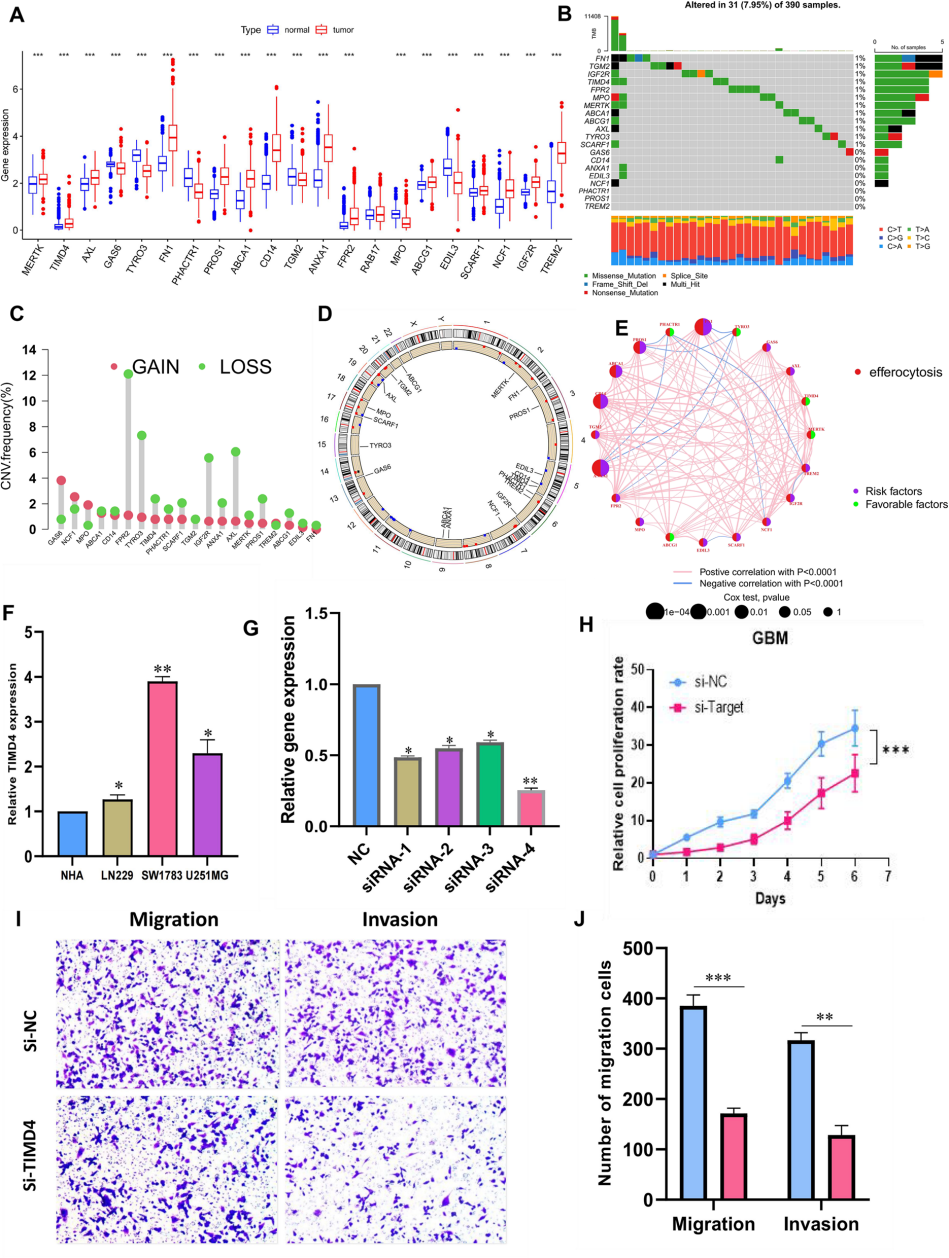
Genetic and transcriptional alterations of ERGs in GBM. **A** Distribution of expression of ERGs between normal and GBM. **B** Mutation frequency of 20 ERGs in the TCGA cohort of 390 GBM patients. **C** Copy number variation (CNV) of 20 ERGs in TCGA-GBM. **D** Localization of 20ERGs in chromosomal regions. **E** Network plot showing the correlation between the 20 ERGs. Red connecting lines indicate positive correlations, while blue indicates negative correlations. **F** TIMD4 was highly expressed in GBM cell lines compared to normal human astrocyte NHA cell lines. **G** RT-qPCR was performed to detect the relative expression of TIMD4 in GBM cells transfected with si-RNAs or negative control (NC). **H** CCK8 assay showed that SW1783 cells with reduced TIMD4 expression had significantly reduced proliferative capacity compared to the NC group. **I**, **J** Transwell assay showed that down-regulation of TIMD4 expression inhibited the migration and invasion ability of SW1783 cells. All data are expressed as mean ± SD of three independent experiments. * *p* < 0.05, ** *p* < 0.01, *** *p* < 0.001

In this study, we collected a total of 22 ERGs, 21 of which were identified in the TCGA and CGGA cohorts. Initially, we compared the expression levels of ERGs between GBM and normal tissues using TCGA, CGGA, and GTEx expression profiles. Our analysis showed that all ERGs, except RAB17, had different expression levels between tumor and normal tissues (Fig. 2A). We also examined the somatic mutation frequencies of the 20 differentially expressed ERGs and found that they had low mutation frequencies, with only 31 out of 390 GBM samples (7.95%) having mutations in these genes (Fig. 2B). In addition, somatic copy number variation analysis showed a general decrease in copy number variation (CNV) for genes such as FPR2, TYRO3, IGF2R and AXL, while GAS6 showed a gain in CNV (Fig. 2C). The chromosomal localization of these ERGs is shown in Fig. 2D. We constructed an efferocytosis-related network to demonstrate the comprehensive landscape of ERG interactions, regulator connections, and their prognostic value in patients with GBM (Fig. 2E). Additionally, we compared the expression levels of TIMD4 in HA cells and three GBM cell lines by cell line experiments and found that BARD1 was significantly highly expressed in tumor cells, especially SW1783 cells (Fig. 2F). We then examined the expression level of TIMD4 5 days after transfection by qRT-PCR to test the effectiveness of siRNA knockdown of TIMD4 in SW1783 cell lines (Fig. 2G). Subsequent CCK-8 cell assays showed that knockdown of BARD1 significantly reduced the proliferative capacity of the SW1783 cell line (Fig. 2H). In addition, GBM cells transfected with si-TIMD4 exhibited weaker migratory invasion ability in transwell assays (Fig. 2I, J). Thus, TIMD4 is a pro-carcinogenic factor in GBM. These findings suggest that there are significant differences in the genetic profiles and expression levels of ERGs between GBM and control samples, and that ERGs may play a crucial role in the development of GBM.

### Validation of single-cell sequencing data

**Fig. 3:**
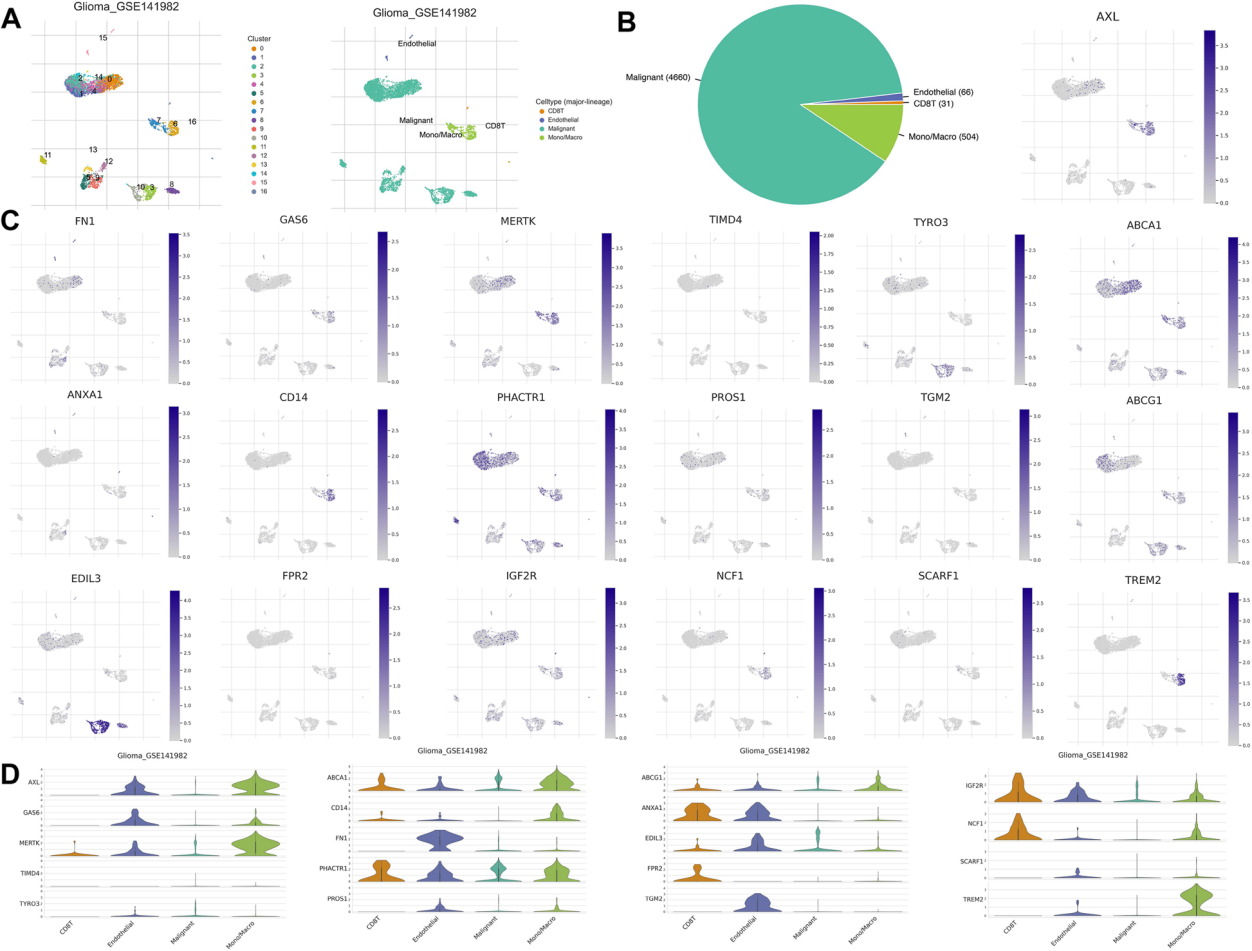
20 ERGs in single-cell RNA sequencing. **A**, **B** Annotation of all cell types in GSE141982 and the percentage of each cell type. **C**, **D** The expression of 20 ERGs in each cell type

To investigate the expression of 20 ERGs within the tumor microenvironment (TME), we analyzed the GBM single-cell dataset GSE141982 obtained from the TISCH database. Among the 20 ERGs, MPO expression was not detected. The GSE141982 dataset consisted of 16 cell clusters and 4 major cell types, which were distributed and counted as shown in Fig. 3A and B. Our analysis revealed that IGF2R, NCF1, and FPR2 were predominantly expressed in CD8T cells, with lower expression levels observed in malignant cells. Conversely, FN1 and GAS6 were primarily expressed in endothelial cells. Notably, our findings showed that almost all ERGs were associated with immune cell infiltration, indicating that efferocytosis plays a crucial role in the GBM immune microenvironment (Fig. 3C, D).

### Identification of ERG clusters

**Fig. 4:**
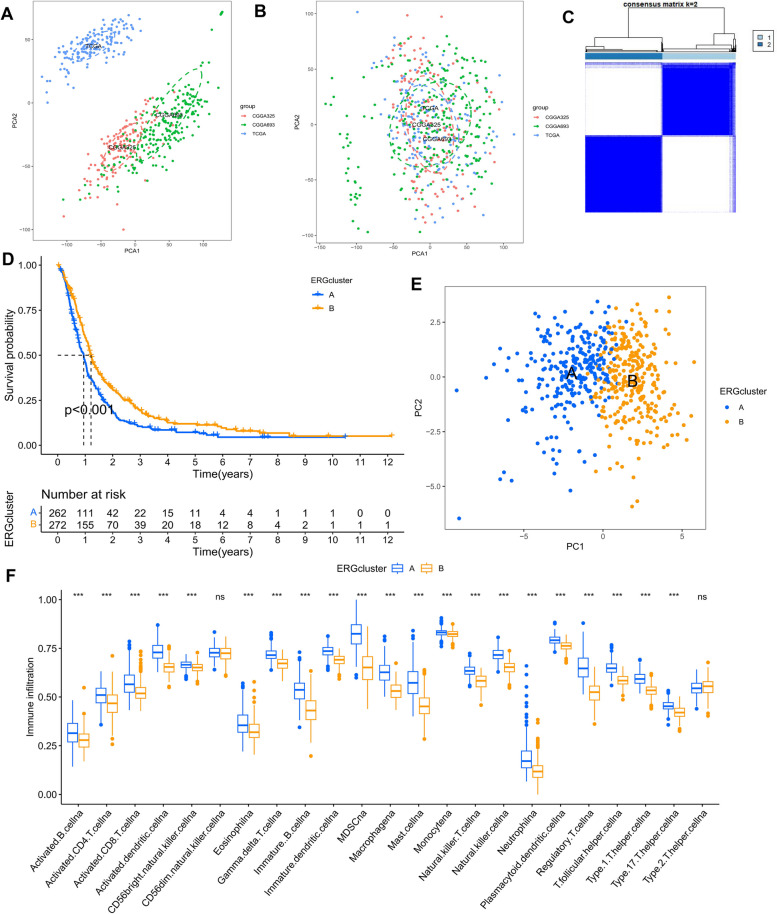
Identification of two ERG clusters. **A** Principal component analysis of common gene profiles before the combination of the TCGA-GBM, CGGA-325, and CGGA-693 cohorts. **B** Principal component analysis of common gene profiles after the combination of TCGA-GBM, CGGA-325, and CGGA-693 cohorts. **C** Heat map of the consensus matrix defining two clusters (k = 2) and their associated regions. **D** Kaplan–Meier survival analysis of OS in 2 ERG clusters. **E** PCA analysis shows significant differences in the transcriptome between the two clusters. **F** The abundance of tumor-infiltrating immune cells between two ERG clusters was calculated by ssGSEA. ns no significance, *** *p* < 0.001

We utilized the TCGA-GBM, CGG325, and CGGA-693 cohorts and performed PCA analysis to demonstrate a significant reduction in the corrected batch effect (Fig. 4A, B). The optimal number of clusters was determined to be k = 2, based on our findings, and we divided the 549 GBM patients into two clusters (Fig. 4C, Supplementary Figure S1, and Supplementary Table 3). Patients in cluster A had a worse prognosis than those in cluster B, as evidenced by Kaplan–Meier survival analysis (Fig. 4D, *P* < 0.001). Significant differences in transcriptional profiles between the two clusters were also observed using PCA analysis (Fig. 4E). We conducted the “ssGSEA” algorithm to explore the tumor microenvironment in both clusters, and the results indicated that cluster A had a higher abundance of immune cells than cluster B, except for CD56dim NK cells and type 2 helper T cells (Fig. 4F).

**Fig. 5:**
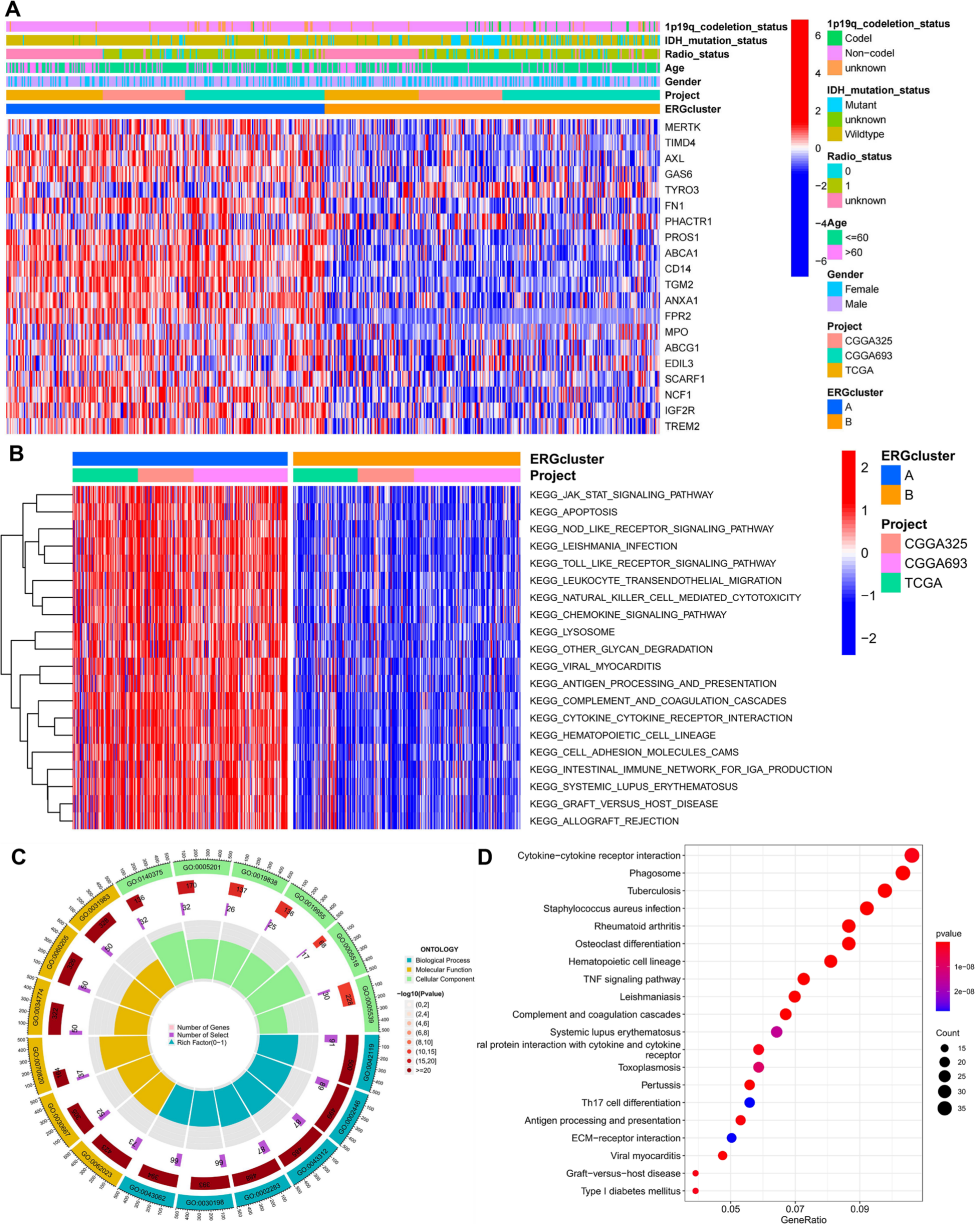
Clinicopathological and biological characteristics of two ERG clusters. **A** Differences in clinicopathological features and expression levels of ERG between the two different subtypes. **B** Differential enrichment of the KEGG pathway between the two ERG clusters based on GSVA analysis. **C**, **D** GO and KEGG enrichment analyses of DEGs among two ERG clusters

We then examined ERG expression and clinicopathological features in both clusters and found significant differences, with cluster A exhibiting significantly higher ERG expression (Fig. 5A). GSVA enrichment analysis revealed that immune activation-related pathways were significantly enriched in cluster A, including the JAK/STAT signaling pathway, programmed cell death, cytokine receptor interactions, chemokine signaling pathway activation, NOD-like, and Toll-like receptor signaling pathway (Fig. 5B). Differential expression analysis between the two clusters revealed 641 DEGs (Supplementary Table 4), which were enriched in functions related to efferocytosis and immune-related pathways such as neutrophil-mediated immunity, immune receptor activity, cytokine receptor activity, and leukocyte activation (Fig. 5C). KEEG enrichment analysis showed that these DEGs were associated with the progression of certain autoimmune diseases (Fig. 5D).

### Identification of ERG gene clusters

**Fig. 6:**
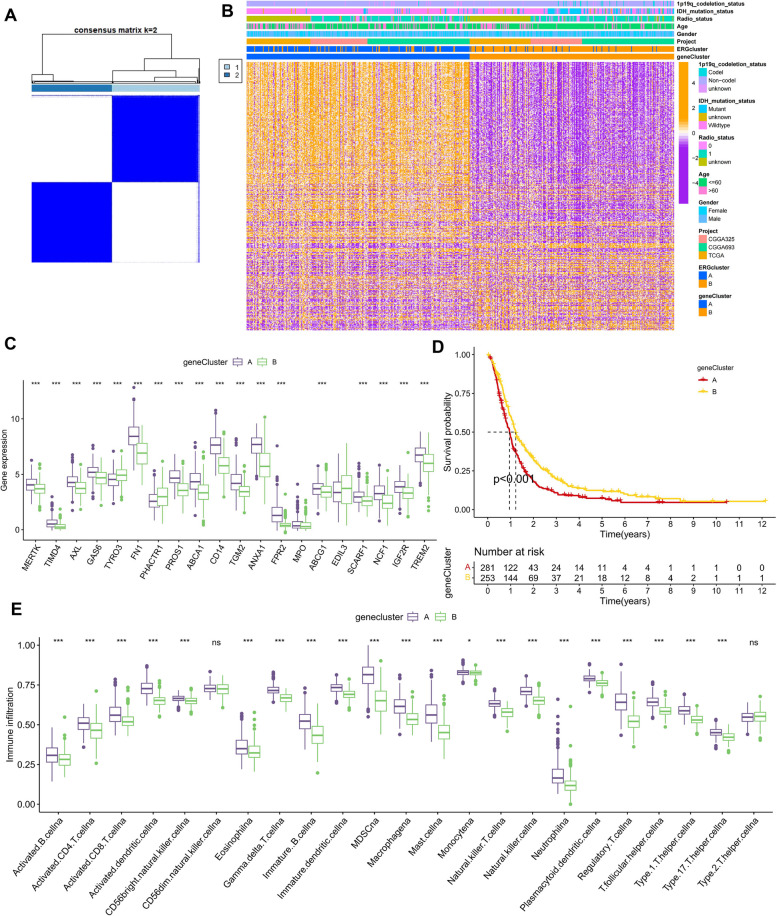
Identification and characterization of two ERG gene clusters. **A** Heat map of the consensus matrix defining the two gene clusters (k = 2) and their associated regions. **B** Relationship between clinicopathological features and the two gene clusters. **C** Expression differences of 20 ERGs between two ERG gene clusters. **D** Kaplan–Meier survival analysis of OS between two ERG gene clusters. **E** The abundance of tumor-infiltrating immune cells between two ERG gene clusters was calculated by ssGSEA. ns no significance,* *p* < 0.05, *** *p* < 0.001

To investigate the association between gene expression and GBM prognosis, we conducted univariate Cox regression analysis on the 641 DEGs and identified 296 genes that were prognostic for GBM (Supplementary Table 5). We then performed clustering analysis on these 296 DEGs, and found that the optimal number of clusters was 2 based on the slope of the cumulative distribution function curve (Fig. 6A, Supplementary Figure S2, and Supplementary Table 6). The heat map in Fig. 6B shows the expression of the 296 DEGs in the two gene clusters, as well as the clinicopathological characteristics of each sample. Remarkably, all 20 ERGs were more highly expressed in gene cluster A (Fig. 6C). Furthermore, Kaplan–Meier survival analysis revealed that patients in gene cluster A had a worse prognosis than those in gene cluster B (Fig. 6D, *p* < 0.001). To explore the tumor microenvironment of both gene clusters, we used the “ssGSEA” algorithm. Consistent with previous ERG clusters, differential enrichment of immune cells showed a higher abundance of immune cells in cluster A than in cluster B, except for CD56dim NK cells and type 2 helper T cells (Fig. 6E).

### Development of an efferocytosis-related scoring system

**Fig. 7:**
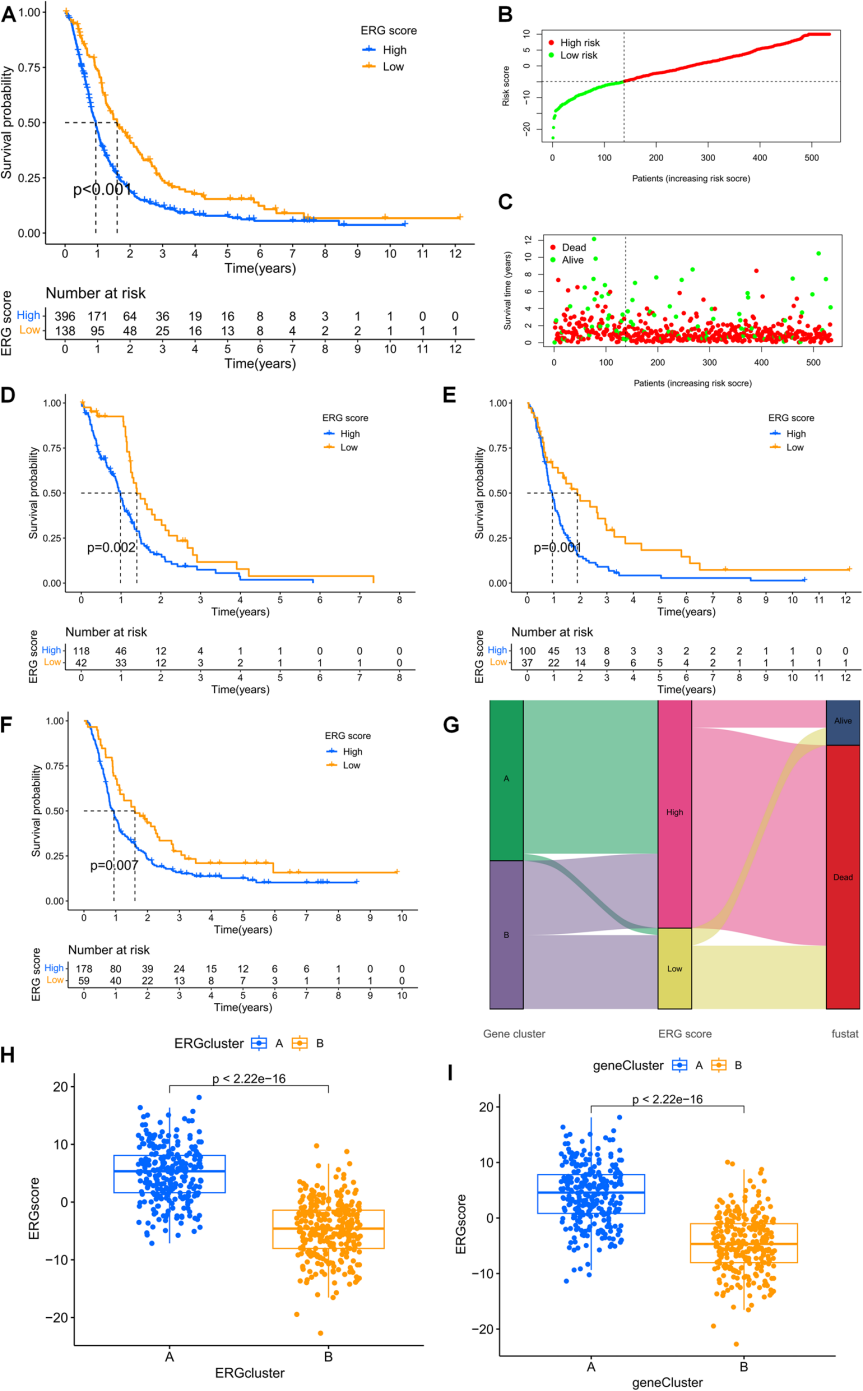
Construction and validation of the ERG scoring system. **A** Kaplan–Meier survival analysis of OS between high- and low-achieving subgroups in the entire cohort. **B** Distribution of ERG scores in the whole cohort. **C** Relationship between ERG characteristics and survival status in the whole cohort. **D** Kaplan–Meier survival analysis of OS between high and low-scoring subgroups in the TCGA-GBM cohort. **E** Kaplan–Meier survival analysis of OS between high- and low-achieving subgroups in the CGGA-325 cohort. **F** Kaplan–Meier survival analysis of OS between high-scoring and low-scoring subgroups in the CGGA-693 cohort. **G** Sankey diagram demonstrating the relationship between patient survival status and ERG group. **H** The difference in scores between the two ERG clusters. **I** The difference in scores of two ERG gene clusters

We identified 296 differentially expressed genes (DEGs) and categorized 54 genes that were positively correlated with the ERG gene cluster signature as gene signature A, while 242 genes that were negatively correlated were assigned to gene signature B (Figure S6). To identify candidate genes from these important genes, we employed the “Boruta” package, resulting in 53 genes for signature A and 202 genes for signature B (Supplementary Table 7). We then calculated ERG scores for 549 GBM patients using the PCA formula described earlier. After determining the best cut-off value for the PCA score, we divided the TCGA and CGGA cohorts into high-risk (HR) and low-risk (LR) groups. Significant differences in overall survival (OS) were observed between the two groups in the whole cohort and in three separate cohorts, with patients in the LR group often having a better prognosis (Fig. 7A, D, E, F). The risk curves and survival status plots emphasized the strong discriminatory power of this ERG signature (Fig. 7B, C). The Sankey plots showed that the majority of ERG gene cluster A with poorer prognosis belonged to the HR group, indicating poorer survival outcomes (Fig. 7G). Both ERG cluster A (Fig. 7H) and ERG gene cluster A (Fig. 7I) with poorer prognosis had higher ERG scores, further highlighting the prognostic value of this signature.

### Validation of an efferocytosis-related scoring system

**Fig. 8:**
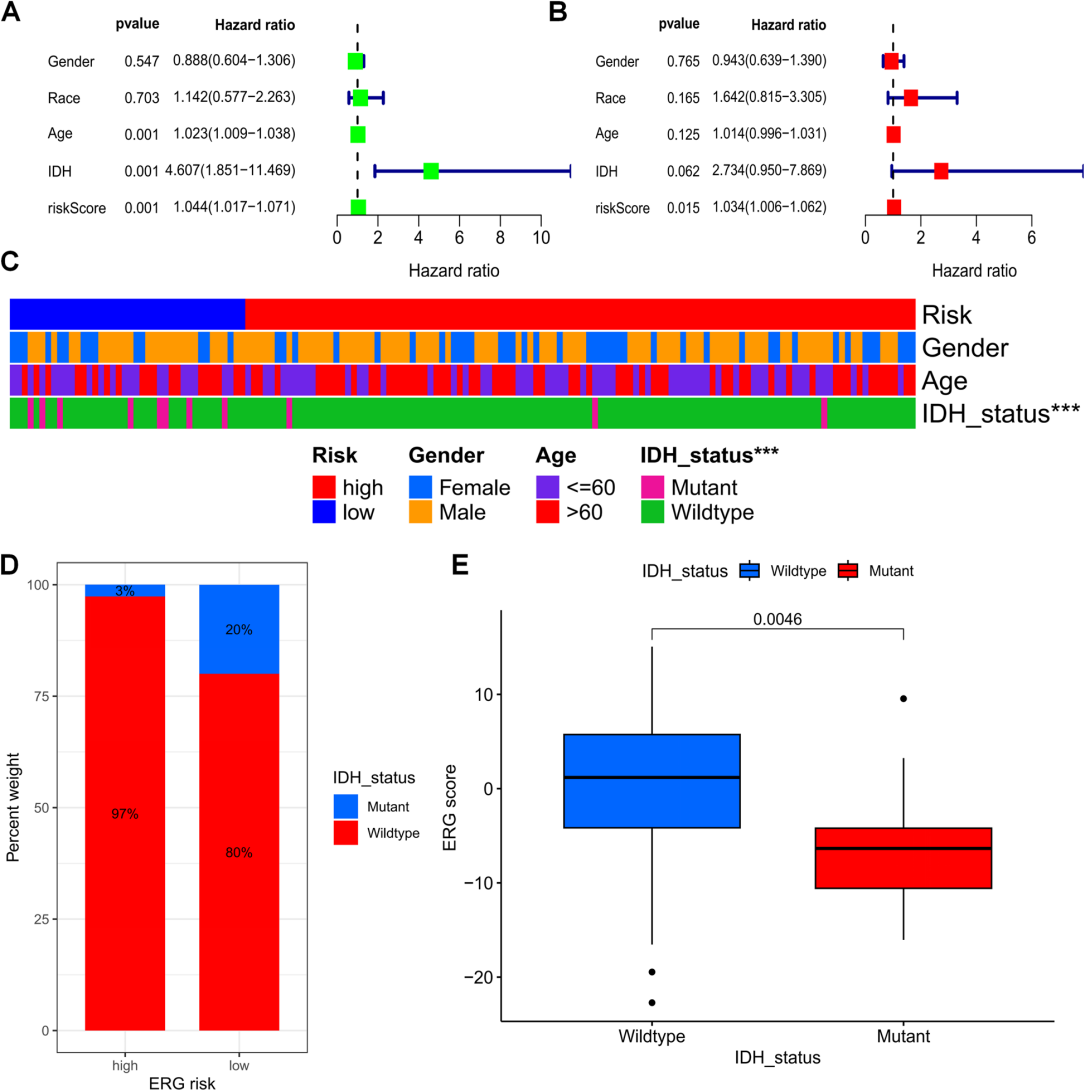
Prognostic value of ERG scores in patients with GBM. **A** Univariate and **B** multivariate COX analysis to assess ERG signature and clinical characteristics (including age, gender, and IDH mutation status). **C** Histogram of clinical characteristics associated with ERG scores. **D**, **E** Correlation of ERG scores with IDH mutation status. ***p < 0.001

After conducting univariate Cox regression analysis, we observed that age, IDH mutation status, and ERG score were significantly associated with OS in all datasets of GBM patients (Fig. 8A). Subsequently, we performed multivariate Cox regression analysis and identified that age and ERG score were independent prognostic indicators for GBM patients (Fig. 8B). Additionally, using the chi-square test, we found that our ERG grouping was associated with the gender and IDH mutation status of the patients (Fig. 8C), and that lower ERG scores were linked to IDH mutations (Fig. 8D, E). These findings suggest that our developed ERG signature is a reliable predictor of OS for GBM patients, irrespective of other clinical characteristics.

### The correlation with tumor microenvironment

**Fig. 9:**
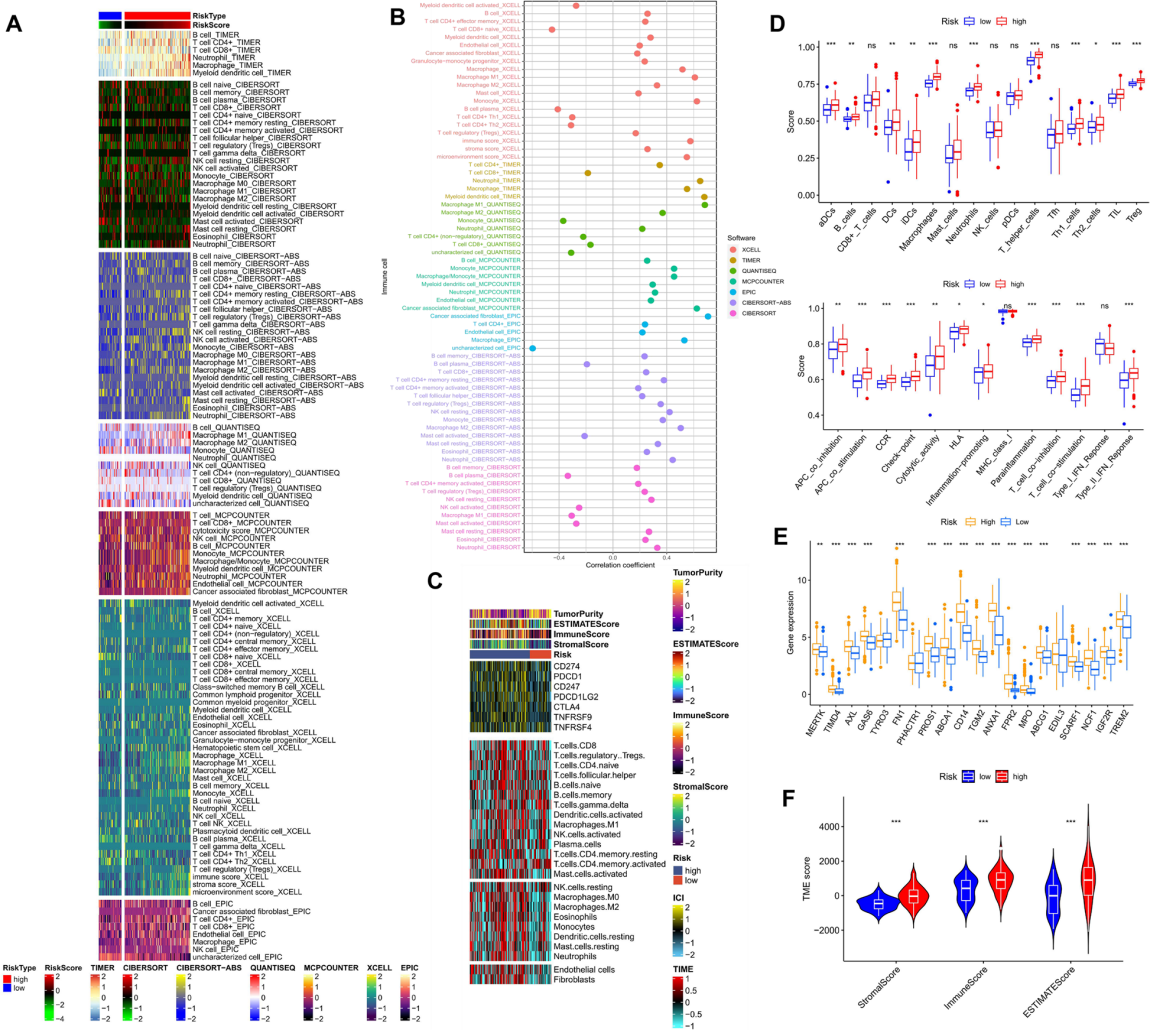
Analysis of the immune microenvironment in different risk groups. **A** Differences in immune infiltration status between different risk groups were evaluated by seven algorithms. **B** Bubble plot of the correlation between ERG score and immune cell abundance. **C** Heatmap showing differences in TME score, immune checkpoint expression, and immune cell infiltration calculated by ssGSEA among different risk subgroups. **D** Differences in ssGSEA scores of immune cells and immune function in the two score subgroups. **E** Differences in the expression of immune checkpoint genes in the two score groups. **F** Comparison of the differences in StromalScore, ImmuneScore, and ESTIMATEScore between the two score subgroups. ns no significance,* *p* < 0.05, ** *p* < 0.01, *** *p* < 0.001

In the tumor microenvironment (TME), immune cell infiltration plays a crucial role in immune response. Initially, we conducted Spearman correlation analysis in the TCGA cohort to examine the relationship between ERG scores and immune cell abundance in the GBM TME using different algorithms. Figure 9A illustrates the immune cell infiltration landscape in different risk groups, where we found that most immune cells were positively correlated with ERG scores. Interestingly, macrophage abundance linked to efferocytosis showed a positive correlation with ERG scores across all algorithms (Fig. 9B). To further investigate the association between ERG grouping and immune cells and functions, we quantified the ssGSEA enrichment scores for different immune cell subpopulations, related functions, or pathways. The results revealed that the high-scoring subgroups had more infiltration of B cells, CD8 + T cells, dendritic cells (DCs), immature DCs (IDCs), macrophages, neutrophils, plasmacytoid DCs (pDCs), helper T cells, type 1 T helper cells (Th1), type 2 T helper cells (Th2), tumor-infiltrating lymphocytes (TILs), and regulatory T cells (Tregs) (Fig. 9D). Similarly, all immune pathways, including APC_co_inhibition, Check-point, HLA, Inflammation-promoting, and T_cell_co-inhibition, were higher in the high-scoring subgroup (Fig. 9D). Furthermore, stromal scores, immune scores, and estimation scores were higher in the high-risk (HR) group (Fig. 9F). We also observed that some common immune checkpoints (ICs) were more highly expressed in the HR group, and we present the TME scores, immune checkpoint expression, and immune cell infiltration landscape between the two groups in the form of a heat map (Fig. 9C). Our findings in the CGGA-325 and CGGA-693 cohorts were similar to those discussed above (Supplementary Figure S3). Additionally, all 20 ERGs were more highly expressed in the HR group, suggesting that the higher overall immune level and immunogenicity of TME in the HR group were likely triggered by the effects of efferocytosis (Fig. 9E).

### Prediction and validation of immunotherapy efficacy, prediction of targeted chemotherapeutic drugs

**Fig. 10:**
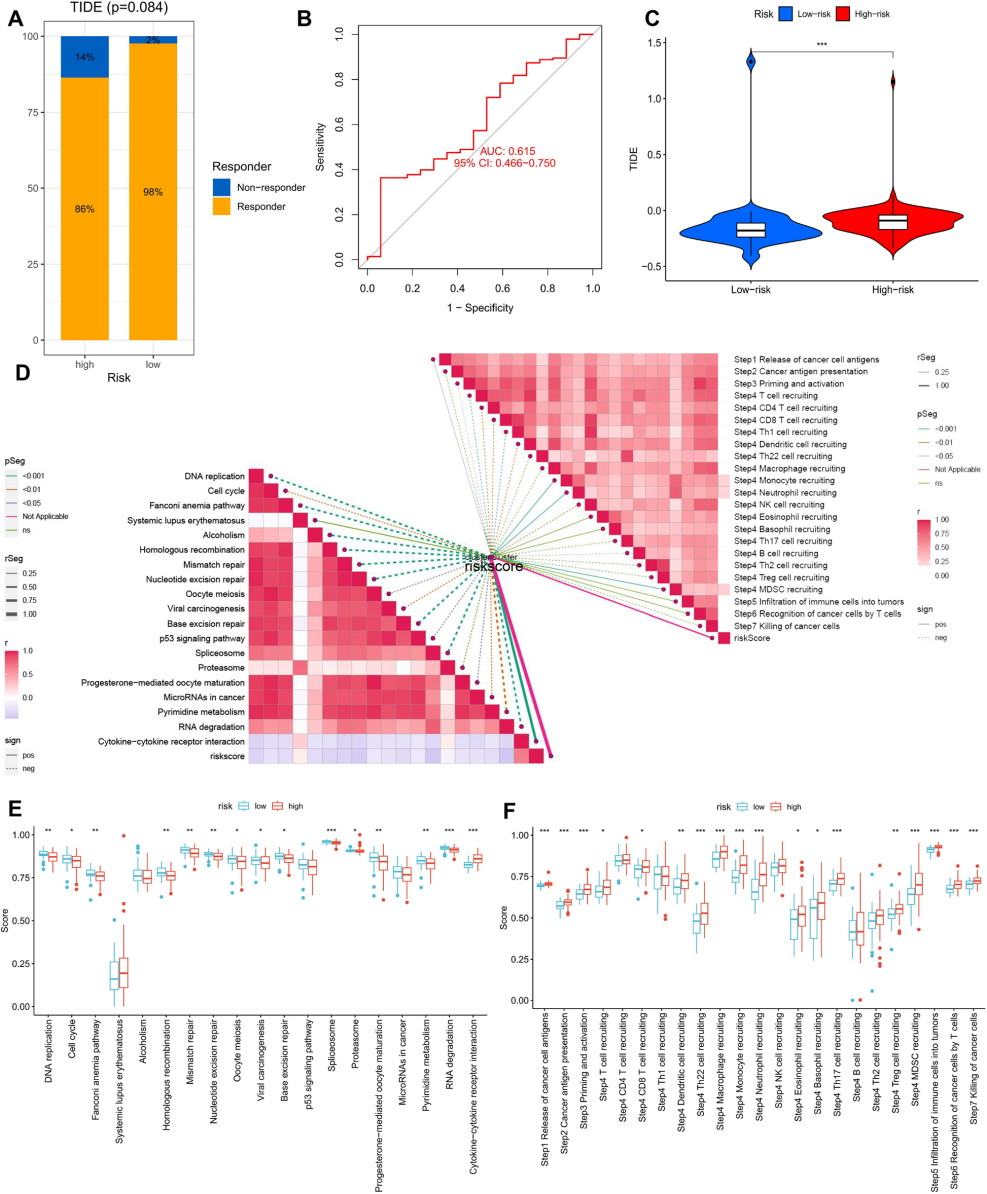
Prediction and validation of the effect of immunotherapy. **A** Distribution of ERG scores between responders and non-responders. **B** ERG score predicts ROC curve for immunotherapy response. **C** Distribution of TIDE scores between high- and low-risk groups in the TCGA-GBM dataset. **D** The relationship between ERG scores, ICB response traits, and each stage of the tumor immune cycle. **E** The plot of the difference in enrichment scores between the high-risk and low-risk groups on the immunotherapy prediction pathway. **F** The plot of differences between the high-risk and low-risk groups on each step of the cancer-immune cycle. * *p* < 0.05, ** *p* < 0.01, *** *p* < 0.001

**Fig. 11:**
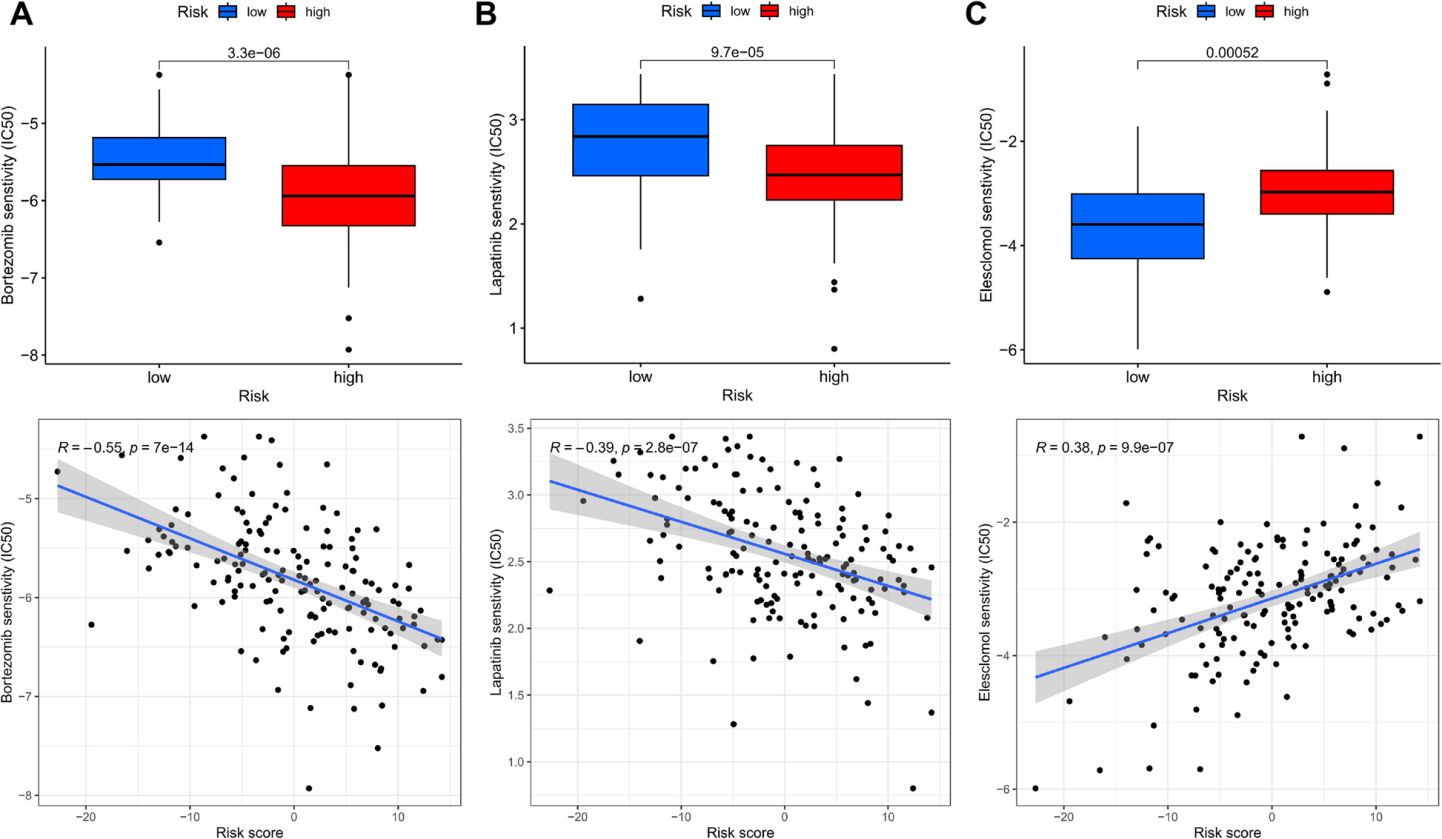
ERG signature predicts chemotherapy sensitivity. **A** Lapatinib, **B** Bortezomib, **C** Elesclomol

To determine the suitability of patients for immunotherapy, we utilized the TIDE score to assess potential immune dysfunctions in the tumor and regional lymph nodes. We found that patients in the low-risk subgroup were more likely to respond positively to immunotherapy (Fig. 10A, B, C). We also examined the sensitivity of three classical chemotherapeutic agents, Lapatinib, Bortezomib, and Elesclomol. In the low-risk group, patients treated with Lapatinib and Bortezomib had higher IC50 values, indicating greater sensitivity to these drugs. Conversely, patients treated with Elesclomol showed higher sensitivity in the high-risk group (Fig. 11A-C).

Furthermore, we investigated the differences between the two subgroups in predicting immune checkpoint blockade (ICB) response characteristics. We observed that the LR group had higher scores in DNA replication, cell cycle, viral oncogenesis, base excision repair, and p53 signaling pathway, compared to the HR group (Fig. 10E). We also evaluated the relationship between ERG scores and ICB-related positive signals and found a negative correlation between ERG scores and signals such as DNA replication, cell cycle, depletion pathway, mismatch repair, base excision repair, and microRNAs in cancer (Fig. 10D). To assess the biological function of the chemokine system and immunomodulators, we compared the differences in the activity of tumor immune steps between the high- and low-risk groups. We observed that the HR group exhibited upregulated activity in most steps of the tumor immune cycle, including the release of cancer cell antigens (step 1), the presentation of cancer antigens (step 2), priming and activation (step 3), and the entry of immune cells into the tumor (step 4), such as T cell recruitment, CD8 T cell recruitment, Th1 recruitment, DC cell recruitment, and Th22 cell recruitment (Fig. 10F). Additionally, we found a positive correlation between each of these steps in the tumor immune cycle and ERG scores (Fig. 10D).

## Discussion

GBMs can be categorized into different subgroups based on their gene expression profiles, which include mutations in isocitrate dehydrogenase (IDH), promoter methylation of O6-methylguanine-DNA methyltransferase (MGMT), and amplification of epidermal growth factor receptor (EGFR), reflecting their histological and morphological characteristics [42, 43]. However, relying solely on tumor size, histologic grade, or individual genetic features to predict prognosis and determine treatment options for GBM patients is insufficient due to the complex and multifactorial nature of GBM development. Thus, there is an urgent need for more accurate models for preclinical selection [29, 44].

Potential targets for cancer therapy include efferocytosis-related genes and pathways, such as phosphatidylserine, TYRO3, MerTK, indoleamine-2,3-dioxygenase 1, membrane-linked protein V, CD 47, TGF-β, and IL-10 [45]. These signals are often upregulated in GBM and associated with poor prognosis [46, 47]. However, treating glioma is complicated by tumor heterogeneity, changes in immune checkpoints, and extensive immunosuppression in the hypoxic microenvironment [48–50]. In order to address these challenges, Wu et al. proposed MerTK, an efferocytosis-related receptor, as a potential therapeutic target for glioblastoma [51]. In light of the crucial role that efferocytosis plays in GBM progression, along with immunosuppressive medication and promotion of tumor growth, a new efferocytosis-related scoring system was developed to evaluate the risk and predict personalized therapy [52].

Due to the high heterogeneity of glioblastoma (GBM), identifying various subtypes is often the best prognostic approach for patient intervention (with diverse phenotypes associated with efferocytosis). Despite efferocytosis and tumor development being popular topics in medical research, there is still insufficient literature on the combined effect of efferocytosis-related phenotypes in GBM. In this study, we categorized 549 GBM patients from three cohorts into two ERG clusters. Patients in cluster A had worse overall survival (OS) than those in cluster B, indicating that these efferocytosis-associated genes might affect GBM prognosis. To assess the difference in the tumor immune microenvironment, we compared the enrichment scores of tumor-infiltrating immune cells (TIICs) in the ERG clusters using ssGSEA. Despite having a worse prognosis, cluster A showed higher immune cell infiltration levels. The unique brain immunology contributes to GBM’s distinct tumor microenvironment, where multiple peripheral immune components, including various types of monocytes and lymphocytes, are present in the tumor immune microenvironment (TIME). However, their infiltration rate is significantly lower in gliomas than other tumors. GBM’s tumor-infiltrating lymphocytes (TILs) are low, while the content of CD4 + T cells and CD8 + T cells increases with tumor malignancy [53]. Treg cells also play an important role in the immunosuppressive microenvironment, as a component of the glioma microenvironment [54]. However, in advanced gliomas, TAMs are mainly characterized by an “M2” phenotype, which induces immunosuppressive responses and tumor immune escape [55, 56]. Although natural killer cells (NK cells) are potent innate cytotoxic lymphocytes, in the context of immunosuppression, tumor-associated neutrophils (TANs) play a crucial role. TANs, myeloid-derived suppressor cells (MDSCs), and the combined negative regulation of Treg and NK cells infiltrating in the TIME of GBM are generally considered functionally incompetent [57]. Our findings are consistent with GBM-derived cytokines and chemokines reprogramming TIICs. The distinct immune profiles of the two ERG clusters suggest that some underlying genes need to be identified. Therefore, we extracted differentially expressed genes (DEGs) from both and found that these genes were enriched in cytophagy and immune-related functions, indicating that they could be targeted in immunotherapy.

Using a bioinformatics approach, we developed an ERG signature for GBM based on the PCA algorithm and key DEGs to investigate the impact of efferocytosis-related phenotypes on prognosis. Our ERG signature effectively stratifies GBM patients based on risk and serves as an independent predictor of survival, with lower scores indicating better OS compared to higher scores. In line with previous findings, higher ERG scores are associated with increased tumor-infiltrating immune cells (TIICs) and poorer prognosis. Additionally, higher tumor microenvironment (TME) scores are linked to higher ERG scores. Several bioinformatics studies have shown that high mesenchymal and immune scores are associated with malignancy progression and a very poor prognosis [58]. Given that immunosuppressive cells within the TME can render immunotherapy ineffective, a high TME score is considered a red flag for GBM patients [59]. In addition to the suppressive role of TME, hypoxic conditions can also protect tumors from immune responses by inhibiting natural killer cell and connective tissue cell activity, and promoting immunosuppressive cytokine release and cell function enhancement. Our study shows that higher ERG scores are associated with an immunosuppressive microenvironment, highlighting the OS advantage of patients in lower-scoring groups.

The use of immune checkpoint inhibitors (ICIs) has revolutionized treatment for multiple types of cancer, including melanoma, lung cancer, and kidney cancer, resulting in a significant increase in overall survival for oncology patients [60]. ICIs targeting CTLA4 and PD-1/PDL-1 pathways have improved immune activation, paving the way for new therapies. Although the efficacy of immune checkpoint inhibition therapy for GBM is currently insufficient, ICIs are still the most clinically established form of immunotherapy [61]. Therefore, identifying patients who are likely to benefit from immunotherapy early on is crucial. Recently, a study demonstrated that administering PD-1 inhibitors two weeks before surgery, as a neoadjuvant regimen, improved overall survival in patients with recurrent GBM, compared to postoperative adjuvant therapy. This success supports the theory that PD-1 inhibitors can enhance antitumor immune responses [62, 63]. Nonetheless, current clinical practice lacks specific biomarkers for GBM immunotherapy.

We examined the expression of immune checkpoints (ICs) in high-risk (HR) and low-risk (LR) subgroups. In the HR group, most ICs (PD-1, CTLA-4, IDO, LAG-3, and TIM-3) were highly expressed. The interaction between PD-1 and PD-L1 generates an immune regulatory axis that promotes GBM cell invasion in brain tissue [63]. Elevated PD-L1 in glioma cells binds to PD-1 on tumor-associated macrophages (TAMs) and tumor-infiltrating lymphocytes (TILs), creating a suppressive immune microenvironment and resulting in a poor prognosis for GBM patients [64, 65]. Tumor-derived antigens increase LAG-3 expression, leading to CTL deficiency [66]. TIM-3 controls T-cell depletion by interacting with the ligand Gal-9 and contributes to tumor immune evasion [67]. Overexpression of TIM-3 in GBM is associated with worse prognosis, lower quality of life, and increased malignancy [68, 69]. The cancer immune cycle reflects the immunological response of the human immune system to cancer [70]. In our study of GBM tumor immune cycle and immune checkpoint blockade (ICB) response, we observed a significant positive correlation between ERG scores and ICB-related negative signals, as well as a positive correlation with the suppressive tumor immune cycle. These results further support the presence of immunosuppression and an inflammatory tumor microenvironment in the HR group. With the advent of bioinformatics, various algorithms have been successfully utilized to predict immunotherapy outcomes in tumors [31]. Using the TIDE algorithm, we explored the immunotherapeutic potential of our ERG signature and found significantly higher TIDE scores in the HR group. Lower ERG scores were linked to a better prognosis and higher response rates to immunotherapy.

Unlike inflammatory diseases, tumors employ efferocytosis to foster an immunosuppressive milieu that polarizes macrophages towards the M2 phenotype. This phenotype inhibits anti-tumor immunity, facilitates tissue repair, and stimulates vascular growth, ultimately leading to a dismal prognosis. Augmented expression of efferocytosis-related positive molecules boosts tumor cell survival, migration, invasion, and metastasis. Upon binding to its receptor, phosphatidylserine (PS) impedes the generation of NF-κB and type I IFN, thereby constraining the antitumor immune response.

## Conclusions

Despite offering valuable insights, our bioinformatics-based investigation has limitations that must be acknowledged. To confirm our findings, larger prospective studies and more in vivo and in vitro experiments are necessary, particularly for validating the efferocytosis-related signature in a genuine and larger cohort. Nevertheless, our study indicates that the ERG cluster signature may be associated with the prognosis and response to immunotherapy in GBM patients, and can direct future research on efferocytosis. Although the role of efferocytosis in tumor cell apoptosis is still in its preliminary stage of exploration, our study has successfully developed a signature linked to efferocytosis in GBM. In the future, our computational scoring method could help clinicians precisely assess the prognosis and immune status of GBM patients and recognize particular subgroups who may gain from tailored immunotherapy and chemotherapy treatments.

### Supplementary Information

**Additional file 1: Supplementary Figure 1.** Consensus unsupervised clustering of 549 GBM patients based on 8 ERGs. **Supplementary Figure 2.** Consensus unsupervised clustering of 549 GBM patients based on 473 DEGs. **Supplementary Figure 3.** Differences in tumor immune microenvironment between low and high-scoring subgroups in the CGGA cohort. **Supplementary Table 1.** 22 tumor-related ERGs were obtained from literature search results. **Supplementary Table 2.** siRNA and primer sequence information. **Supplementary Table 3.** Queue information was obtained for the two ERG clusters. **Supplementary Table 4.** Differential genes between two ERG clusters. **Supplementary Table 5.** Results after univariate Cox analysis of differential genes between two ERG clusters. **Supplementary Table 6.** Detailed cohort information for the 2 ERG gene clusters by the consensus clustering algorithm. **Supplementary Table 7.** Applying the “Boruta” package to screen for important genes.

## Data Availability

The datasets analyzed for this study were obtained from the UCSC Xena website (https://xenabrowser.net/datapages/) and CGGA dataset (http://www.cgga.org.cn/).
