# Supplementary material for: Interaction, immune infiltration characteristics and prognostic modeling of efferocytosis-related subtypes in glioblastoma
Source: BMC Med Genomics. 2023 Oct 18;16:248. doi: 10.1186/s12920-023-01688-4 (PMC10583324; doi:10.1186/s12920-023-01688-4)
Supplement: Supplementary file 1 — Additional file 1: Supplementary Figure 1. Consensus unsupervised clustering of 549 GBM patients based on 8 ERGs. Supplementary Figure 2. Consensus unsupervised clustering of 549 GBM patients based on 473 DEGs. Supplementary Figure 3. Differences in tumor immune microenvironment between low and high-scoring subgroups in the CGGA cohort. Supplementary Table 1. 22 tumor-related ERGs were obtained from literature search results. Supplementary Table 2. siRNA and primer sequence information. Supplementary Table 3. Queue information was obtained for the two ERG clusters. Supplementary Table 4. Differential genes between two ERG clusters. Supplementary Table 5. Results after univariate Cox analysis of differential genes between two ERG clusters. Supplementary Table 6. Detailed cohort information for the 2 ERG gene clusters by the consensus clustering algorithm. Supplementary Table 7. Applying the “Boruta” package to screen for important genes. [file 12920_2023_1688_MOESM1_ESM.pdf]

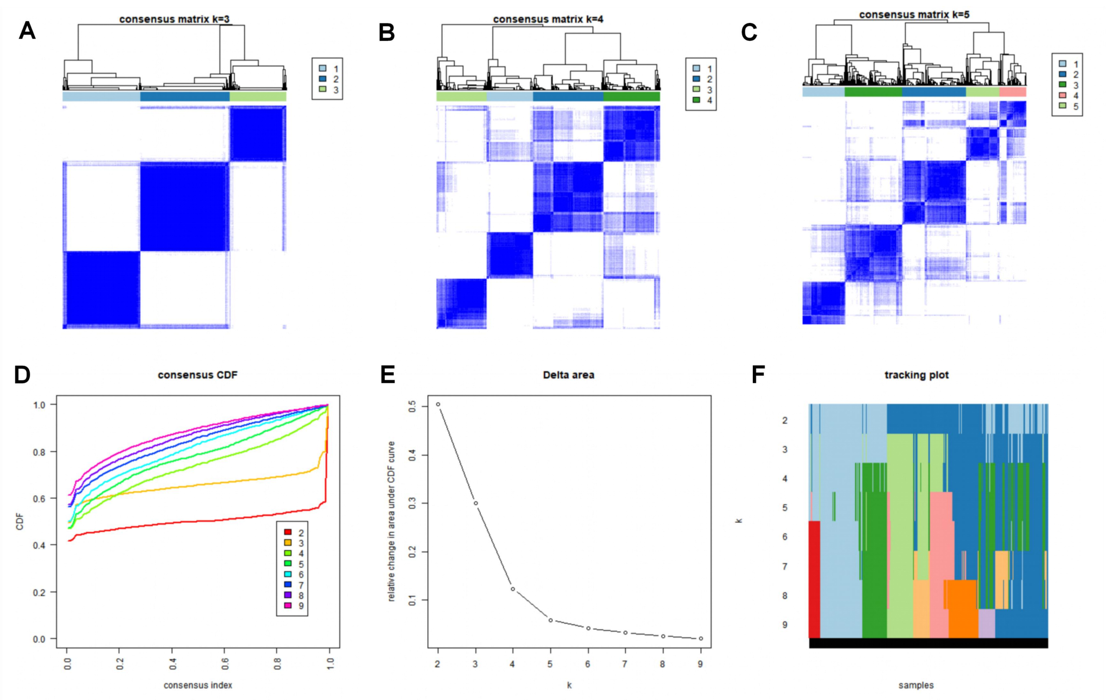

Supplementary Figure 1. Consensus unsupervised clustering of 549 GBM patients based on 8 ERGs. (A-C) Consensus matrix of 549 GBM patients when k=3, 4, and 5. (D) Relative change under the CDF curve when k=2-6. (E) Correlation between consensus index and CDF when k=2-9. (F) Tracking plots for 549 patients when k=2-9.

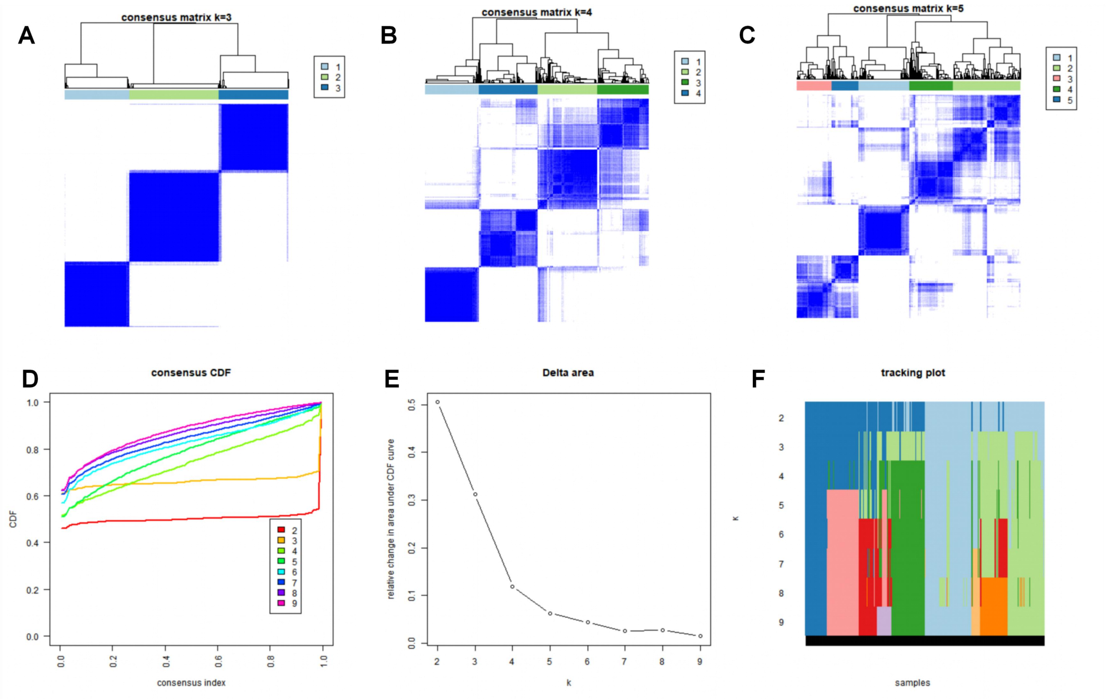

Supplementary Figure 2. Consensus unsupervised clustering of 549 GBM

patients based on 473 DEGs. (A-C) Consensus matrix of 549 GBM patients when k=3, 4, and 5. (D) Relative change under the CDF curve when k=2-6. (E) Correlation between consensus index and CDF when k=2-9. (F) Tracking plots for 549 patients when k=2-9.

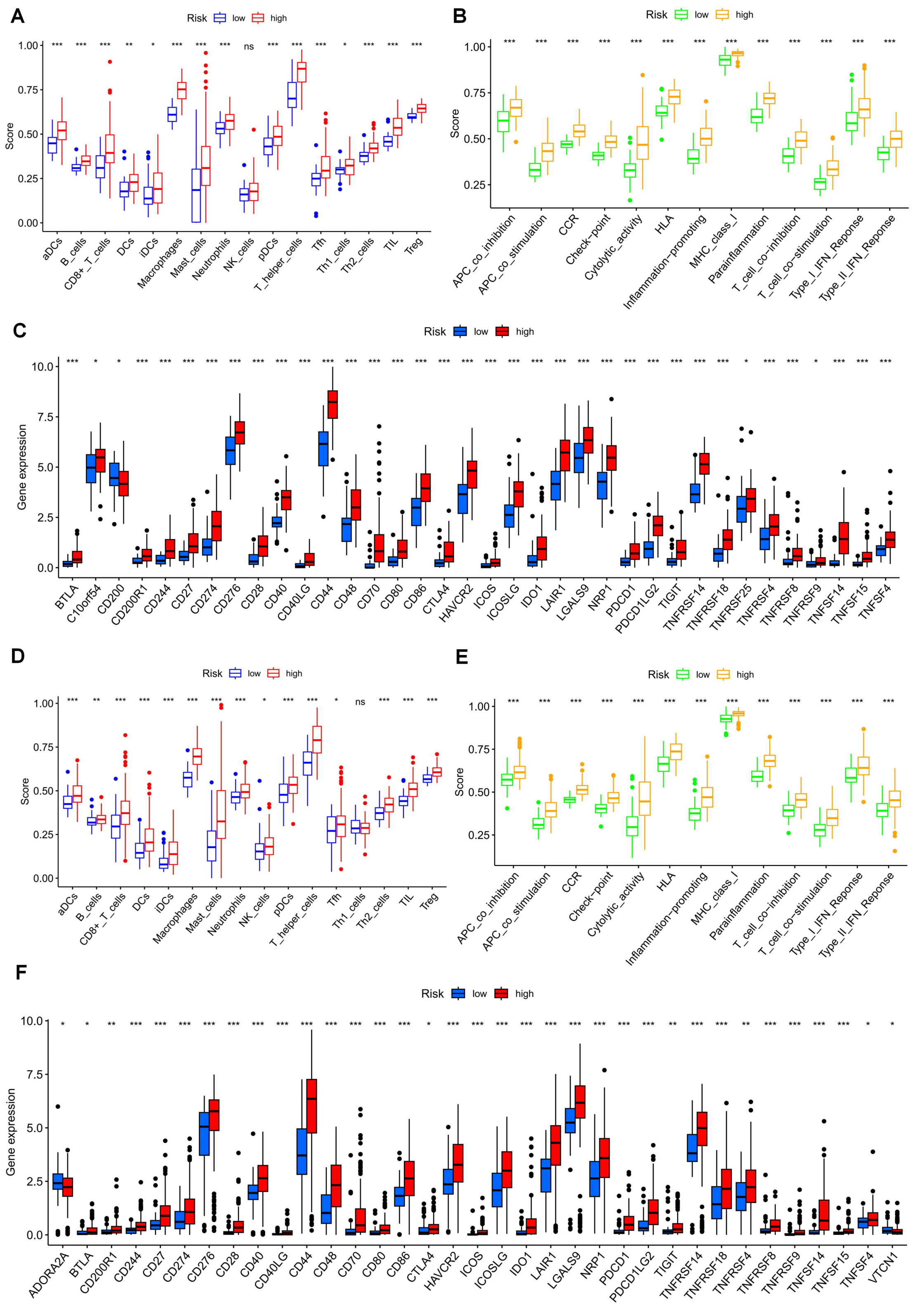

Supplementary Figure 3. Differences in tumor immune microenvironment between low and high-scoring subgroups in the CGGA cohort. (A, B) Differences in ssGSEA scores of immune cells and immune function in the two scoring subgroups of the CGGA-325 cohort. (C) Differences in immune checkpoint gene expression between the two scoring subgroups of the CGGA-325 cohort. (D, E) Differences in ssGSEA scores for immune cells and immune function in the two scoring subgroups of the CGGA-693 cohort. (F) Differences in immune checkpoint gene expression between the two scoring subgroups of the CGGA-693 cohort. * $p < 0.05$, ** p 0<0.01, **** $p < 0.0001$.

Supplementary Table 1. 22 tumor-related ERGs were obtained from literature search results

Genes/promoting efferocytosis	PMID	Localization of cells
MERTK	32049051	Macrophage surface
TIMD4	32640697	Macrophage surface
AXL	31088471	Macrophage surface
GAS6	31088471	
PLG	28320709	
TYRO3	31088471	Macrophage surface
FN1	30451988	
PHACTR1	33630758	
PROS1	29545796	Protein S
ABCA1	30429880	Macrophage intracellular
CD14	34593755	Macrophage surface
TGM2	27378395	Macrophage surface
ANXA1	33741356 26800869	
FPR2	33493655 35228119	
RAB17	28005073	Macrophage intracellular
MPO	32758447	
ABCG1	20431058	Macrophage intracellular
EDIL3	30455459	Secreted protein
SCARF1	35082161	
NCF1	34556485	
IGF2R	30657605	
TREM2	33845849	

Supplementary Table 2. siRNA and primer sequence information

siRNA	sense(5'-3')	antisense(5'-3')
TIMD4-Homo-409	GGCUGGUUCAACGAUGUAATT	UUACAUCGUUGAACCAGCCTT
TIMD4-Homo-622	GCCGUCUUCACAACAGCAATT	UUGCUGUUGUGAAGACGGCTT
TIMD4-Homo-1027	GGAUUUGUGCUCUUCGCAUTT	AUGCGAAGAGCACAAAUCCTT
TIMD4-Homo-793	GACACUGUCCUGCUGACAUTT	AUGUCAGCAGGACAGUGUCTT
**Primer**		
GAPDH	GGCCTCCAAGGAGTAAGACC (forward)	AGGGGAGATTCAGTGTGGTG (reverse)
TIMD4	GTACTGCTGCCGCATAGAAGT (forward)	TTGTTGTCATTTGTCGGGTGG (reverse)

Supplementary Table 3. Queue information was obtained for the two ERG clusters

ID	ERGcluster	ID	ERGcluster
TCGA_TCGA-19-1389	A	CGGA693_CGGA-1041	B
TCGA_TCGA-26-5132	A	CGGA693_CGGA-1075	A
TCGA_TCGA-28-2513	A	CGGA693_CGGA-1086	A
TCGA_TCGA-76-4927	A	CGGA693_CGGA-1103	B
TCGA_TCGA-26-5133	B	CGGA693_CGGA-1106	A
TCGA_TCGA-76-4928	A	CGGA693_CGGA-1130	B
TCGA_TCGA-06-5856	A	CGGA693_CGGA-1134	A
TCGA_TCGA-19-2619	B	CGGA693_CGGA-1135	A
TCGA_TCGA-41-3915	A	CGGA693_CGGA-1138	B
TCGA_TCGA-14-1825	B	CGGA693_CGGA-1142	B
TCGA_TCGA-15-1444	B	CGGA693_CGGA-1164	B
TCGA_TCGA-06-0210	A	CGGA693_CGGA-1172	B
TCGA_TCGA-14-1034	A	CGGA693_CGGA-120	B
TCGA_TCGA-28-2499	B	CGGA693_CGGA-1208	A
TCGA_TCGA-06-2570	B	CGGA693_CGGA-1236	B
TCGA_TCGA-06-2565	A	CGGA693_CGGA-1248	B
TCGA_TCGA-06-2559	A	CGGA693_CGGA-1255	A
TCGA_TCGA-26-5134	B	CGGA693_CGGA-1256	B
TCGA_TCGA-26-1442	B	CGGA693_CGGA-1257	B
TCGA_TCGA-12-3652	B	CGGA693_CGGA-1260	B
TCGA_TCGA-19-5960	B	CGGA693_CGGA-1262	A
TCGA_TCGA-06-2557	A	CGGA693_CGGA-1282	B
TCGA_TCGA-06-0686	B	CGGA693_CGGA-1325	A
TCGA_TCGA-19-2625	B	CGGA693_CGGA-1326	B

TCGA_TCGA-28-2514	B	CGGA693_CGGA-1337	B
TCGA_TCGA-06-2558	B	CGGA693_CGGA-1353	A
TCGA_TCGA-06-0882	A	CGGA693_CGGA-1354	B
TCGA_TCGA-28-5220	B	CGGA693_CGGA-1365	A
TCGA_TCGA-76-4929	A	CGGA693_CGGA-1371	A
TCGA_TCGA-19-2629	A	CGGA693_CGGA-1378	B
TCGA_TCGA-27-2528	B	CGGA693_CGGA-1380	A
TCGA_TCGA-06-1804	B	CGGA693_CGGA-1382	B
TCGA_TCGA-14-0817	A	CGGA693_CGGA-1387	B
TCGA_TCGA-06-0187	A	CGGA693_CGGA-139	B
TCGA_TCGA-28-5218	A	CGGA693_CGGA-1391	B
TCGA_TCGA-06-5408	A	CGGA693_CGGA-1392	A
TCGA_TCGA-28-5204	B	CGGA693_CGGA-1402	A
TCGA_TCGA-12-0618	B	CGGA693_CGGA-1403	A
TCGA_TCGA-32-2634	B	CGGA693_CGGA-1410	B
TCGA_TCGA-28-5207	B	CGGA693_CGGA-1415	A
TCGA_TCGA-28-5216	A	CGGA693_CGGA-1418	A
TCGA_TCGA-41-2571	B	CGGA693_CGGA-1419	B
TCGA_TCGA-12-1597	B	CGGA693_CGGA-1420	B
TCGA_TCGA-06-0219	B	CGGA693_CGGA-1422	A
TCGA_TCGA-06-5410	A	CGGA693_CGGA-1425	B
TCGA_TCGA-32-2638	A	CGGA693_CGGA-1426	A
TCGA_TCGA-14-0790	B	CGGA693_CGGA-1429	B
TCGA_TCGA-14-0787	A	CGGA693_CGGA-1430	B
TCGA_TCGA-06-0644	A	CGGA693_CGGA-1433	A
TCGA_TCGA-06-0744	B	CGGA693_CGGA-1441	A
TCGA_TCGA-06-0749	B	CGGA693_CGGA-1444	B
TCGA_TCGA-19-2620	A	CGGA693_CGGA-1451	A
TCGA_TCGA-32-2616	B	CGGA693_CGGA-1452	A
TCGA_TCGA-08-0386	B	CGGA693_CGGA-1457	A
TCGA_TCGA-06-0211	A	CGGA693_CGGA-1461	B
TCGA_TCGA-12-3653	B	CGGA693_CGGA-1462	A
TCGA_TCGA-27-2523	B	CGGA693_CGGA-1467	B
TCGA_TCGA-19-0957	B	CGGA693_CGGA-1472	B
TCGA_TCGA-12-0619	A	CGGA693_CGGA-1476	B
TCGA_TCGA-06-2569	B	CGGA693_CGGA-1478	B
TCGA_TCGA-06-0649	A	CGGA693_CGGA-1480	A
TCGA_TCGA-12-0616	B	CGGA693_CGGA-1481	A
TCGA_TCGA-06-2562	A	CGGA693_CGGA-1486	A
TCGA_TCGA-28-5213	A	CGGA693_CGGA-1491	B
TCGA_TCGA-14-1402	B	CGGA693_CGGA-1492	A
TCGA_TCGA-06-5858	A	CGGA693_CGGA-1494	A
TCGA_TCGA-41-2572	B	CGGA693_CGGA-1496	B
TCGA_TCGA-27-2519	A	CGGA693_CGGA-1498	A

TCGA_TCGA-19-2624	B	CGGA693_CGGA-1500	B
TCGA_TCGA-27-1830	A	CGGA693_CGGA-1501	B
TCGA_TCGA-06-0878	A	CGGA693_CGGA-1503	A
TCGA_TCGA-06-0646	A	CGGA693_CGGA-1505	A
TCGA_TCGA-06-0157	B	CGGA693_CGGA-1507	A
TCGA_TCGA-27-1835	B	CGGA693_CGGA-1520	B
TCGA_TCGA-27-2524	A	CGGA693_CGGA-1521	A
TCGA_TCGA-14-1829	B	CGGA693_CGGA-1529	B
TCGA_TCGA-76-4932	B	CGGA693_CGGA-1534	B
TCGA_TCGA-06-0190	A	CGGA693_CGGA-1535	A
TCGA_TCGA-06-0645	A	CGGA693_CGGA-1537	A
TCGA_TCGA-06-5411	B	CGGA693_CGGA-1538	B
TCGA_TCGA-06-2567	A	CGGA693_CGGA-1539	B
TCGA_TCGA-26-5139	A	CGGA693_CGGA-1541	B
TCGA_TCGA-32-5222	B	CGGA693_CGGA-1542	A
TCGA_TCGA-02-0047	B	CGGA693_CGGA-1543	A
TCGA_TCGA-14-0736	B	CGGA693_CGGA-1546	A
TCGA_TCGA-06-5859	A	CGGA693_CGGA-1548	A
TCGA_TCGA-27-2521	B	CGGA693_CGGA-1551	A
TCGA_TCGA-06-0743	B	CGGA693_CGGA-1558	A
TCGA_TCGA-14-1823	A	CGGA693_CGGA-1559	B
TCGA_TCGA-14-0781	A	CGGA693_CGGA-1560	B
TCGA_TCGA-06-0171	A	CGGA693_CGGA-1564	B
TCGA_TCGA-16-0846	B	CGGA693_CGGA-1571	B
TCGA_TCGA-06-0238	B	CGGA693_CGGA-1572	B
TCGA_TCGA-27-2526	B	CGGA693_CGGA-1586	A
TCGA_TCGA-28-2510	B	CGGA693_CGGA-1595	B
TCGA_TCGA-32-4213	A	CGGA693_CGGA-1596	B
TCGA_TCGA-06-0745	B	CGGA693_CGGA-1597	A
TCGA_TCGA-12-5295	B	CGGA693_CGGA-1601	A
TCGA_TCGA-27-1831	B	CGGA693_CGGA-1603	B
TCGA_TCGA-27-1832	A	CGGA693_CGGA-1604	B
TCGA_TCGA-06-0132	A	CGGA693_CGGA-1605	B
TCGA_TCGA-27-1834	A	CGGA693_CGGA-1611	B
TCGA_TCGA-28-1753	A	CGGA693_CGGA-1612	A
TCGA_TCGA-32-2615	A	CGGA693_CGGA-1613	A
TCGA_TCGA-32-1982	A	CGGA693_CGGA-1615	B
TCGA_TCGA-28-2509	A	CGGA693_CGGA-1624	B
TCGA_TCGA-06-0125	B	CGGA693_CGGA-1626	A
TCGA_TCGA-06-0158	A	CGGA693_CGGA-1631	A
TCGA_TCGA-14-0789	A	CGGA693_CGGA-1634	B
TCGA_TCGA-06-0174	B	CGGA693_CGGA-1635	B
TCGA_TCGA-28-5208	A	CGGA693_CGGA-1641	B
TCGA_TCGA-06-0141	A	CGGA693_CGGA-1643	B

TCGA_TCGA-06-0129	B	CGGA693_CGGA-1644	B
TCGA_TCGA-06-5416	B	CGGA693_CGGA-1650	B
TCGA_TCGA-32-1970	B	CGGA693_CGGA-1654	B
TCGA_TCGA-19-1787	A	CGGA693_CGGA-1656	B
TCGA_TCGA-06-0184	A	CGGA693_CGGA-1658	A
TCGA_TCGA-41-5651	B	CGGA693_CGGA-1659	A
TCGA_TCGA-02-2485	B	CGGA693_CGGA-1663	A
TCGA_TCGA-06-0138	A	CGGA693_CGGA-1666	A
TCGA_TCGA-06-0747	B	CGGA693_CGGA-1678	A
TCGA_TCGA-06-5414	A	CGGA693_CGGA-1681	A
TCGA_TCGA-06-0750	A	CGGA693_CGGA-1682	A
TCGA_TCGA-76-4925	B	CGGA693_CGGA-1684	A
TCGA_TCGA-06-0221	B	CGGA693_CGGA-1687	A
TCGA_TCGA-19-4065	A	CGGA693_CGGA-1690	A
TCGA_TCGA-06-0178	B	CGGA693_CGGA-1694	B
TCGA_TCGA-02-0055	A	CGGA693_CGGA-1697	B
TCGA_TCGA-02-2483	B	CGGA693_CGGA-1698	A
TCGA_TCGA-06-2563	B	CGGA693_CGGA-1699	B
TCGA_TCGA-06-0139	A	CGGA693_CGGA-1702	B
TCGA_TCGA-28-5209	B	CGGA693_CGGA-1706	A
TCGA_TCGA-02-2486	A	CGGA693_CGGA-1708	B
TCGA_TCGA-76-4931	B	CGGA693_CGGA-1709	A
TCGA_TCGA-06-2564	A	CGGA693_CGGA-1713	B
TCGA_TCGA-27-1837	B	CGGA693_CGGA-1722	A
TCGA_TCGA-41-4097	A	CGGA693_CGGA-1727	B
TCGA_TCGA-26-5136	A	CGGA693_CGGA-1728	A
TCGA_TCGA-15-0742	B	CGGA693_CGGA-1729	B
TCGA_TCGA-06-5417	B	CGGA693_CGGA-1735	B
TCGA_TCGA-26-5135	B	CGGA693_CGGA-1736	A
TCGA_TCGA-06-5418	A	CGGA693_CGGA-1740	B
TCGA_TCGA-28-5215	A	CGGA693_CGGA-1744	A
TCGA_TCGA-06-0152	A	CGGA693_CGGA-1749	B
TCGA_TCGA-19-1390	B	CGGA693_CGGA-1750	B
TCGA_TCGA-32-1980	A	CGGA693_CGGA-1758	B
TCGA_TCGA-06-0168	A	CGGA693_CGGA-1764	B
TCGA_TCGA-06-5412	A	CGGA693_CGGA-1767	A
TCGA_TCGA-32-2632	A	CGGA693_CGGA-1769	B
TCGA_TCGA-12-3650	B	CGGA693_CGGA-1770	B
TCGA_TCGA-06-0156	A	CGGA693_CGGA-1773	A
TCGA_TCGA-06-0130	A	CGGA693_CGGA-1776	B
TCGA_TCGA-06-5413	A	CGGA693_CGGA-1780	A
TCGA_TCGA-12-0821	B	CGGA693_CGGA-1785	B
TCGA_TCGA-12-5299	A	CGGA693_CGGA-1807	B
TCGA_TCGA-06-2561	A	CGGA693_CGGA-1811	A

TCGA_TCGA-14-0871	B	CGGA693_CGGA-1812	A
TCGA_TCGA-14-2554	B	CGGA693_CGGA-1814	A
TCGA_TCGA-28-1747	B	CGGA693_CGGA-1815	A
TCGA_TCGA-16-1045	A	CGGA693_CGGA-1817	A
TCGA_TCGA-76-4926	B	CGGA693_CGGA-1819	B
CGGA325_CGGA-1001	A	CGGA693_CGGA-1820	B
CGGA325_CGGA-1007	B	CGGA693_CGGA-1826	B
CGGA325_CGGA-1011	A	CGGA693_CGGA-1833	B
CGGA325_CGGA-1015	A	CGGA693_CGGA-1840	B
CGGA325_CGGA-1023	B	CGGA693_CGGA-1857	A
CGGA325_CGGA-1024	B	CGGA693_CGGA-1865	A
CGGA325_CGGA-1026	A	CGGA693_CGGA-1866	B
CGGA325_CGGA-1035	A	CGGA693_CGGA-1870	B
CGGA325_CGGA-1039	B	CGGA693_CGGA-1886	B
CGGA325_CGGA-1045	A	CGGA693_CGGA-1899	B
CGGA325_CGGA-1049	A	CGGA693_CGGA-1901	A
CGGA325_CGGA-1072	B	CGGA693_CGGA-1908	B
CGGA325_CGGA-1077	A	CGGA693_CGGA-1911	B
CGGA325_CGGA-1083	A	CGGA693_CGGA-1912	B
CGGA325_CGGA-1091	B	CGGA693_CGGA-1916	B
CGGA325_CGGA-1109	B	CGGA693_CGGA-1946	A
CGGA325_CGGA-1124	A	CGGA693_CGGA-1953	A
CGGA325_CGGA-1139	B	CGGA693_CGGA-1955	B
CGGA325_CGGA-1171	A	CGGA693_CGGA-1972	A
CGGA325_CGGA-1180	A	CGGA693_CGGA-1976	A
CGGA325_CGGA-1214	B	CGGA693_CGGA-1985	B
CGGA325_CGGA-1216	B	CGGA693_CGGA-2003	B
CGGA325_CGGA-1224	A	CGGA693_CGGA-2008	A
CGGA325_CGGA-1234	A	CGGA693_CGGA-2024	B
CGGA325_CGGA-1237	B	CGGA693_CGGA-2038	B
CGGA325_CGGA-1240	A	CGGA693_CGGA-2039	B
CGGA325_CGGA-1251	B	CGGA693_CGGA-2047	A
CGGA325_CGGA-1258	A	CGGA693_CGGA-2053	B
CGGA325_CGGA-1270	A	CGGA693_CGGA-2056	A
CGGA325_CGGA-1299	B	CGGA693_CGGA-2062	A
CGGA325_CGGA-1313	A	CGGA693_CGGA-2075	A
CGGA325_CGGA-1320	B	CGGA693_CGGA-2078	A
CGGA325_CGGA-1332	B	CGGA693_CGGA-2082	A
CGGA325_CGGA-1338	A	CGGA693_CGGA-2088	A
CGGA325_CGGA-1342	A	CGGA693_CGGA-2106	B
CGGA325_CGGA-274	A	CGGA693_CGGA-2115	B
CGGA325_CGGA-413	B	CGGA693_CGGA-487	A
CGGA325_CGGA-483	A	CGGA693_CGGA-509	B
CGGA325_CGGA-494	B	CGGA693_CGGA-530	A

CGGA325_CGGA-499	B
CGGA325_CGGA-525	A
CGGA325_CGGA-604	A
CGGA325_CGGA-658	A
CGGA325_CGGA-676	A
CGGA325_CGGA-679	A
CGGA325_CGGA-680	B
CGGA325_CGGA-700	B
CGGA325_CGGA-731	A
CGGA325_CGGA-782	A
CGGA325_CGGA-789	A
CGGA325_CGGA-802	A
CGGA325_CGGA-804	B
CGGA325_CGGA-808	B
CGGA325_CGGA-837	B
CGGA325_CGGA-842	A
CGGA325_CGGA-848	A
CGGA325_CGGA-850	B
CGGA325_CGGA-859	A
CGGA325_CGGA-876	A
CGGA325_CGGA-878	B
CGGA325_CGGA-902	A
CGGA325_CGGA-D03	B
CGGA325_CGGA-D09	B
CGGA325_CGGA-D35	A
CGGA325_CGGA-D37	A
CGGA325_CGGA-D57	A
CGGA325_CGGA-342	A
CGGA325_CGGA-1219	B
CGGA325_CGGA-1287	B
CGGA325_CGGA-1314	B
CGGA325_CGGA-1384	B
CGGA325_CGGA-1409	B
CGGA325_CGGA-491	B
CGGA325_CGGA-710	A
CGGA325_CGGA-747	B
CGGA325_CGGA-761	B
CGGA325_CGGA-1008	B
CGGA325_CGGA-1053	A
CGGA325_CGGA-1070	B
CGGA325_CGGA-1073	A
CGGA325_CGGA-1074	A
CGGA325_CGGA-1114	A
CGGA325_CGGA-1275	A
CGGA693_CGGA-568	A
CGGA693_CGGA-777	A
CGGA693_CGGA-831	A
CGGA693_CGGA-869	A
CGGA693_CGGA-D53	B
CGGA693_CGGA-P100	B
CGGA693_CGGA-P102	B
CGGA693_CGGA-P106	B
CGGA693_CGGA-P109	B
CGGA693_CGGA-P112	B
CGGA693_CGGA-P116	B
CGGA693_CGGA-P136	B
CGGA693_CGGA-P143	A
CGGA693_CGGA-P15	B
CGGA693_CGGA-P154	B
CGGA693_CGGA-P16	B
CGGA693_CGGA-P160	A
CGGA693_CGGA-P164	A
CGGA693_CGGA-P175	A
CGGA693_CGGA-P178	B
CGGA693_CGGA-P180	A
CGGA693_CGGA-P182	A
CGGA693_CGGA-P199	A
CGGA693_CGGA-P205	A
CGGA693_CGGA-P22	B
CGGA693_CGGA-P25	A
CGGA693_CGGA-P28	A
CGGA693_CGGA-P280	B
CGGA693_CGGA-P283	A
CGGA693_CGGA-P295	B
CGGA693_CGGA-P335	B
CGGA693_CGGA-P385	B
CGGA693_CGGA-P401	A
CGGA693_CGGA-P411	B
CGGA693_CGGA-P415	A
CGGA693_CGGA-P499	A
CGGA693_CGGA-P512	B
CGGA693_CGGA-P585	A
CGGA693_CGGA-P596	B
CGGA693_CGGA-P609	B
CGGA693_CGGA-P610	B
CGGA693_CGGA-P619	A
CGGA693_CGGA-P625	A
CGGA693_CGGA-P7	A

CGGA325_CGGA-759	A
CGGA325_CGGA-D30	A
CGGA325_CGGA-1019	A
CGGA325_CGGA-1078	A
CGGA325_CGGA-1177	A
CGGA325_CGGA-1271	B
CGGA325_CGGA-1285	A
CGGA325_CGGA-1343	B
CGGA325_CGGA-1370	B
CGGA325_CGGA-624	A
CGGA325_CGGA-669	B
CGGA325_CGGA-D02	A
CGGA325_CGGA-D26	A
CGGA325_CGGA-D32	B
CGGA325_CGGA-D36	A
CGGA325_CGGA-D51	A
CGGA325_CGGA-1060	B
CGGA325_CGGA-1227	B
CGGA325_CGGA-1381	B
CGGA325_CGGA-1394	B
CGGA325_CGGA-545	A
CGGA325_CGGA-899	B
CGGA325_CGGA-D38	B
CGGA325_CGGA-J100	B
CGGA325_CGGA-1116	B
CGGA325_CGGA-1119	B
CGGA325_CGGA-1188	B
CGGA325_CGGA-1218	A
CGGA325_CGGA-1272	B
CGGA325_CGGA-1283	B
CGGA325_CGGA-1301	A
CGGA693_CGGA-P87	B
CGGA693_CGGA-P89	A
CGGA693_CGGA-P99	B
CGGA325_CGGA-1346	A
CGGA325_CGGA-1393	A
CGGA325_CGGA-272	A
CGGA325_CGGA-318	A
CGGA325_CGGA-773	B
CGGA325_CGGA-719	B
CGGA325_CGGA-1068	A
CGGA325_CGGA-1105	A
CGGA325_CGGA-1129	B
CGGA325_CGGA-1136	A
CGGA325_CGGA-1170	B
CGGA325_CGGA-1175	B
CGGA325_CGGA-1324	B
CGGA325_CGGA-1375	B
CGGA325_CGGA-1412	B
CGGA325_CGGA-374	B
CGGA325_CGGA-822	B
CGGA325_CGGA-D34	B
CGGA325_CGGA-D59	B
CGGA325_CGGA-518	B
CGGA325_CGGA-1099	A
CGGA325_CGGA-1197	B
CGGA325_CGGA-1450	B
CGGA325_CGGA-1460	B
CGGA325_CGGA-1475	A
CGGA693_CGGA-1017	B
CGGA693_CGGA-1036	A

Supplementary Table 4. Differential genes between two ERG clusters

id	logFC	AveExpr	t	P.Value	adj.P.Val	B
LAIR1	-1.606705504	4.267799945	-25.94546421	1.49E-97	2.62E-93	211.8064579
SIGLEC9	-1.348689363	2.721477093	-25.42269486	6.77E-95	5.93E-91	205.7235381
FCGR2A	-1.68105445	4.775874424	-24.972454	1.33E-92	7.74E-89	200.475817
CD14	-2.028188739	6.71987014	-24.85547301	5.23E-92	2.29E-88	199.1112388
CD4	-1.348395518	4.71753638	-24.53296057	2.30E-90	8.06E-87	195.3471175

LRRC25	-1.38451502	3.009349744	-24.12081835	2.91E-88	8.50E-85	190.5333617
CTSB	-1.474106823	8.700553422	-23.7359901	2.68E-86	6.70E-83	186.0361335
FCGR3A	-1.796221822	6.233356863	-23.21632262	1.20E-83	2.63E-80	179.9613872
CD163	-2.556197868	5.071335964	-23.09775849	4.84E-83	9.43E-80	178.5754026
ARPC1B	-1.307671508	6.315545535	-22.97855584	1.97E-82	3.44E-79	177.1820294
IFI30	-1.747699349	4.935769114	-22.80664059	1.48E-81	2.36E-78	175.1727029
TNFRSF1B	-1.446589281	4.173690805	-22.74746272	2.97E-81	4.34E-78	174.4811142
MSR1	-1.759543258	4.123766795	-22.54974199	3.03E-80	4.08E-77	172.1707952
HCLS1	-1.359102357	5.018288624	-22.50234848	5.29E-80	6.61E-77	171.6171111
SNX20	-1.067021852	1.688963455	-22.24048619	1.14E-78	1.34E-75	168.558698
SERPINA1	-1.849343231	4.661875585	-22.21884655	1.47E-78	1.61E-75	168.3060308
THEMIS2	-1.391912154	4.223714527	-22.17083113	2.59E-78	2.55E-75	167.7454404
SIGLEC7	-1.300549706	2.270490905	-22.16993038	2.62E-78	2.55E-75	167.7349246
ITGB2	-1.600969939	5.811938331	-22.07878975	7.63E-78	7.04E-75	166.6710158
PLAUR	-1.77790674	4.22528151	-22.0015839	1.89E-77	1.65E-74	165.7699629
CCR5	-1.123859507	1.73436631	-21.94035632	3.87E-77	3.23E-74	165.0555224
CTSZ	-1.765498479	7.108643192	-21.87551712	8.28E-77	6.59E-74	164.2990748
NCKAP1L	-1.226913618	3.223458971	-21.85775863	1.02E-76	7.77E-74	164.0919202
IL4R	-1.191390888	3.520764193	-21.75341063	3.47E-76	2.53E-73	162.8749204
ARHGAP30	-1.2398112	3.08765073	-21.69781832	6.65E-76	4.66E-73	162.2267201
SLC11A1	-1.884291492	4.377258652	-21.6205957	1.64E-75	1.11E-72	161.3265163
DOK3	-1.223218791	3.503489055	-21.5306625	4.72E-75	3.06E-72	160.2784551
IL10RA	-1.375163823	3.12541202	-21.52051357	5.31E-75	3.32E-72	160.1602037
PTPN7	-1.057800888	1.95406179	-21.49312125	7.32E-75	4.27E-72	159.8410619
SLC7A7	-1.168362609	3.594397771	-21.37938041	2.77E-74	1.57E-71	158.51626
MAFB	-1.425524515	4.643849865	-21.35655919	3.62E-74	1.98E-71	158.2505224
LCP1	-1.289750964	4.148907492	-21.33446335	4.69E-74	2.49E-71	157.9932555
CTSC	-1.538440764	4.992789161	-21.28481929	8.39E-74	4.32E-71	157.4153268
FCGR2B	-2.017443012	2.635821275	-21.25202294	1.23E-73	6.16E-71	157.033598
VSIG4	-1.813421856	5.869502423	-21.13166166	5.03E-73	2.45E-70	155.6331526
SLA	-1.479253401	3.640543439	-21.10259138	7.07E-73	3.35E-70	155.2950273
ICAM1	-1.859598732	3.806964068	-21.01416111	1.99E-72	9.16E-70	154.266761
CTSS	-1.550855983	4.819088761	-20.98553584	2.78E-72	1.25E-69	153.9340034
LCP2	-1.277292851	3.912751594	-20.97797864	3.03E-72	1.33E-69	153.8461619
MYOF	-1.266309441	3.225249993	-20.93919881	4.77E-72	2.02E-69	153.3954567
RAC2	-1.308507359	3.354680797	-20.9377059	4.85E-72	2.02E-69	153.3781077
LHFPL2	-1.09047262	4.099482665	-20.90244633	7.33E-72	2.98E-69	152.9683976
HCK	-1.244992086	3.817766373	-20.89311238	8.17E-72	3.25E-69	152.8599514
FPR3	-1.47765463	2.643541514	-20.852671	1.31E-71	5.10E-69	152.3901475
C5AR1	-1.62994303	3.456777834	-20.84480842	1.44E-71	5.35E-69	152.2988204
SLAMF8	-1.457075642	2.487977527	-20.844806	1.44E-71	5.35E-69	152.2987924
CCR1	-1.316816501	3.204592452	-20.81360636	2.07E-71	7.47E-69	151.9364348
NCF2	-1.241800095	3.225858822	-20.81265325	2.09E-71	7.47E-69	151.9253663
FPR1	-1.765199032	4.068884983	-20.78514634	2.88E-71	9.97E-69	151.6059501

MS4A6A	-1.649678682	5.53186423	-20.78457274	2.90E-71	9.97E-69	151.5992899
B4GALT1	-1.166998946	3.381758011	-20.77278567	3.33E-71	1.12E-68	151.4624313
C1QC	-1.56560843	8.201034735	-20.56793055	3.63E-70	1.19E-67	149.0853722
PLEK	-1.271435087	3.442989601	-20.56677967	3.68E-70	1.19E-67	149.0720261
MYO1G	-1.363015721	2.04542883	-20.54076948	4.99E-70	1.59E-67	148.7704254
RBM47	-1.277809886	2.317906615	-20.49056864	8.95E-70	2.80E-67	148.1884613
HK3	-1.616883916	2.703312013	-20.47566939	1.06E-69	3.27E-67	148.0157743
LAPTM5	-1.447473167	7.251601332	-20.42194136	1.99E-69	5.99E-67	147.3931867
SASH3	-1.220561513	3.752728082	-20.42077927	2.02E-69	5.99E-67	147.3797231
CYTIP	-1.095845035	2.01865575	-20.39755006	2.64E-69	7.72E-67	147.1106182
C1R	-1.907967223	6.982852801	-20.35804972	4.19E-69	1.20E-66	146.6531115
TLR2	-1.298429126	3.090258351	-20.32421923	6.21E-69	1.75E-66	146.2613714
CD68	-1.522374724	4.836676113	-20.27985688	1.04E-68	2.89E-66	145.747815
DOCK2	-1.104137001	2.703049241	-20.26641922	1.22E-68	3.33E-66	145.5922863
MAP3K8	-1.210797281	2.564240126	-20.22664109	1.93E-68	5.21E-66	145.1319764
SYK	-1.115577695	3.110015418	-20.2046715	2.50E-68	6.62E-66	144.8778012
SLC16A3	-1.773465568	4.787850314	-20.18772137	3.04E-68	7.95E-66	144.6817254
CD300C	-1.002446833	1.925098166	-20.15408474	4.49E-68	1.16E-65	144.2926946
F13A1	-2.641466247	3.623659951	-20.0786744	1.08E-67	2.74E-65	143.4208704
CTSL	-1.230794229	6.806111694	-20.04215355	1.65E-67	4.13E-65	142.9988265
HAVCR2	-1.265165824	4.043319048	-20.01434617	2.28E-67	5.62E-65	142.6775563
CLEC7A	-1.215441999	2.9900954	-19.99156843	2.97E-67	7.22E-65	142.4144465
GNA15	-1.150862291	2.805148565	-19.95207696	4.70E-67	1.13E-64	141.9583839
MYO1F	-1.126300836	3.898631115	-19.90141886	8.46E-67	2.00E-64	141.3735721
ITGAM	-1.212505113	3.172417282	-19.89152553	9.48E-67	2.22E-64	141.2593882
MS4A4A	-1.552952127	3.912743965	-19.8100036	2.44E-66	5.63E-64	140.3188508
PIK3AP1	-1.194837046	2.833062871	-19.80570166	2.57E-66	5.84E-64	140.2692359
C1S	-1.765838457	5.7502475	-19.7795867	3.47E-66	7.70E-64	139.9680854
PIK3R5	-1.13492606	2.528633889	-19.71393055	7.43E-66	1.61E-63	139.2112489
C1QA	-1.600030063	8.137405935	-19.68611333	1.03E-65	2.19E-63	138.8907197
CD300A	-1.236304141	3.966417466	-19.67888885	1.12E-65	2.35E-63	138.8074868
ADAP2	-1.094575051	3.816329553	-19.65080612	1.54E-65	3.22E-63	138.4839966
CYBB	-1.425830179	4.323971022	-19.63803146	1.79E-65	3.69E-63	138.3368691
C1QB	-1.518705056	8.291873551	-19.52474433	6.64E-65	1.35E-62	137.0328558
CACNA2D4	-1.144638655	1.994231649	-19.51860429	7.13E-65	1.44E-62	136.9622177
PTPRC	-1.263925063	2.94822161	-19.4095137	2.52E-64	5.01E-62	135.7078527
LYZ	-1.864416132	4.313277823	-19.40390958	2.69E-64	5.28E-62	135.643449
DSE	-1.04224984	2.63296922	-19.36108868	4.40E-64	8.57E-62	135.1514558
CD53	-1.322566766	5.369999292	-19.33823715	5.73E-64	1.10E-61	134.8889845
SOCS3	-2.311351166	5.082731092	-19.29460875	9.49E-64	1.81E-61	134.3880331
SCIMP	-1.01398566	2.135012397	-19.28276918	1.09E-63	2.05E-61	134.2521253
CYTH4	-1.282691562	3.615118043	-19.28021838	1.12E-63	2.09E-61	134.2228465
THBD	-1.433662624	2.464102822	-19.25299476	1.53E-63	2.83E-61	133.9104115
GPR65	-1.131633461	2.217951621	-19.23077077	1.98E-63	3.62E-61	133.6554182

DPYD	-1.595039509	3.34714628	-19.21906252	2.27E-63	4.10E-61	133.5211029
MS4A7	-1.362169919	4.219122342	-19.20843783	2.56E-63	4.58E-61	133.3992319
CEBPB	-1.31884706	5.326252122	-19.15877369	4.54E-63	8.04E-61	132.8297307
TREM1	-2.272521808	2.87860928	-19.11603619	7.44E-63	1.29E-60	132.3398896
ST14	-1.354368419	2.794003646	-19.11533267	7.50E-63	1.29E-60	132.3318279
ALOX5	-1.370855441	4.058594842	-19.11051003	7.93E-63	1.35E-60	132.2765666
PARVG	-1.205789282	3.799179686	-19.08218943	1.10E-62	1.85E-60	131.9521048
FERMT3	-1.194668157	4.403579976	-19.04577694	1.67E-62	2.79E-60	131.5350781
FCGR2C	-1.769018322	2.665647194	-18.93136945	6.23E-62	1.03E-59	130.2258427
TBXAS1	-1.066540094	3.936446041	-18.91107948	7.87E-62	1.29E-59	129.9938221
LILRB1	-1.158238306	2.645566936	-18.85532978	1.49E-61	2.42E-59	129.35658
CSTA	-1.449094239	2.369014066	-18.83843853	1.81E-61	2.91E-59	129.1635843
TLR1	-1.089629421	2.511300431	-18.83643079	1.85E-61	2.95E-59	129.1406468
GLIPR1	-1.282100058	3.55198582	-18.75936428	4.49E-61	7.03E-59	128.2605921
CDCP1	-1.127078278	1.61102018	-18.71768479	7.25E-61	1.09E-58	127.7849617
S100A9	-2.379884332	5.863401744	-18.61043791	2.48E-60	3.67E-58	126.5621728
NAMPT	-1.897076405	5.935637845	-18.60984616	2.49E-60	3.67E-58	126.5554303
CLCF1	-1.531377703	2.605421108	-18.60566074	2.62E-60	3.82E-58	126.5077417
C1RL	-1.401576578	3.889572081	-18.58343712	3.38E-60	4.89E-58	126.2545669
NFAM1	-1.039738975	2.278993311	-18.55869886	4.48E-60	6.43E-58	125.9728255
HMOX1	-1.72982549	5.978370772	-18.51409498	7.47E-60	1.05E-57	125.465052
DAB2	-1.104757845	3.761388101	-18.48535025	1.04E-59	1.45E-57	125.1379685
TGFBR2	-1.086452266	3.909148161	-18.46800068	1.27E-59	1.76E-57	124.9406062
TYROBP	-1.3139538	7.795226032	-18.4360604	1.82E-59	2.49E-57	124.5773777
CTSD	-1.079227047	8.549154015	-18.40736247	2.53E-59	3.44E-57	124.2511467
C2	-1.484790036	3.72806843	-18.39377843	2.96E-59	3.98E-57	124.0967682
CHI3L1	-3.456755006	9.282550495	-18.35937026	4.38E-59	5.85E-57	123.7058502
MVP	-1.113200432	5.400128091	-18.34347921	5.25E-59	6.97E-57	123.5253674
COL8A1	-2.186778765	2.525939345	-18.30691305	7.97E-59	1.03E-56	123.1102084
GPR84	-1.148435395	1.696784014	-18.3015787	8.47E-59	1.09E-56	123.0496607
GCNT1	-1.219843318	1.567965307	-18.29510575	9.12E-59	1.17E-56	122.9761949
DENND2D	-1.135686159	2.49692995	-18.27214614	1.19E-58	1.50E-56	122.7156618
CSF1R	-1.276650862	5.465809398	-18.27055667	1.21E-58	1.52E-56	122.6976283
VAV1	-1.055914174	2.912427596	-18.21076422	2.39E-58	2.99E-56	122.0195231
TNFRSF1A	-1.054742503	6.08496989	-18.12395773	6.41E-58	7.91E-56	121.0360244
ALOX5AP	-1.744213387	5.542633559	-18.10781746	7.71E-58	9.44E-56	120.8532872
STAB1	-1.602608834	5.587019037	-18.06863844	1.20E-57	1.46E-55	120.4098796
PLBD1	-1.120540254	2.433563516	-18.06548758	1.25E-57	1.51E-55	120.3742303
LILRA6	-1.019627488	1.207037712	-18.05977786	1.33E-57	1.60E-55	120.3096338
CFI	-1.611727857	4.389417359	-17.97992475	3.30E-57	3.91E-55	119.4067646
ITGA5	-1.399846584	4.42798765	-17.9774362	3.40E-57	3.99E-55	119.3786439
PLXDC2	-1.163287435	3.434602751	-17.94265092	5.04E-57	5.85E-55	118.9856746
IL1R1	-1.359814995	2.327955254	-17.91838476	6.64E-57	7.65E-55	118.7116564
CSF1	-1.086490998	4.928006295	-17.90898215	7.39E-57	8.46E-55	118.6055062

TYMP	-1.701813483	5.461907351	-17.90665737	7.59E-57	8.63E-55	118.579263
C3AR1	-1.18543103	4.254450542	-17.85927246	1.30E-56	1.46E-54	118.0445539
SAT1	-1.106504847	8.29573798	-17.82539469	1.91E-56	2.10E-54	117.662493
NCF4	-1.124901616	3.623009955	-17.81536966	2.14E-56	2.34E-54	117.5494713
SLC2A5	-1.454304006	4.640891383	-17.80567868	2.38E-56	2.59E-54	117.4402316
LILRB2	-1.146988377	2.033364569	-17.7782791	3.25E-56	3.52E-54	117.1314611
LILRB3	-1.197707784	1.65420986	-17.75110602	4.43E-56	4.73E-54	116.8253687
SERPINE1	-2.19710264	5.077548004	-17.70637889	7.34E-56	7.75E-54	116.3218126
HLA-DMB	-1.298442894	5.123133922	-17.69915856	7.97E-56	8.36E-54	116.2405554
SOD2	-1.908009105	7.550805196	-17.6894889	8.89E-56	9.27E-54	116.1317477
PDCD1LG2	-1.001754549	1.798339188	-17.629612	1.75E-55	1.81E-53	115.4583459
PTAFR	-1.092198777	2.968308409	-17.61904269	1.97E-55	2.03E-53	115.3395439
CXCL16	-1.085995018	5.509393095	-17.61336207	2.10E-55	2.15E-53	115.2757003
CD86	-1.103985976	3.161195234	-17.57759968	3.15E-55	3.21E-53	114.873903
LILRB4	-1.238914245	4.403579823	-17.56412757	3.67E-55	3.71E-53	114.7226
CD44	-1.674927288	6.495268841	-17.5472196	4.44E-55	4.47E-53	114.5327554
TNFAIP3	-1.242175349	3.061978051	-17.4595084	1.19E-54	1.16E-52	113.548749
JAK3	-1.122138221	2.147373914	-17.27811348	9.20E-54	8.76E-52	111.518219
CD74	-1.420897648	10.1345674	-17.20191962	2.16E-53	2.04E-51	110.6671581
CASP4	-1.165066301	3.747806751	-17.1835686	2.66E-53	2.49E-51	110.4623501
RNASE2	-1.593540507	3.165283432	-17.16292813	3.35E-53	3.12E-51	110.2320686
IL7R	-1.085347842	1.277680137	-17.15316841	3.74E-53	3.45E-51	110.1232103
COL1A1	-2.49233687	5.237837874	-17.14196805	4.24E-53	3.89E-51	109.9983064
HLA-DMA	-1.246270415	5.643844931	-17.13658681	4.51E-53	4.11E-51	109.9383046
CCL2	-2.202958349	5.807358555	-17.13564722	4.55E-53	4.13E-51	109.9278286
SRPX2	-1.715513086	3.512375739	-17.09278672	7.36E-53	6.58E-51	109.4501384
STEAP3	-1.544322722	4.212542943	-17.05458067	1.13E-52	1.00E-50	109.0246295
ABCC3	-2.036383537	3.611523125	-16.94438132	3.87E-52	3.38E-50	107.7989568
LTBP2	-1.254234383	2.284044645	-16.94000172	4.07E-52	3.53E-50	107.7502964
RHBDF2	-1.179523144	3.991364043	-16.88222295	7.76E-52	6.66E-50	107.1087025
DOK2	-1.193002716	2.065232283	-16.84912034	1.12E-51	9.59E-50	106.7414315
HLA-DRA	-1.479861586	8.239894167	-16.82688899	1.44E-51	1.22E-49	106.4949044
BCL3	-1.321669315	3.887130328	-16.81270002	1.68E-51	1.42E-49	106.3376147
FPR2	-1.125428565	0.999858907	-16.80490068	1.84E-51	1.54E-49	106.2511743
SP100	-1.128788703	3.981859075	-16.79163516	2.13E-51	1.77E-49	106.1041815
PTGER4	-1.106606155	1.840072104	-16.77998089	2.43E-51	1.99E-49	105.9750732
AQP9	-1.450112502	1.544179795	-16.70879167	5.36E-51	4.39E-49	105.1870518
SPP1	-2.150444772	9.984361027	-16.70369774	5.67E-51	4.62E-49	105.1307066
SCIN	-1.501311425	3.055388192	-16.69766254	6.06E-51	4.92E-49	105.0639569
IL2RA	-1.418131754	1.29953654	-16.68606859	6.90E-51	5.52E-49	104.9357494
RNASET2	-1.009512616	6.023875584	-16.6845645	7.02E-51	5.59E-49	104.9191191
SRGN	-1.499998122	6.260438296	-16.66523561	8.70E-51	6.89E-49	104.7054481
HLA-DOA	-1.308164698	3.527212834	-16.60017631	1.79E-50	1.39E-48	103.9868476
CD84	-1.064776576	2.330967942	-16.51052555	4.84E-50	3.74E-48	102.9981514

SPI1	-1.228339553	5.559471163	-16.50613002	5.09E-50	3.91E-48	102.9497222
SERPINB1	-1.050555957	4.034102937	-16.48323788	6.55E-50	4.99E-48	102.6975699
SPOCD1	-2.222141945	4.767061416	-16.47364604	7.29E-50	5.53E-48	102.5919527
CD33	-1.02644482	2.699030629	-16.45425728	9.03E-50	6.82E-48	102.3785235
S100A8	-2.173845364	4.757552565	-16.43654419	1.10E-49	8.26E-48	102.1836141
NFKBIZ	-1.424229407	2.669141273	-16.41165291	1.45E-49	1.08E-47	101.9098385
APOBEC3C	-1.050605075	3.674028962	-16.40727146	1.52E-49	1.13E-47	101.8616622
VAMP8	-1.273098445	5.669376135	-16.35342074	2.75E-49	2.04E-47	101.2699044
OSCAR	-1.16100918	2.768535159	-16.32364983	3.83E-49	2.82E-47	100.9430429
BACE2	-1.097683388	3.381458883	-16.29979152	4.98E-49	3.63E-47	100.6812455
RDH10	-1.513899851	4.111266629	-16.2705892	6.87E-49	4.93E-47	100.3609889
ANXA2	-1.596304571	7.173625038	-16.25211891	8.43E-49	6.00E-47	100.1585318
FAM20A	-1.395465266	2.886061412	-16.2251523	1.13E-48	7.95E-47	99.86308873
WAS	-1.038329551	3.921265003	-16.214648	1.27E-48	8.85E-47	99.7480513
CASP1	-1.05661155	4.033309743	-16.17792441	1.91E-48	1.31E-46	99.34608032
SIGLEC10	-1.209982352	3.872216499	-16.15090841	2.57E-48	1.74E-46	99.0505727
CLEC5A	-1.439585037	2.342872851	-16.13679785	3.00E-48	2.02E-46	98.8962975
MAN1A1	-1.101008262	2.594313517	-16.12915181	3.26E-48	2.18E-46	98.81272094
CEBPD	-1.330243322	5.93409812	-16.08594946	5.25E-48	3.48E-46	98.34075437
CSF3R	-1.139695064	3.689253496	-16.08412928	5.35E-48	3.54E-46	98.32087963
AMPD3	-1.046974902	2.842543624	-16.08343007	5.39E-48	3.54E-46	98.31324509
EMB	-1.143205923	2.141003546	-16.07274897	6.06E-48	3.96E-46	98.1966351
OSMR	-1.299665773	3.496722885	-16.01032936	1.20E-47	7.83E-46	97.51573217
TGFBI	-1.738410485	5.938900195	-15.98405528	1.60E-47	1.04E-45	97.2294086
BCL2A1	-1.574245462	2.948369448	-15.98220637	1.64E-47	1.05E-45	97.20926637
COL1A2	-2.091639186	5.711664143	-15.96930528	1.89E-47	1.21E-45	97.0687444
FOSL2	-1.328806922	4.444367799	-15.96874065	1.90E-47	1.21E-45	97.06259526
TNFAIP2	-1.498427946	4.716262206	-15.88303755	4.85E-47	3.02E-45	96.1301616
RUNX1	-1.184810419	2.930729369	-15.86514501	5.90E-47	3.65E-45	95.9357271
SAMSN1	-1.149897368	3.368498493	-15.79546938	1.26E-46	7.65E-45	95.17935249
NRP1	-1.202233576	4.162706592	-15.7582626	1.89E-46	1.14E-44	94.77595792
IL4I1	-1.260759322	2.484755391	-15.72458189	2.73E-46	1.64E-44	94.41110136
OSM	-1.543810663	2.300380109	-15.70235923	3.48E-46	2.07E-44	94.17052907
LIF	-1.845494782	2.574703347	-15.66799629	5.06E-46	2.99E-44	93.79878588
PTX3	-2.037100381	3.574799158	-15.66771089	5.08E-46	2.99E-44	93.7956997
PLAU	-1.618018997	4.191768541	-15.62424472	8.14E-46	4.75E-44	93.32592445
FCGBP	-1.789412547	4.679013748	-15.56911138	1.48E-45	8.57E-44	92.73077302
COL3A1	-2.339241335	5.38830929	-15.56405607	1.57E-45	9.02E-44	92.67624271
COL6A3	-2.059157579	2.593410424	-15.56068477	1.62E-45	9.33E-44	92.63988122
BATF	-1.138540605	2.050307651	-15.53927365	2.05E-45	1.17E-43	92.40902047
CMKLR1	-1.002214195	2.530797761	-15.53039944	2.26E-45	1.28E-43	92.31337234
FGL2	-1.049104399	3.137001729	-15.52290604	2.45E-45	1.39E-43	92.23262342
CD93	-1.301153214	3.793115089	-15.505503	2.95E-45	1.66E-43	92.04514662
IL1RN	-1.363561812	1.820159041	-15.50466423	2.98E-45	1.67E-43	92.036113

CP	-1.770718644	3.943421585	-15.49883809	3.18E-45	1.77E-43	91.97336963
FAM20C	-1.313726358	6.197309291	-15.48681982	3.62E-45	2.00E-43	91.84397065
OLFML2B	-1.25910567	3.787686653	-15.45459961	5.13E-45	2.82E-43	91.49725411
SLC37A2	-1.042507012	2.292561181	-15.45058278	5.35E-45	2.93E-43	91.45404946
MMP19	-1.529896514	2.444466087	-15.41628932	7.76E-45	4.21E-43	91.08537234
ZDHHC22	1.711632288	3.19393949	15.41127269	8.19E-45	4.40E-43	91.03146735
HSPA7	-1.614244477	3.244619416	-15.37703492	1.19E-44	6.30E-43	90.663759
PRSS23	-1.297772853	4.351489867	-15.37187098	1.25E-44	6.64E-43	90.60832724
GBP2	-1.333142452	4.718274676	-15.35414197	1.52E-44	7.99E-43	90.4180733
ACSL1	-1.01665832	4.209428322	-15.28613428	3.16E-44	1.62E-42	89.68908107
THBS1	-1.884343845	3.271387246	-15.26666225	3.90E-44	1.99E-42	89.48059436
CYBA	-1.100620089	7.061872165	-15.26643878	3.91E-44	1.99E-42	89.47820224
PYGL	-1.068486927	4.624306647	-15.26279746	4.07E-44	2.06E-42	89.43922684
FN1	-1.56248015	7.729203691	-15.24482549	4.93E-44	2.48E-42	89.24691594
CLIC1	-1.180564911	7.141591887	-15.23954836	5.22E-44	2.62E-42	89.19046481
FBP1	-1.12870798	2.794907046	-15.21878767	6.53E-44	3.25E-42	88.96845805
CAPG	-1.240677992	6.893585834	-15.19169372	8.74E-44	4.30E-42	88.67891056
FTL	-1.219804127	11.8851843	-15.18993595	8.91E-44	4.37E-42	88.66013296
S100A11	-1.476402023	7.877442375	-15.16793813	1.13E-43	5.50E-42	88.42521232
IRF1	-1.001985414	3.877033686	-15.14413259	1.46E-43	7.07E-42	88.17114325
FSD1	1.087982369	4.568608767	15.1346675	1.61E-43	7.80E-42	88.07017076
TCIRG1	-1.168864786	4.9114802	-15.12351423	1.82E-43	8.77E-42	87.95122215
MAN1C1	-1.257830312	4.738670166	-15.11478087	2.00E-43	9.58E-42	87.85810678
PLIN2	-1.282229032	4.607389097	-15.10391095	2.24E-43	1.07E-41	87.7422423
COL5A1	-1.726303596	3.458245417	-15.0917846	2.55E-43	1.22E-41	87.61302585
CIITA	-1.107053725	2.681728928	-15.07820192	2.95E-43	1.40E-41	87.46834171
SLC39A8	-1.030880079	3.452785284	-15.05088143	3.96E-43	1.87E-41	87.17748434
GPNMB	-1.623812278	5.356058701	-15.04100052	4.40E-43	2.04E-41	87.07234474
NPC2	-1.144157288	7.327267309	-15.02249516	5.37E-43	2.45E-41	86.87551239
IBSP	-1.898288298	2.666975977	-14.99418178	7.26E-43	3.30E-41	86.57455234
RAB42	-1.021960334	2.631586772	-14.95937198	1.05E-42	4.78E-41	86.20486329
TGM2	-1.094894429	3.92613384	-14.86967613	2.74E-42	1.22E-40	85.25393903
MSN	-1.160493162	6.682502597	-14.85745369	3.13E-42	1.38E-40	85.12454849
HLA-DPA1	-1.344830454	7.061209782	-14.84090654	3.73E-42	1.64E-40	84.94944742
CD37	-1.081077262	4.4308006	-14.82679416	4.33E-42	1.90E-40	84.80017677
FCER1G	-1.432152206	6.625318761	-14.81241766	5.05E-42	2.19E-40	84.64817468
PRF1	-1.204738929	1.985657805	-14.80354521	5.55E-42	2.40E-40	84.55439828
A2M	-1.090493952	7.791102563	-14.80007381	5.76E-42	2.48E-40	84.51771419
BIRC3	-1.162142622	1.718180486	-14.79411515	6.13E-42	2.64E-40	84.45475455
TMBIM1	-1.026806995	5.821427466	-14.75212226	9.58E-42	4.08E-40	84.01136376
PROS1	-1.059263404	4.190469033	-14.69536504	1.75E-41	7.32E-40	83.41294578
TPP1	-1.017799808	6.532757045	-14.68249783	2.01E-41	8.35E-40	83.27741997
MNDA	-1.080492156	3.29512105	-14.62135568	3.83E-41	1.58E-39	82.6341388
IER3	-1.359391109	3.918969741	-14.60181294	4.71E-41	1.93E-39	82.42877614

CPVL	-1.179887218	5.024114273	-14.52581288	1.05E-40	4.22E-39	81.6312898
BRSK2	1.348597466	3.684402439	14.4739728	1.82E-40	7.21E-39	81.08837945
CKB	1.379344331	9.493825192	14.44757924	2.40E-40	9.46E-39	80.81229817
SHC1	-1.023281542	5.507832587	-14.41827673	3.26E-40	1.28E-38	80.50605351
LOXL2	-1.377910811	3.981911195	-14.40885978	3.60E-40	1.41E-38	80.40769491
KCNIP2	1.435355421	2.903984037	14.39587208	4.13E-40	1.62E-38	80.27208797
SH3TC1	-1.00311799	3.385365682	-14.39093816	4.35E-40	1.69E-38	80.22058638
ANXA1	-1.618165985	6.714312775	-14.34778788	6.85E-40	2.64E-38	79.77051302
SLCO2B1	-1.201475022	5.28371879	-14.34220382	7.26E-40	2.79E-38	79.71231399
MAST1	1.276558235	3.395464357	14.3413793	7.32E-40	2.81E-38	79.70372142
PI3	-2.61075785	2.813660632	-14.33437404	7.88E-40	3.00E-38	79.63072653
COL8A2	-1.138942291	2.466024672	-14.33365596	7.94E-40	3.02E-38	79.62324507
MARCO	-1.837436884	1.964134433	-14.29104661	1.24E-39	4.68E-38	79.1796152
S100A4	-1.489383935	5.425722948	-14.28827911	1.28E-39	4.81E-38	79.15082209
NNMT	-2.279709715	5.530670661	-14.2668275	1.60E-39	6.00E-38	78.92772537
PODXL2	1.134744216	6.002568411	14.26147023	1.69E-39	6.32E-38	78.87203373
GPX8	-1.242005835	2.787321753	-14.20987499	2.90E-39	1.07E-37	78.33616619
HAMP	-1.670099443	3.868672969	-14.18097498	3.92E-39	1.43E-37	78.03640246
CYP1B1	-1.471895914	2.321802955	-14.16441775	4.66E-39	1.70E-37	77.86479074
LY96	-1.284470249	4.56216184	-14.15883653	4.94E-39	1.79E-37	77.80696367
CCL20	-1.498626752	1.390416195	-14.14197558	5.89E-39	2.12E-37	77.63233166
DUSP26	1.439396605	3.415322843	14.1119779	8.05E-39	2.87E-37	77.32187946
HLA-DPB1	-1.400747073	6.208787292	-13.99533995	2.71E-38	9.52E-37	76.11770829
PLTP	-1.031988319	7.57050571	-13.9633131	3.77E-38	1.32E-36	75.78788872
EFEMP1	-1.571408907	6.582152672	-13.95890596	3.95E-38	1.38E-36	75.74253101
ABCA1	-1.192871837	3.982338523	-13.92606068	5.55E-38	1.92E-36	75.40470694
TIMP1	-2.090752353	8.511029959	-13.92516778	5.60E-38	1.93E-36	75.39552852
HSPA6	-1.424131279	3.362770935	-13.92502986	5.61E-38	1.93E-36	75.39411078
SLC15A3	-1.04370312	4.86310641	-13.89636062	7.55E-38	2.59E-36	75.0995594
ENHO	1.529208187	5.626117985	13.89369417	7.76E-38	2.65E-36	75.07217871
GDAP1L1	1.641058564	3.750153981	13.88604579	8.40E-38	2.87E-36	74.99365465
GPR183	-1.08947291	2.78198688	-13.8606443	1.09E-37	3.70E-36	74.73301243
RNASE6	-1.032008042	4.206068778	-13.83580534	1.41E-37	4.76E-36	74.47836433
IL1B	-1.443227392	3.014660253	-13.80515822	1.93E-37	6.48E-36	74.16447479
ANPEP	-1.200331619	2.452651416	-13.78671959	2.34E-37	7.78E-36	73.97578746
PDPN	-1.754357808	5.692525576	-13.77213811	2.72E-37	9.00E-36	73.82665807
WWTR1	-1.159823565	4.84502796	-13.75131555	3.37E-37	1.11E-35	73.61383201
BHLHE40	-1.100059836	4.941905712	-13.75069948	3.39E-37	1.12E-35	73.6075376
RUNDC3A	1.56023933	4.782903354	13.74718379	3.52E-37	1.16E-35	73.57162023
METRNL	-1.006487985	4.887236339	-13.69988883	5.72E-37	1.87E-35	73.08887586
RAC3	1.096665307	4.509444518	13.68694295	6.53E-37	2.13E-35	72.95687794
GNG4	1.313755243	3.914671833	13.62357532	1.25E-36	4.05E-35	72.31165816
MMP14	-1.407223179	6.184006414	-13.59322278	1.71E-36	5.48E-35	72.00312715
ACTN1	-1.112066794	5.863907317	-13.57375253	2.08E-36	6.67E-35	71.8053932

TMEM176B	-1.327129786	6.310342275	-13.54267796	2.86E-36	9.07E-35	71.49010128
PCED1B-AS1	-1.135563938	4.05213565	-13.53002541	3.25E-36	1.03E-34	71.36182785
TAGLN2	-1.147480854	7.784130864	-13.50807986	4.07E-36	1.28E-34	71.1394827
FNDC3B	-1.004679215	3.479564306	-13.50619418	4.15E-36	1.30E-34	71.120386
TMEM145	1.148725409	3.892889946	13.49810185	4.51E-36	1.41E-34	71.03844836
NRP2	-1.091522697	4.373343422	-13.49081857	4.85E-36	1.51E-34	70.96472374
LUM	-1.806599909	3.646954462	-13.44199446	7.98E-36	2.46E-34	70.47102025
RGS1	-1.593432619	5.517310289	-13.39868484	1.24E-35	3.78E-34	70.03383426
CHI3L2	-2.123005636	6.633133786	-13.37924438	1.51E-35	4.56E-34	69.8378261
JUNB	-1.203620033	6.975991491	-13.32411441	2.64E-35	7.90E-34	69.28276621
LINGO1	1.360728816	4.668513265	13.30654989	3.16E-35	9.42E-34	69.1061686
CFD	-1.399596356	3.621512227	-13.30440548	3.23E-35	9.61E-34	69.08461636
LOX	-1.504140691	2.962535309	-13.29509056	3.54E-35	1.05E-33	68.99101818
FZD7	-1.20258905	3.70437878	-13.26680452	4.72E-35	1.39E-33	68.70700025
FOSL1	-1.177602511	2.705554649	-13.26398777	4.85E-35	1.42E-33	68.6787345
CA12	-1.524911375	3.729009525	-13.24609011	5.82E-35	1.70E-33	68.49920569
TNFRSF12A	-1.419481922	5.932877861	-13.24570794	5.84E-35	1.70E-33	68.49537356
RNF165	1.036203968	2.041871425	13.23997842	6.19E-35	1.80E-33	68.43792885
ADAM8	-1.241575329	2.729052543	-13.22515143	7.19E-35	2.08E-33	68.28933135
GAD1	1.412266087	3.203161599	13.20720855	8.61E-35	2.49E-33	68.10962116
C1orf162	-1.074223231	4.040143328	-13.20206949	9.07E-35	2.61E-33	68.05817315
TUBA1C	-1.224806576	5.576603871	-13.15816629	1.41E-34	4.01E-33	67.6190742
HES5	1.580576234	2.357481577	13.14521783	1.61E-34	4.56E-33	67.48971485
ACP5	-1.296771005	2.909311014	-13.12580333	1.95E-34	5.51E-33	67.29588202
ADAM12	-1.182892978	2.585356087	-13.11806867	2.11E-34	5.94E-33	67.21870145
RCOR2	1.406620474	3.404059152	13.08564806	2.93E-34	8.18E-33	66.89544986
MRC2	-1.255034805	5.892644947	-13.0821138	3.03E-34	8.44E-33	66.86023659
RIPPLY2	1.344712559	2.466092	13.07719121	3.19E-34	8.86E-33	66.81119926
FBXO32	-1.152890213	4.73639276	-13.05435058	4.01E-34	1.11E-32	66.58379423
CCL5	-1.187167978	2.98632144	-13.04780443	4.28E-34	1.18E-32	66.51865819
TTC9B	1.704807906	3.306200286	13.04052151	4.60E-34	1.26E-32	66.4462113
CXCR4	-1.037569105	4.998521569	-13.01360537	6.03E-34	1.65E-32	66.17864774
LGALS3	-1.501909865	6.575604138	-13.0028827	6.71E-34	1.83E-32	66.07213891
MXRA5	-1.186191116	2.09719579	-12.99805582	7.04E-34	1.91E-32	66.02420831
CASKIN1	1.184269771	2.956618215	12.99742961	7.08E-34	1.92E-32	66.01799077
SBK1	1.109878626	3.330630201	12.97631405	8.75E-34	2.36E-32	65.80843105
SECTM1	-1.033761488	2.930234451	-12.97591888	8.78E-34	2.36E-32	65.8045109
G0S2	-1.596111885	3.509351047	-12.97553177	8.82E-34	2.37E-32	65.80067085
APOL1	-1.069061239	3.505593035	-12.96261847	1.00E-33	2.69E-32	65.67260678
COL6A2	-1.736604081	6.099110944	-12.94896134	1.15E-33	3.07E-32	65.53723978
DUSP1	-1.369400478	6.167621	-12.94769909	1.16E-33	3.11E-32	65.52473232
ACTA2	-1.147854167	5.977423997	-12.89102911	2.05E-33	5.40E-32	64.96387063

SLC39A14	-1.068283798	4.524319115	-12.86970848	2.53E-33	6.60E-32	64.75320109
LTF	-2.87234806	5.050952059	-12.86708222	2.60E-33	6.77E-32	64.72726386
ADAMTS1	-1.106410632	3.312177142	-12.86343175	2.70E-33	6.98E-32	64.69121615
RARRES1	-1.274835923	2.118966189	-12.85700705	2.87E-33	7.42E-32	64.62778684
TAGLN	-1.365513193	6.883479543	-12.84722396	3.17E-33	8.15E-32	64.53123361
VASN	-1.158431343	3.639601716	-12.83962868	3.42E-33	8.78E-32	64.45630004
SERPING1	-1.327692945	6.467831373	-12.7932634	5.42E-33	1.38E-31	63.99938638
TNC	-1.495454791	6.354866332	-12.78987201	5.60E-33	1.43E-31	63.96600031
IL6	-1.412446923	1.798994535	-12.77599061	6.43E-33	1.63E-31	63.82939709
UPP1	-1.089315853	5.07490341	-12.73747375	9.41E-33	2.37E-31	63.45078361
COL5A2	-1.422068784	4.366431771	-12.70866596	1.25E-32	3.14E-31	63.16801438
C3	-1.491182723	8.590420339	-12.68534675	1.58E-32	3.92E-31	62.93937502
SEZ6L	1.585496136	3.796963555	12.68211482	1.63E-32	4.04E-31	62.90770483
C15orf48	-1.177793994	1.818851624	-12.669658	1.84E-32	4.56E-31	62.78567965
MMD2	1.261317645	2.200684993	12.66429108	1.94E-32	4.80E-31	62.73312622
MAP2	1.158872261	5.729937787	12.64826849	2.27E-32	5.61E-31	62.57630416
HES6	1.535536043	6.766870028	12.64801791	2.28E-32	5.61E-31	62.57385248
ZFP36	-1.235245927	6.169741664	-12.64338569	2.38E-32	5.87E-31	62.52853505
FXYD6	1.32750064	7.526731839	12.62598905	2.83E-32	6.93E-31	62.35842363
SCG3	1.500403853	5.167467618	12.62307356	2.91E-32	7.12E-31	62.32992742
BTBD17	1.580264362	3.489470438	12.61946075	3.02E-32	7.35E-31	62.29462051
RCAN1	-1.141527661	6.227001424	-12.61571404	3.13E-32	7.62E-31	62.25801085
ADAMTS14	-1.03794186	1.97154591	-12.61154095	3.26E-32	7.92E-31	62.21724212
CDK5R1	1.151472205	3.568031313	12.58448734	4.26E-32	1.03E-30	61.95312351
IGFBP7	-1.023349113	9.031835411	-12.57713454	4.58E-32	1.10E-30	61.88139377
CLEC2B	-1.024018016	2.821184567	-12.50162244	9.60E-32	2.28E-30	61.14608391
SHISA7	1.155137781	1.791223051	12.44861416	1.61E-31	3.77E-30	60.63138011
CNN2	-1.110289506	4.062363559	-12.43761619	1.80E-31	4.19E-30	60.52474429
ITGA3	-1.217694589	4.41592639	-12.43079422	1.92E-31	4.47E-30	60.45862522
CAV1	-1.217708899	4.718465352	-12.42776737	1.98E-31	4.60E-30	60.42929524
NKAIN4	1.709552631	5.477283255	12.4108304	2.33E-31	5.40E-30	60.26525118
STX1B	1.024647503	3.374172027	12.40689059	2.43E-31	5.60E-30	60.22710993
STC1	-1.114737846	2.173303422	-12.39905667	2.62E-31	6.02E-30	60.15128998
GBP1	-1.197608059	4.517188285	-12.36166772	3.77E-31	8.58E-30	59.78979555
VIM	-1.069477096	10.20085894	-12.34564219	4.40E-31	9.98E-30	59.63504135
EMILIN1	-1.215660595	5.393284289	-12.31757548	5.79E-31	1.30E-29	59.36428229
FCGR1A	-1.169774845	5.230136903	-12.3131179	6.04E-31	1.36E-29	59.32131216
CNTFR	1.278768355	4.519382441	12.30354843	6.63E-31	1.48E-29	59.22909434
CELF3	1.479162597	3.074913361	12.26322412	9.81E-31	2.17E-29	58.84094974
MMP7	-1.486619817	1.697577919	-12.25053044	1.11E-30	2.44E-29	58.71891587
EMP1	-1.417805884	6.018526832	-12.23223153	1.32E-30	2.91E-29	58.54312148
EMP3	-1.346166062	6.551446737	-12.21357685	1.59E-30	3.47E-29	58.36406394
MYCN	1.202048423	2.655855216	12.21268847	1.60E-30	3.49E-29	58.35554064
SAA2	-1.996190275	1.957869869	-12.18514402	2.09E-30	4.54E-29	58.0914514

SERPINH1	-1.097272528	6.144651024	-12.18390368	2.11E-30	4.59E-29	58.07956731
SCRT1	1.272684112	1.664312116	12.17920986	2.21E-30	4.79E-29	58.03460086
AIF1	-1.152803579	6.320004645	-12.16976041	2.42E-30	5.24E-29	57.94410604
C6orf141	-1.440060352	2.154248833	-12.15017977	2.93E-30	6.31E-29	57.75671612
CKMT1B	1.269892514	1.711206531	12.09748227	4.86E-30	1.04E-28	57.25325656
MYL9	-1.107170581	5.460099672	-12.07794839	5.87E-30	1.25E-28	57.06695593
SERPINA3	-1.537976473	6.409001456	-12.07613768	5.97E-30	1.27E-28	57.04969548
SOX8	1.608041914	5.918484479	12.07262468	6.17E-30	1.31E-28	57.01621232
CXCL5	-1.269268359	1.310258165	-12.06831148	6.44E-30	1.36E-28	56.97511003
LYVE1	-1.193300919	1.951339725	-12.05661238	7.20E-30	1.53E-28	56.86366724
MMP11	-1.014504775	2.277149031	-12.03028147	9.27E-30	1.95E-28	56.61307538
CAMSAP3	1.147624313	1.87552655	12.0038821	1.19E-29	2.50E-28	56.36215242
CXCL3	-1.232931384	1.694631014	-11.99938732	1.25E-29	2.60E-28	56.3194621
MEDAG	-1.014797834	1.184400978	-11.98667881	1.41E-29	2.93E-28	56.19881024
GEM	-1.001992085	4.086959177	-11.97728826	1.54E-29	3.20E-28	56.10970645
LAMB1	-1.116468038	4.93119314	-11.97326936	1.60E-29	3.31E-28	56.07158488
PLP2	-1.220651892	5.540808695	-11.97218127	1.62E-29	3.34E-28	56.06126509
ATP1A3	1.732003073	4.474313589	11.96802052	1.68E-29	3.46E-28	56.02180804
CDKN1A	-1.042030118	5.983027939	-11.96293068	1.77E-29	3.62E-28	55.97355117
TSPAN7	1.133106772	6.752445641	11.96161797	1.79E-29	3.67E-28	55.96110733
BEX1	1.454226368	7.009697017	11.95897215	1.84E-29	3.76E-28	55.93602861
CKMT1A	1.218099115	1.462303542	11.94814075	2.04E-29	4.15E-28	55.83339593
OLIG1	1.765646981	7.293247161	11.90702631	3.01E-29	6.09E-28	55.44431445
CBLN1	1.061341991	1.231124421	11.85780833	4.81E-29	9.57E-28	54.97958613
INA	1.643233494	2.477731407	11.83270605	6.11E-29	1.21E-27	54.74300273
SHD	1.867747677	4.052845706	11.82978468	6.28E-29	1.25E-27	54.71548883
PLA2G2A	-2.354941889	3.348692455	-11.82653029	6.48E-29	1.28E-27	54.68484316
SDC2	-1.004139853	4.971310497	-11.82558404	6.54E-29	1.29E-27	54.67593357
CXCL1	-1.240653496	1.736364523	-11.7733232	1.07E-28	2.10E-27	54.18451867
CSPG5	1.390299063	5.78639567	11.76387465	1.17E-28	2.29E-27	54.09581125
FSTL1	-1.011254762	5.547893868	-11.7624722	1.19E-28	2.31E-27	54.08264801
KCNE4	-1.105840883	3.216580065	-11.74591621	1.39E-28	2.69E-27	53.92732625
NDRG1	-1.251061611	6.519637333	-11.74473013	1.41E-28	2.71E-27	53.91620389
ASCL1	1.413846523	4.052535043	11.71028978	1.95E-28	3.73E-27	53.59353714
TREM2	-1.015780884	6.363424488	-11.69428891	2.27E-28	4.33E-27	53.44382063
LGALS1	-1.014518216	9.711347405	-11.69256329	2.30E-28	4.40E-27	53.42768174
CELF5	1.358694924	2.655037495	11.66164282	3.08E-28	5.84E-27	53.13874028
SLIT1	1.328327142	3.294191739	11.65500202	3.28E-28	6.21E-27	53.07674434
FBLL1	1.204947256	3.577872287	11.65487265	3.29E-28	6.22E-27	53.0755368
SNAP91	1.401042609	2.527655992	11.64537081	3.59E-28	6.78E-27	52.98686891
CXCL6	-1.110782356	0.848901845	-11.64429978	3.63E-28	6.83E-27	52.97687716
NECAB2	1.318026507	2.96891841	11.64426371	3.63E-28	6.83E-27	52.97654064
OLR1	-1.09357509	3.97526365	-11.62664216	4.29E-28	8.01E-27	52.81222741
PODNL1	-1.090052457	2.09726502	-11.61318849	4.86E-28	9.06E-27	52.68687907

GADD45B	-1.036036448	6.077112103	-11.6069594	5.16E-28	9.58E-27	52.62887219
UBD	-1.242986726	2.136547036	-11.56868672	7.38E-28	1.36E-26	52.27288118
ACTL6B	1.551654711	2.693023771	11.53869716	9.78E-28	1.79E-26	51.99443444
ODF3B	-1.098345977	3.45172177	-11.50220805	1.38E-27	2.50E-26	51.65623499
PCSK2	1.345036559	1.832099684	11.48607483	1.60E-27	2.90E-26	51.50691288
CPNE5	1.115165373	3.589337074	11.45541031	2.13E-27	3.82E-26	51.22344919
DLL3	1.902210952	4.540183753	11.42030391	2.95E-27	5.25E-26	50.89949614
HLA-DRB1	-1.438064397	6.56761531	-11.40850636	3.29E-27	5.85E-26	50.79076869
SAA1	-2.428101616	3.418234323	-11.40297437	3.47E-27	6.14E-26	50.73980916
FOLR2	-1.233152367	4.682075883	-11.39164114	3.85E-27	6.81E-26	50.63545739
TAGLN3	1.503174022	5.060734948	11.39034091	3.90E-27	6.89E-26	50.62348948
ALDOC	1.279542121	7.258178657	11.384901	4.10E-27	7.24E-26	50.57342727
NDRG2	1.207218213	7.628875758	11.38379598	4.14E-27	7.30E-26	50.56325988
DDX25	1.01310413	2.208599175	11.37962563	4.31E-27	7.58E-26	50.52489343
SMIM18	1.080131911	1.458356778	11.34226403	6.09E-27	1.06E-25	50.18156175
DLL1	1.2128637	3.541529286	11.33822658	6.32E-27	1.10E-25	50.14450181
PHYHIPL	1.195209929	4.926804968	11.32002532	7.48E-27	1.30E-25	49.97753305
AHNAK2	-1.030658528	2.052961608	-11.31440519	7.88E-27	1.37E-25	49.9260106
CCL18	-1.445125717	1.295293839	-11.29173174	9.72E-27	1.68E-25	49.71831377
MYBPH	-1.313473035	1.815640059	-11.28899086	9.97E-27	1.72E-25	49.69322394
PIANP	1.047917942	4.131810296	11.26926973	1.20E-26	2.04E-25	49.51280967
RETN	-1.209261708	1.272011043	-11.2542362	1.37E-26	2.33E-25	49.37541114
SPTBN2	1.118961014	3.927552973	11.23725516	1.61E-26	2.72E-25	49.22035121
NEU4	1.439695758	3.467122163	11.20917064	2.08E-26	3.51E-25	48.96422304
RAB3A	1.203429077	3.902827278	11.20189162	2.23E-26	3.74E-25	48.89790463
FBLIM1	-1.147644595	3.753387443	-11.17432098	2.87E-26	4.78E-25	48.64695681
RPRM	1.342709796	2.808785719	11.1695479	3.00E-26	4.98E-25	48.60355175
NSG2	1.745396119	3.683779573	11.16230048	3.20E-26	5.32E-25	48.53766802
SLPI	-1.733575361	4.129269528	-11.16186196	3.21E-26	5.34E-25	48.53368242
NKAIN1	1.248448096	2.394184446	11.16088804	3.24E-26	5.38E-25	48.52483114
ARSJ	-1.040852482	2.166585036	-11.12882279	4.35E-26	7.17E-25	48.23368408
RGS2	-1.021887334	5.231274332	-11.09845158	5.74E-26	9.42E-25	47.95840659
JPH4	1.32076127	3.661324204	11.09462167	5.95E-26	9.75E-25	47.92372693
RIMS4	1.109138979	3.111491884	11.08909451	6.26E-26	1.02E-24	47.87369226
ANGPTL4	-1.384057249	4.677147293	-11.0803111	6.78E-26	1.11E-24	47.79421273
MYT1	1.212842321	1.990595154	11.08001505	6.80E-26	1.11E-24	47.79153452
CPLX1	1.560752843	3.975338994	11.05815245	8.30E-26	1.34E-24	47.59388066
LINC00634	1.023604302	3.437856403	11.03420833	1.03E-25	1.66E-24	47.37769303
ATP2B2	1.004577096	2.778031312	11.02973653	1.08E-25	1.73E-24	47.33735091
CD52	-1.129716518	3.528728776	-10.9701453	1.85E-25	2.93E-24	46.8007479
ELAVL2	1.088256557	1.62748209	10.9678026	1.89E-25	2.99E-24	46.7796905
ADM	-1.31356418	4.559666386	-10.95773376	2.07E-25	3.26E-24	46.68921924
IGHA2	-1.43682931	1.674451286	-10.93861132	2.46E-25	3.84E-24	46.51754559
BCAN	1.685952513	8.424547175	10.89096983	3.79E-25	5.82E-24	46.09067748

ELFN2	1.176099623	1.839046212	10.8768947	4.30E-25	6.59E-24	45.96479374
IL32	-1.052888629	5.100934391	-10.87345363	4.43E-25	6.79E-24	45.93403382
DLGAP1	1.026320232	2.870993563	10.86337679	4.86E-25	7.40E-24	45.84399254
IGHG2	-1.904742058	2.72700227	-10.83856145	6.07E-25	9.21E-24	45.62248589
DIRAS3	-1.089621158	3.656826503	-10.83305021	6.38E-25	9.65E-24	45.57333597
SYP	1.228900482	5.181870647	10.82435342	6.90E-25	1.04E-23	45.49580979
JPH3	1.346628476	2.290247573	10.816718	7.39E-25	1.11E-23	45.42777828
CHRM1	1.103114884	1.443249432	10.76958473	1.13E-24	1.68E-23	45.00851156
PCDH15	1.268674516	1.789673767	10.74458475	1.41E-24	2.09E-23	44.78661177
COL6A1	-1.046308261	7.150371707	-10.73124832	1.59E-24	2.34E-23	44.66837512
TMEM151B	1.066634275	2.174108128	10.72024196	1.76E-24	2.57E-23	44.57086843
IGHG1	-2.102307562	3.490393709	-10.70510752	2.01E-24	2.93E-23	44.43689733
IGHA1	-1.922060897	3.108058657	-10.67496077	2.63E-24	3.82E-23	44.17040559
SLC2A3	-1.04505284	4.432089183	-10.65162873	3.24E-24	4.67E-23	43.96449312
NEAT1	-1.224152095	5.816132805	-10.58895455	5.66E-24	8.00E-23	43.41284255
IGFBP6	-1.242180234	4.06711122	-10.58783242	5.71E-24	8.07E-23	43.40298525
CARD16	-1.059493233	3.970551888	-10.56698018	6.88E-24	9.66E-23	43.21993564
TMEM179	1.175109093	3.087364092	10.52515458	9.96E-24	1.39E-22	42.85349371
TMEM176A	-1.128893204	5.193392334	-10.51085021	1.13E-23	1.57E-22	42.72839175
ST8SIA3	1.300757203	1.835661955	10.48258594	1.45E-23	2.00E-22	42.4815333
CD248	-1.031292855	4.099861599	-10.47709046	1.52E-23	2.09E-22	42.43358745
IGKC	-2.096381045	4.114596006	-10.47663547	1.53E-23	2.10E-22	42.42961859
COL4A1	-1.434278214	6.096696972	-10.46141026	1.75E-23	2.39E-22	42.29687625
C1QTNF4	1.130669087	2.710565106	10.44149324	2.08E-23	2.82E-22	42.12342256
OLIG2	1.408729628	5.890149843	10.42712722	2.36E-23	3.18E-22	41.99844865
LOXL1	-1.083641719	3.437921226	-10.41863724	2.54E-23	3.42E-22	41.92464619
ATP6V1G2	1.082868149	4.747666221	10.41690332	2.58E-23	3.47E-22	41.90957832
GSX1	1.336664238	2.065580213	10.37338791	3.78E-23	5.04E-22	41.53197837
CRB2	-1.08144892	3.654427628	-10.37230944	3.82E-23	5.08E-22	41.52263356
UNC5A	1.075427955	2.15297041	10.35137461	4.59E-23	6.08E-22	41.34136499
APOC1	-1.030609998	8.73210925	-10.34780492	4.73E-23	6.27E-22	41.3104807
IGLC3	-1.893818182	2.897335477	-10.33011743	5.52E-23	7.26E-22	41.15755759
NRXN1	1.119984535	3.161091276	10.31344204	6.39E-23	8.38E-22	41.01354656
ELAVL4	1.096042569	2.486135598	10.29014684	7.83E-23	1.02E-21	40.81262901
C21orf62	-1.113468621	3.576544068	-10.28880499	7.92E-23	1.03E-21	40.80106513
WNK2	1.112999089	2.425843265	10.28644531	8.08E-23	1.05E-21	40.78073219
NXPH1	1.172511872	2.741725556	10.22242684	1.41E-22	1.81E-21	40.23030289
SULF1	-1.246514506	3.382541134	-10.22200292	1.42E-22	1.81E-21	40.22666583
CADM2	1.081474751	3.434387425	10.2193914	1.45E-22	1.86E-21	40.20426217
SAPCD2	1.048252796	3.411792266	10.19269749	1.82E-22	2.32E-21	39.97548482
IGLC2	-1.750250619	2.633066535	-10.18466599	1.96E-22	2.49E-21	39.90673148
NPM2	1.083405582	2.10949156	10.11329169	3.62E-22	4.50E-21	39.29736205
MMP9	-1.732661264	4.163146615	-10.10664142	3.83E-22	4.76E-21	39.24073375
IGFBP3	-1.496373045	6.170721405	-10.10552599	3.87E-22	4.80E-21	39.23123819

LINC00844	1.487832009	4.95840964	10.1049913	3.89E-22	4.82E-21	39.22668663
GABRB3	1.052275436	1.832987391	10.09285828	4.32E-22	5.33E-21	39.12344922
PHACTR3	1.156055584	3.332447892	10.06684092	5.40E-22	6.63E-21	38.90235996
ESM1	-1.190487483	3.030499499	-10.06041812	5.70E-22	7.00E-21	38.84784096
CHGA	1.645340596	2.967510527	10.04625028	6.44E-22	7.88E-21	38.72766405
TNR	1.370815691	2.996676715	10.02996961	7.41E-22	9.03E-21	38.58970955
COL4A2	-1.25549499	6.43055467	-10.0071171	9.00E-22	1.09E-20	38.39632901
HP	-1.59751067	2.645938015	-10.00709999	9.01E-22	1.09E-20	38.3961843
ARHGDIG	1.370502439	3.538071508	9.995219642	9.97E-22	1.20E-20	38.29577203
FLNC	-1.163526523	4.080733506	-9.980771794	1.13E-21	1.36E-20	38.17377055
GRIA4	1.104532317	3.492782279	9.971404372	1.22E-21	1.47E-20	38.09473485
APOC2	-1.040929344	4.942742538	-9.936396106	1.65E-21	1.96E-20	37.79981558
CXCL2	-1.010878749	2.732835973	-9.876077617	2.75E-21	3.21E-20	37.29336924
S100A10	-1.273218745	7.53456747	-9.843477005	3.62E-21	4.19E-20	37.02054389
ATCAY	1.447842156	3.783813222	9.834824507	3.89E-21	4.48E-20	36.94823953
CNTN1	1.244760779	4.317986672	9.811905069	4.73E-21	5.40E-20	36.75692917
SDC1	-1.00535285	3.07587734	-9.809389596	4.83E-21	5.52E-20	36.73595138
VSTM2B	1.294907215	2.59500548	9.805187183	5.00E-21	5.70E-20	36.70091376
GADD45G	1.195641919	5.355904034	9.766826775	6.91E-21	7.83E-20	36.38157149
GRIN1	1.678675067	2.53439561	9.713189122	1.08E-20	1.21E-19	35.936528
CPLX2	1.664710949	3.1021676	9.695761948	1.25E-20	1.39E-19	35.79230338
IGHM	-1.304722381	1.878190903	-9.682940283	1.40E-20	1.55E-19	35.68631023
FAM163B	1.409626268	2.184454191	9.615755	2.44E-20	2.65E-19	35.13253504
ABCC8	1.084146666	2.65267656	9.605205438	2.67E-20	2.88E-19	35.04582917
DKK1	-1.348294117	1.794600618	-9.548756958	4.26E-20	4.55E-19	34.5830378
FBXL16	1.19262256	4.279208644	9.530584437	4.95E-20	5.26E-19	34.43446565
PSD	1.026254736	4.190994356	9.470783963	8.11E-20	8.48E-19	33.94699069
NETO1	1.195532131	2.128847418	9.46965565	8.19E-20	8.55E-19	33.9378142
C2orf80	1.060131171	3.245862715	9.464477805	8.55E-20	8.90E-19	33.89571329
ISLR	-1.030472916	3.903549988	-9.460503643	8.83E-20	9.18E-19	33.86341073
AGAP2	1.163343035	4.212349256	9.457003815	9.09E-20	9.43E-19	33.8349717
CAMKV	1.190888835	2.75922523	9.448410529	9.75E-20	1.01E-18	33.76517619
SFRP4	-1.029490818	3.808342379	-9.418088559	1.25E-19	1.29E-18	33.51926356
GDF15	-1.145323891	3.571172984	-9.410299448	1.33E-19	1.37E-18	33.45618555
DCN	-1.079461452	5.203381989	-9.392107323	1.55E-19	1.58E-18	33.30900838
GJB2	-1.056227316	2.149871324	-9.37472471	1.78E-19	1.80E-18	33.1685727
SMOC1	1.647351267	4.453856536	9.343435377	2.30E-19	2.31E-18	32.91625824
CDK5R2	1.031104976	2.54181119	9.328477615	2.60E-19	2.60E-18	32.79585632
MOXD1	-1.281011107	3.53943065	-9.302231281	3.22E-19	3.19E-18	32.58492622
FMOD	-1.191602407	3.536616931	-9.248843035	4.97E-19	4.86E-18	32.15720455
CHGB	1.141898384	3.141946869	9.205678845	7.04E-19	6.84E-18	31.81270804
HLA-DQA1	-1.430462583	3.794898147	-9.182872376	8.46E-19	8.15E-18	31.63116449
SNCB	1.493101738	3.90962156	9.117409728	1.43E-18	1.35E-17	31.11190813
CELF4	1.105140481	2.231586826	9.056383492	2.33E-18	2.17E-17	30.63030904

OMG	1.059243494	3.871524721	9.006454126	3.47E-18	3.18E-17	30.23806358
SYN1	1.195867655	3.508372107	9.005736414	3.49E-18	3.20E-17	30.23243695
KCNJ4	1.027748535	2.078515016	8.990047126	3.95E-18	3.61E-17	30.10952135
COL20A1	1.611992804	3.655556718	8.981405306	4.24E-18	3.86E-17	30.04188621
PDLIM4	-1.285978185	4.762760522	-8.948141943	5.51E-18	4.97E-17	29.78200257
VIPR2	1.159006952	1.879883593	8.946319036	5.59E-18	5.04E-17	29.76778111
CHRNA9	-1.001903314	1.907930196	-8.906111315	7.68E-18	6.85E-17	29.45464957
PPP1R1A	1.079680649	2.355741343	8.900648478	8.02E-18	7.14E-17	29.41218714
CXCL14	-1.395834044	5.084136979	-8.871366825	1.01E-17	8.91E-17	29.18491419
C7	-1.004400792	1.870964682	-8.862730442	1.08E-17	9.51E-17	29.117989
SVOP	1.046330255	1.465374849	8.840556116	1.29E-17	1.12E-16	28.94637928
PCP4L1	1.00726376	1.834177819	8.80307884	1.72E-17	1.49E-16	28.65707236
RARRES2	-1.377216713	5.601999472	-8.774913694	2.15E-17	1.84E-16	28.4402595
MAOB	-1.104922843	6.038878653	-8.734202913	2.95E-17	2.49E-16	28.12779783
CABP1	1.076336824	2.366024061	8.717581003	3.35E-17	2.81E-16	28.00053774
SNAP25	1.40913725	4.89637802	8.706331312	3.66E-17	3.07E-16	27.91451233
IGLV1-51	-1.068029933	1.49904032	-8.701751273	3.79E-17	3.17E-16	27.87951327
GABRD	1.047124746	2.623434672	8.67520963	4.66E-17	3.86E-16	27.6769661
STMN4	1.100990944	5.286902734	8.575724311	1.00E-16	8.11E-16	26.92195267
IGHG4	-1.026539018	1.351018667	-8.543602529	1.28E-16	1.03E-15	26.67959337
IGHG3	-1.315393646	1.90542142	-8.531510335	1.40E-16	1.12E-15	26.58853787
SNX22	1.076425159	3.611864918	8.440020284	2.82E-16	2.20E-15	25.90281644
NPPA	1.209778858	2.67849618	8.417577026	3.34E-16	2.60E-15	25.73547237
FOS	-1.028021539	7.129857381	-8.297648933	8.23E-16	6.21E-15	24.84709361
AEBP1	-1.077372404	6.845438013	-8.281736913	9.26E-16	6.95E-15	24.72996701
CA9	-1.231384227	3.209393092	-8.269946774	1.01E-15	7.57E-15	24.64329391
DCX	1.082536631	2.325295256	8.269283816	1.02E-15	7.60E-15	24.63842315
PLA2G5	-1.129198912	3.607090961	-8.24989792	1.17E-15	8.71E-15	24.4961291
CALY	1.333219146	2.696996817	8.237126312	1.29E-15	9.56E-15	24.40252669
POSTN	-1.669503111	3.720589293	-8.22950633	1.37E-15	1.01E-14	24.34673412
ASIC4	1.135205361	3.766568927	8.160271223	2.28E-15	1.65E-14	23.84165285
SYN2	1.121197714	2.39771052	8.159552127	2.30E-15	1.66E-14	23.83642445
TAC1	1.056786892	1.666043483	8.12321296	3.00E-15	2.15E-14	23.57268058
CCL4	-1.007003063	3.687921404	-8.00508155	7.12E-15	4.94E-14	22.72170715
CCK	1.407053413	2.626663424	7.991199044	7.88E-15	5.43E-14	22.62234933
METTL7B	-1.069243926	5.426649052	-7.950739759	1.06E-14	7.22E-14	22.33355942
NCAN	1.127377042	5.905131103	7.932237483	1.21E-14	8.22E-14	22.20188154
KCNQ2	1.114714392	4.577498471	7.930397959	1.22E-14	8.32E-14	22.18880325
ETNPPL	1.160322709	2.953600713	7.912240271	1.40E-14	9.45E-14	22.0598385
HPCA	1.107968951	3.717556486	7.872786819	1.85E-14	1.24E-13	21.78043182
MGST1	-1.008456075	5.44458883	-7.840714448	2.33E-14	1.55E-13	21.55411798
MGP	-1.018617369	5.982427597	-7.699605211	6.37E-14	4.07E-13	20.56719485
NTSR2	1.039411396	2.585804587	7.696194783	6.53E-14	4.15E-13	20.5435204
CA10	1.078016015	2.20586291	7.686044236	7.01E-14	4.45E-13	20.4731075

LUZP2	1.011866896	2.252813778	7.645382231	9.34E-14	5.86E-13	20.19179228
FXYD7	1.192068713	3.486310119	7.610974324	1.19E-13	7.39E-13	19.95468679
SH3GL2	1.013701901	3.10387972	7.607503616	1.22E-13	7.57E-13	19.93081809
HMX1	1.137892394	2.05929428	7.56293926	1.66E-13	1.02E-12	19.62512459
PACSIN1	1.104823663	2.131698828	7.547845663	1.85E-13	1.13E-12	19.52191881
STMN2	1.312246395	3.996157905	7.536495877	2.00E-13	1.22E-12	19.44442244
EEF1A2	1.063595323	5.987028683	7.465190515	3.27E-13	1.97E-12	18.9597203
SULT4A1	1.044801533	2.439859027	7.396874849	5.23E-13	3.07E-12	18.49886865
LINC00689	1.007032543	1.456097419	7.381108969	5.83E-13	3.40E-12	18.39300569
CRYM	1.122891283	2.201921145	7.344987152	7.46E-13	4.30E-12	18.15115727
GPR17	1.259176963	2.863092061	7.269861005	1.24E-12	6.98E-12	17.65128511
VEGFA	-1.19525276	6.84767871	-7.224509911	1.68E-12	9.34E-12	17.35158008
SLC17A7	1.319514718	2.764342587	7.146279943	2.84E-12	1.55E-11	16.83824059
IGLC1	-1.370165754	1.917885549	-7.136974107	3.03E-12	1.64E-11	16.77748472
TMSB15A	1.220852679	3.572646074	7.055359305	5.20E-12	2.77E-11	16.2474598
KLRC2	1.125851333	2.111348769	7.054448073	5.23E-12	2.79E-11	16.2415707
SYT1	1.037686657	2.918289453	6.903178081	1.41E-11	7.16E-11	15.2727704
NEFM	1.010640975	2.043176289	6.888994982	1.54E-11	7.81E-11	15.18283875
NRGN	1.220341892	5.638027394	6.857153203	1.89E-11	9.53E-11	14.98150444
HLA-DQB1	-1.192333569	3.930988022	-6.815110554	2.48E-11	1.24E-10	14.71687449
SLN	-1.038204471	3.767558459	-5.700601054	1.95E-08	7.15E-08	8.215631848
HLA-DRB5	-1.085303006	4.28901719	-5.370166118	1.16E-07	3.89E-07	6.484820389

Supplementary Table 5. Results after univariate Cox analysis of differential genes between two ERG clusters

ID	geneCluster	ID	geneCluster
TCGA_TCGA-19-1389	A	CGGA325_CGGA-1346	A
TCGA_TCGA-26-5132	A	CGGA325_CGGA-1393	A
TCGA_TCGA-28-2513	A	CGGA325_CGGA-272	A
TCGA_TCGA-76-4927	A	CGGA325_CGGA-318	A
TCGA_TCGA-26-5133	B	CGGA325_CGGA-773	B
TCGA_TCGA-76-4928	A	CGGA325_CGGA-719	B
TCGA_TCGA-06-5856	B	CGGA325_CGGA-1068	A
TCGA_TCGA-19-2619	B	CGGA325_CGGA-1105	A
TCGA_TCGA-41-3915	A	CGGA325_CGGA-1129	B
TCGA_TCGA-14-1825	B	CGGA325_CGGA-1136	A
TCGA_TCGA-15-1444	B	CGGA325_CGGA-1170	B
TCGA_TCGA-06-0210	A	CGGA325_CGGA-1175	B
TCGA_TCGA-14-1034	A	CGGA325_CGGA-1324	B
TCGA_TCGA-28-2499	B	CGGA325_CGGA-1375	B
TCGA_TCGA-06-2570	B	CGGA325_CGGA-1412	B
TCGA_TCGA-06-2565	B	CGGA325_CGGA-374	B

TCGA_TCGA-06-2559	A	CGGA325_CGGA-822	B
TCGA_TCGA-26-5134	B	CGGA325_CGGA-D34	B
TCGA_TCGA-26-1442	B	CGGA325_CGGA-D59	B
TCGA_TCGA-12-3652	B	CGGA325_CGGA-518	B
TCGA_TCGA-19-5960	B	CGGA325_CGGA-1099	A
TCGA_TCGA-06-2557	A	CGGA325_CGGA-1197	B
TCGA_TCGA-06-0686	B	CGGA325_CGGA-1450	B
TCGA_TCGA-19-2625	A	CGGA325_CGGA-1460	B
TCGA_TCGA-28-2514	B	CGGA325_CGGA-1475	A
TCGA_TCGA-06-2558	B	CGGA693_CGGA-1017	B
TCGA_TCGA-06-0882	A	CGGA693_CGGA-1036	A
TCGA_TCGA-28-5220	B	CGGA693_CGGA-1041	B
TCGA_TCGA-76-4929	B	CGGA693_CGGA-1075	A
TCGA_TCGA-19-2629	B	CGGA693_CGGA-1086	A
TCGA_TCGA-27-2528	B	CGGA693_CGGA-1103	B
TCGA_TCGA-06-1804	B	CGGA693_CGGA-1106	A
TCGA_TCGA-14-0817	A	CGGA693_CGGA-1130	B
TCGA_TCGA-06-0187	A	CGGA693_CGGA-1134	A
TCGA_TCGA-28-5218	A	CGGA693_CGGA-1135	A
TCGA_TCGA-06-5408	A	CGGA693_CGGA-1138	B
TCGA_TCGA-28-5204	B	CGGA693_CGGA-1142	B
TCGA_TCGA-12-0618	B	CGGA693_CGGA-1164	B
TCGA_TCGA-32-2634	B	CGGA693_CGGA-1172	B
TCGA_TCGA-28-5207	B	CGGA693_CGGA-120	A
TCGA_TCGA-28-5216	A	CGGA693_CGGA-1208	A
TCGA_TCGA-41-2571	B	CGGA693_CGGA-1236	B
TCGA_TCGA-12-1597	B	CGGA693_CGGA-1248	B
TCGA_TCGA-06-0219	B	CGGA693_CGGA-1255	A
TCGA_TCGA-06-5410	A	CGGA693_CGGA-1256	B
TCGA_TCGA-32-2638	A	CGGA693_CGGA-1257	B
TCGA_TCGA-14-0790	B	CGGA693_CGGA-1260	B
TCGA_TCGA-14-0787	A	CGGA693_CGGA-1262	A
TCGA_TCGA-06-0644	A	CGGA693_CGGA-1282	B
TCGA_TCGA-06-0744	B	CGGA693_CGGA-1325	B
TCGA_TCGA-06-0749	B	CGGA693_CGGA-1326	B
TCGA_TCGA-19-2620	B	CGGA693_CGGA-1337	B
TCGA_TCGA-32-2616	A	CGGA693_CGGA-1353	A
TCGA_TCGA-08-0386	B	CGGA693_CGGA-1354	A
TCGA_TCGA-06-0211	A	CGGA693_CGGA-1365	A
TCGA_TCGA-12-3653	B	CGGA693_CGGA-1371	A
TCGA_TCGA-27-2523	B	CGGA693_CGGA-1378	B
TCGA_TCGA-19-0957	B	CGGA693_CGGA-1380	B
TCGA_TCGA-12-0619	A	CGGA693_CGGA-1382	B
TCGA_TCGA-06-2569	B	CGGA693_CGGA-1387	A

TCGA_TCGA-06-0649	A	CGGA693_CGGA-139	B
TCGA_TCGA-12-0616	B	CGGA693_CGGA-1391	A
TCGA_TCGA-06-2562	A	CGGA693_CGGA-1392	A
TCGA_TCGA-28-5213	A	CGGA693_CGGA-1402	A
TCGA_TCGA-14-1402	B	CGGA693_CGGA-1403	A
TCGA_TCGA-06-5858	A	CGGA693_CGGA-1410	B
TCGA_TCGA-41-2572	A	CGGA693_CGGA-1415	A
TCGA_TCGA-27-2519	A	CGGA693_CGGA-1418	A
TCGA_TCGA-19-2624	B	CGGA693_CGGA-1419	B
TCGA_TCGA-27-1830	A	CGGA693_CGGA-1420	B
TCGA_TCGA-06-0878	A	CGGA693_CGGA-1422	A
TCGA_TCGA-06-0646	A	CGGA693_CGGA-1425	B
TCGA_TCGA-06-0157	B	CGGA693_CGGA-1426	B
TCGA_TCGA-27-1835	B	CGGA693_CGGA-1429	B
TCGA_TCGA-27-2524	A	CGGA693_CGGA-1430	B
TCGA_TCGA-14-1829	A	CGGA693_CGGA-1433	A
TCGA_TCGA-76-4932	A	CGGA693_CGGA-1441	A
TCGA_TCGA-06-0190	A	CGGA693_CGGA-1444	B
TCGA_TCGA-06-0645	A	CGGA693_CGGA-1451	A
TCGA_TCGA-06-5411	B	CGGA693_CGGA-1452	A
TCGA_TCGA-06-2567	A	CGGA693_CGGA-1457	A
TCGA_TCGA-26-5139	A	CGGA693_CGGA-1461	A
TCGA_TCGA-32-5222	A	CGGA693_CGGA-1462	A
TCGA_TCGA-02-0047	B	CGGA693_CGGA-1467	B
TCGA_TCGA-14-0736	B	CGGA693_CGGA-1472	B
TCGA_TCGA-06-5859	B	CGGA693_CGGA-1476	B
TCGA_TCGA-27-2521	B	CGGA693_CGGA-1478	B
TCGA_TCGA-06-0743	B	CGGA693_CGGA-1480	A
TCGA_TCGA-14-1823	A	CGGA693_CGGA-1481	A
TCGA_TCGA-14-0781	A	CGGA693_CGGA-1486	A
TCGA_TCGA-06-0171	A	CGGA693_CGGA-1491	B
TCGA_TCGA-16-0846	B	CGGA693_CGGA-1492	A
TCGA_TCGA-06-0238	B	CGGA693_CGGA-1494	B
TCGA_TCGA-27-2526	B	CGGA693_CGGA-1496	B
TCGA_TCGA-28-2510	B	CGGA693_CGGA-1498	A
TCGA_TCGA-32-4213	A	CGGA693_CGGA-1500	B
TCGA_TCGA-06-0745	B	CGGA693_CGGA-1501	B
TCGA_TCGA-12-5295	A	CGGA693_CGGA-1503	A
TCGA_TCGA-27-1831	A	CGGA693_CGGA-1505	A
TCGA_TCGA-27-1832	A	CGGA693_CGGA-1507	A
TCGA_TCGA-06-0132	B	CGGA693_CGGA-1520	B
TCGA_TCGA-27-1834	B	CGGA693_CGGA-1521	A
TCGA_TCGA-28-1753	A	CGGA693_CGGA-1529	B
TCGA_TCGA-32-2615	A	CGGA693_CGGA-1534	B

TCGA_TCGA-32-1982	A	CGGA693_CGGA-1535	A
TCGA_TCGA-28-2509	A	CGGA693_CGGA-1537	A
TCGA_TCGA-06-0125	A	CGGA693_CGGA-1538	A
TCGA_TCGA-06-0158	A	CGGA693_CGGA-1539	B
TCGA_TCGA-14-0789	A	CGGA693_CGGA-1541	B
TCGA_TCGA-06-0174	B	CGGA693_CGGA-1542	B
TCGA_TCGA-28-5208	A	CGGA693_CGGA-1543	A
TCGA_TCGA-06-0141	A	CGGA693_CGGA-1546	A
TCGA_TCGA-06-0129	B	CGGA693_CGGA-1548	A
TCGA_TCGA-06-5416	B	CGGA693_CGGA-1551	A
TCGA_TCGA-32-1970	B	CGGA693_CGGA-1558	A
TCGA_TCGA-19-1787	A	CGGA693_CGGA-1559	B
TCGA_TCGA-06-0184	A	CGGA693_CGGA-1560	B
TCGA_TCGA-41-5651	B	CGGA693_CGGA-1564	B
TCGA_TCGA-02-2485	B	CGGA693_CGGA-1571	B
TCGA_TCGA-06-0138	B	CGGA693_CGGA-1572	B
TCGA_TCGA-06-0747	B	CGGA693_CGGA-1586	A
TCGA_TCGA-06-5414	A	CGGA693_CGGA-1595	B
TCGA_TCGA-06-0750	A	CGGA693_CGGA-1596	B
TCGA_TCGA-76-4925	A	CGGA693_CGGA-1597	A
TCGA_TCGA-06-0221	B	CGGA693_CGGA-1601	A
TCGA_TCGA-19-4065	A	CGGA693_CGGA-1603	A
TCGA_TCGA-06-0178	B	CGGA693_CGGA-1604	A
TCGA_TCGA-02-0055	A	CGGA693_CGGA-1605	B
TCGA_TCGA-02-2483	B	CGGA693_CGGA-1611	B
TCGA_TCGA-06-2563	B	CGGA693_CGGA-1612	A
TCGA_TCGA-06-0139	A	CGGA693_CGGA-1613	A
TCGA_TCGA-28-5209	A	CGGA693_CGGA-1615	B
TCGA_TCGA-02-2486	A	CGGA693_CGGA-1624	A
TCGA_TCGA-76-4931	B	CGGA693_CGGA-1626	A
TCGA_TCGA-06-2564	A	CGGA693_CGGA-1631	A
TCGA_TCGA-27-1837	B	CGGA693_CGGA-1634	A
TCGA_TCGA-41-4097	A	CGGA693_CGGA-1635	B
TCGA_TCGA-26-5136	A	CGGA693_CGGA-1641	B
TCGA_TCGA-15-0742	B	CGGA693_CGGA-1643	B
TCGA_TCGA-06-5417	B	CGGA693_CGGA-1644	A
TCGA_TCGA-26-5135	B	CGGA693_CGGA-1650	B
TCGA_TCGA-06-5418	A	CGGA693_CGGA-1654	A
TCGA_TCGA-28-5215	A	CGGA693_CGGA-1656	A
TCGA_TCGA-06-0152	A	CGGA693_CGGA-1658	A
TCGA_TCGA-19-1390	B	CGGA693_CGGA-1659	A
TCGA_TCGA-32-1980	B	CGGA693_CGGA-1663	A
TCGA_TCGA-06-0168	A	CGGA693_CGGA-1666	A
TCGA_TCGA-06-5412	A	CGGA693_CGGA-1678	A

TCGA_TCGA-32-2632	A	CGGA693_CGGA-1681	A
TCGA_TCGA-12-3650	B	CGGA693_CGGA-1682	A
TCGA_TCGA-06-0156	A	CGGA693_CGGA-1684	A
TCGA_TCGA-06-0130	A	CGGA693_CGGA-1687	A
TCGA_TCGA-06-5413	A	CGGA693_CGGA-1690	A
TCGA_TCGA-12-0821	B	CGGA693_CGGA-1694	A
TCGA_TCGA-12-5299	A	CGGA693_CGGA-1697	B
TCGA_TCGA-06-2561	B	CGGA693_CGGA-1698	A
TCGA_TCGA-14-0871	B	CGGA693_CGGA-1699	B
TCGA_TCGA-14-2554	A	CGGA693_CGGA-1702	B
TCGA_TCGA-28-1747	B	CGGA693_CGGA-1706	A
TCGA_TCGA-16-1045	A	CGGA693_CGGA-1708	A
TCGA_TCGA-76-4926	B	CGGA693_CGGA-1709	A
CGGA325_CGGA-1001	A	CGGA693_CGGA-1713	B
CGGA325_CGGA-1007	B	CGGA693_CGGA-1722	A
CGGA325_CGGA-1011	A	CGGA693_CGGA-1727	B
CGGA325_CGGA-1015	A	CGGA693_CGGA-1728	B
CGGA325_CGGA-1023	B	CGGA693_CGGA-1729	B
CGGA325_CGGA-1024	B	CGGA693_CGGA-1735	B
CGGA325_CGGA-1026	B	CGGA693_CGGA-1736	A
CGGA325_CGGA-1035	A	CGGA693_CGGA-1740	B
CGGA325_CGGA-1039	B	CGGA693_CGGA-1744	A
CGGA325_CGGA-1045	A	CGGA693_CGGA-1749	B
CGGA325_CGGA-1049	A	CGGA693_CGGA-1750	B
CGGA325_CGGA-1072	B	CGGA693_CGGA-1758	B
CGGA325_CGGA-1077	A	CGGA693_CGGA-1764	B
CGGA325_CGGA-1083	A	CGGA693_CGGA-1767	A
CGGA325_CGGA-1091	A	CGGA693_CGGA-1769	B
CGGA325_CGGA-1109	B	CGGA693_CGGA-1770	B
CGGA325_CGGA-1124	A	CGGA693_CGGA-1773	A
CGGA325_CGGA-1139	B	CGGA693_CGGA-1776	B
CGGA325_CGGA-1171	A	CGGA693_CGGA-1780	A
CGGA325_CGGA-1180	A	CGGA693_CGGA-1785	B
CGGA325_CGGA-1214	B	CGGA693_CGGA-1807	B
CGGA325_CGGA-1216	A	CGGA693_CGGA-1811	A
CGGA325_CGGA-1224	A	CGGA693_CGGA-1812	A
CGGA325_CGGA-1234	A	CGGA693_CGGA-1814	A
CGGA325_CGGA-1237	B	CGGA693_CGGA-1815	A
CGGA325_CGGA-1240	A	CGGA693_CGGA-1817	A
CGGA325_CGGA-1251	B	CGGA693_CGGA-1819	A
CGGA325_CGGA-1258	A	CGGA693_CGGA-1820	A
CGGA325_CGGA-1270	A	CGGA693_CGGA-1826	B
CGGA325_CGGA-1299	A	CGGA693_CGGA-1833	A
CGGA325_CGGA-1313	A	CGGA693_CGGA-1840	B

CGGA325_CGGA-1320	B
CGGA325_CGGA-1332	B
CGGA325_CGGA-1338	A
CGGA325_CGGA-1342	A
CGGA325_CGGA-274	A
CGGA325_CGGA-413	A
CGGA325_CGGA-483	A
CGGA325_CGGA-494	B
CGGA325_CGGA-499	B
CGGA325_CGGA-525	A
CGGA325_CGGA-604	A
CGGA325_CGGA-658	B
CGGA325_CGGA-676	A
CGGA325_CGGA-679	A
CGGA325_CGGA-680	B
CGGA325_CGGA-700	B
CGGA325_CGGA-731	A
CGGA325_CGGA-782	A
CGGA325_CGGA-789	A
CGGA325_CGGA-802	A
CGGA325_CGGA-804	B
CGGA325_CGGA-808	B
CGGA325_CGGA-837	A
CGGA325_CGGA-842	A
CGGA325_CGGA-848	A
CGGA325_CGGA-850	B
CGGA325_CGGA-859	A
CGGA325_CGGA-876	A
CGGA325_CGGA-878	A
CGGA325_CGGA-902	A
CGGA325_CGGA-D03	A
CGGA325_CGGA-D09	B
CGGA325_CGGA-D35	A
CGGA325_CGGA-D37	A
CGGA325_CGGA-D57	A
CGGA325_CGGA-342	A
CGGA325_CGGA-1219	B
CGGA325_CGGA-1287	B
CGGA325_CGGA-1314	B
CGGA325_CGGA-1384	B
CGGA325_CGGA-1409	B
CGGA325_CGGA-491	B
CGGA325_CGGA-710	B
CGGA325_CGGA-747	B
CGGA693_CGGA-1857	A
CGGA693_CGGA-1865	A
CGGA693_CGGA-1866	B
CGGA693_CGGA-1870	B
CGGA693_CGGA-1886	B
CGGA693_CGGA-1899	B
CGGA693_CGGA-1901	A
CGGA693_CGGA-1908	B
CGGA693_CGGA-1911	A
CGGA693_CGGA-1912	A
CGGA693_CGGA-1916	B
CGGA693_CGGA-1946	A
CGGA693_CGGA-1953	A
CGGA693_CGGA-1955	B
CGGA693_CGGA-1972	A
CGGA693_CGGA-1976	A
CGGA693_CGGA-1985	B
CGGA693_CGGA-2003	B
CGGA693_CGGA-2008	A
CGGA693_CGGA-2024	B
CGGA693_CGGA-2038	B
CGGA693_CGGA-2039	B
CGGA693_CGGA-2047	B
CGGA693_CGGA-2053	B
CGGA693_CGGA-2056	A
CGGA693_CGGA-2062	A
CGGA693_CGGA-2075	A
CGGA693_CGGA-2078	A
CGGA693_CGGA-2082	A
CGGA693_CGGA-2088	A
CGGA693_CGGA-2106	B
CGGA693_CGGA-2115	B
CGGA693_CGGA-487	A
CGGA693_CGGA-509	B
CGGA693_CGGA-530	A
CGGA693_CGGA-568	B
CGGA693_CGGA-777	A
CGGA693_CGGA-831	A
CGGA693_CGGA-869	A
CGGA693_CGGA-D53	B
CGGA693_CGGA-P100	B
CGGA693_CGGA-P102	B
CGGA693_CGGA-P106	A
CGGA693_CGGA-P109	B

CGGA325_CGGA-761	B
CGGA325_CGGA-1008	A
CGGA325_CGGA-1053	A
CGGA325_CGGA-1070	B
CGGA325_CGGA-1073	A
CGGA325_CGGA-1074	A
CGGA325_CGGA-1114	A
CGGA325_CGGA-1275	A
CGGA325_CGGA-759	B
CGGA325_CGGA-D30	B
CGGA325_CGGA-1019	A
CGGA325_CGGA-1078	A
CGGA325_CGGA-1177	A
CGGA325_CGGA-1271	A
CGGA325_CGGA-1285	A
CGGA325_CGGA-1343	A
CGGA325_CGGA-1370	B
CGGA325_CGGA-624	A
CGGA325_CGGA-669	B
CGGA325_CGGA-D02	A
CGGA325_CGGA-D26	A
CGGA325_CGGA-D32	A
CGGA325_CGGA-D36	A
CGGA325_CGGA-D51	A
CGGA325_CGGA-1060	B
CGGA325_CGGA-1227	B
CGGA325_CGGA-1381	B
CGGA325_CGGA-1394	B
CGGA325_CGGA-545	B
CGGA325_CGGA-899	B
CGGA325_CGGA-D38	B
CGGA325_CGGA-J100	B
CGGA325_CGGA-1116	B
CGGA325_CGGA-1119	B
CGGA325_CGGA-1188	B
CGGA325_CGGA-1218	A
CGGA325_CGGA-1272	B
CGGA325_CGGA-1283	B
CGGA325_CGGA-1301	B

CGGA693_CGGA-P112	B
CGGA693_CGGA-P116	A
CGGA693_CGGA-P136	B
CGGA693_CGGA-P143	A
CGGA693_CGGA-P15	B
CGGA693_CGGA-P154	B
CGGA693_CGGA-P16	B
CGGA693_CGGA-P160	A
CGGA693_CGGA-P164	A
CGGA693_CGGA-P175	A
CGGA693_CGGA-P178	B
CGGA693_CGGA-P180	A
CGGA693_CGGA-P182	A
CGGA693_CGGA-P199	A
CGGA693_CGGA-P205	A
CGGA693_CGGA-P22	B
CGGA693_CGGA-P25	A
CGGA693_CGGA-P28	A
CGGA693_CGGA-P280	B
CGGA693_CGGA-P283	A
CGGA693_CGGA-P295	B
CGGA693_CGGA-P335	B
CGGA693_CGGA-P385	B
CGGA693_CGGA-P401	A
CGGA693_CGGA-P411	B
CGGA693_CGGA-P415	A
CGGA693_CGGA-P499	A
CGGA693_CGGA-P512	B
CGGA693_CGGA-P585	A
CGGA693_CGGA-P596	B
CGGA693_CGGA-P609	B
CGGA693_CGGA-P610	B
CGGA693_CGGA-P619	A
CGGA693_CGGA-P625	A
CGGA693_CGGA-P7	A
CGGA693_CGGA-P87	B
CGGA693_CGGA-P89	A
CGGA693_CGGA-P99	B

Supplementary Table 6. Detailed cohort information for the 2 ERG gene clusters by the consensus clustering algorithm

id	HR	HR.95L	HR.95H	pvalue
LAIR1	1.131054174	1.041041476	1.228849737	0.003607611
SIGLEC9	1.153659066	1.046874313	1.271336227	0.003922441
FCGR2A	1.153333761	1.068199044	1.245253654	0.000266121
CD14	1.11410795	1.045384387	1.187349401	0.000880144
CTSB	1.199814988	1.099850041	1.30886571	4.06E-05
FCGR3A	1.117438641	1.040906025	1.199598318	0.002158486
CD163	1.095400161	1.044028075	1.149300044	0.00020075
ARPC1B	1.155064171	1.051038962	1.26938514	0.002755715
IFI30	1.153503101	1.076130322	1.236438911	5.55E-05
MSR1	1.130878745	1.054303734	1.213015468	0.000585644
SERPINA1	1.0935489	1.022193998	1.169884775	0.00938859
SIGLEC7	1.132249079	1.033229329	1.240758407	0.007812919
ITGB2	1.115820121	1.032374599	1.206010439	0.005720709
PLAUR	1.169283849	1.091954295	1.252089695	7.47E-06
CTSZ	1.109558369	1.033730168	1.190948869	0.003996102
SLC11A1	1.15352088	1.079235829	1.232919058	2.61E-05
DOK3	1.218490718	1.105093449	1.343524055	7.34E-05
PTPN7	1.171024795	1.045174249	1.31202914	0.00649605
SLC7A7	1.151324847	1.036141552	1.279312562	0.008790041
CTSC	1.125882	1.041806078	1.216743025	0.002751198
FCGR2B	1.101023872	1.037703169	1.168208408	0.001449397
VSIG4	1.115389269	1.043875235	1.191802602	0.001237655
ICAM1	1.142576255	1.071613694	1.218237976	4.62E-05
LCP2	1.140934788	1.036703244	1.255645912	0.006988256
MYOF	1.167814569	1.060975348	1.285412399	0.001529314
RAC2	1.16217545	1.060897951	1.273121298	0.001234805
FPR3	1.12356954	1.036519851	1.217929892	0.004629567
C5AR1	1.116284803	1.038605433	1.199773968	0.002796406
SLAMF8	1.115612858	1.03132675	1.206787324	0.006342033
NCF2	1.170348508	1.061080038	1.290869285	0.001657945
MYO1G	1.132203009	1.040744064	1.231699222	0.003861658
HK3	1.124711226	1.047142163	1.208026366	0.00126691
C1R	1.137290706	1.068470747	1.210543342	5.36E-05
TLR2	1.134412064	1.035882444	1.242313487	0.006519853
CD68	1.128491029	1.044532842	1.219197664	0.002180185
SLC16A3	1.161881344	1.085612528	1.243508363	1.48E-05
CTSL	1.14499554	1.044731407	1.254882142	0.003781038
MS4A4A	1.102725037	1.026480747	1.184632553	0.007474495
C1S	1.133536816	1.059617241	1.212613068	0.000269452
C1QB	1.10503475	1.024937552	1.191391414	0.009279989
SOCS3	1.133077724	1.079389918	1.18943591	4.54E-07

THBD	1.118693385	1.034330156	1.209937545	0.005051677
DPYD	1.099905447	1.02742816	1.177495458	0.006181689
CEBPB	1.154711306	1.056482052	1.262073688	0.001517901
TREM1	1.076445226	1.025539596	1.129877705	0.002880104
FCGR2C	1.118734851	1.048314707	1.193885443	0.000718575
CSTA	1.117592525	1.036067077	1.205533001	0.004017381
CDCP1	1.149729115	1.042439576	1.268061064	0.005245789
S100A9	1.074981938	1.026109583	1.126182024	0.00232162
NAMPT	1.109395357	1.045439086	1.177264247	0.000610859
CLCF1	1.162498912	1.081467144	1.249602197	4.42E-05
C1RL	1.172576483	1.08250757	1.270139486	9.46E-05
CTSD	1.23074405	1.10584821	1.369745778	0.000143058
C2	1.11698094	1.038451564	1.201448833	0.002935653
CHI3L1	1.08332407	1.04871997	1.119069984	1.35E-06
MVP	1.140554056	1.032577589	1.259821604	0.00954947
COL8A1	1.085945749	1.034743901	1.139681199	0.000819942
GPR84	1.137353415	1.035830024	1.248827279	0.006978059
GCNT1	1.145124225	1.043770589	1.25631964	0.004157102
DENND2D	1.223796787	1.111698157	1.347198938	3.78E-05
TNFRSF1A	1.223362068	1.102427599	1.357562847	0.000146961
ALOX5AP	1.094105344	1.022964393	1.170193715	0.008745304
STAB1	1.094189209	1.022699769	1.170675952	0.009026115
LILRA6	1.239932344	1.117113685	1.376254036	5.32E-05
CFI	1.193032831	1.113470994	1.27827967	5.38E-07
ITGA5	1.178958309	1.089735281	1.27548655	4.13E-05
CSF1	1.153628003	1.039058325	1.280830475	0.007408147
TYMP	1.103435149	1.035243114	1.176119031	0.002493398
SAT1	1.168629327	1.059325547	1.289211335	0.001869264
LILRB3	1.1651941	1.06788476	1.271370601	0.000590167
SERPINE1	1.098235666	1.044385944	1.15486194	0.000259181
SOD2	1.103812233	1.041201741	1.170187675	0.000915966
CD44	1.166982524	1.09022621	1.249142791	8.65E-06
TNFAIP3	1.169957456	1.071878625	1.277010677	0.000441722
CASP4	1.171403662	1.07188591	1.280161	0.000478589
COL1A1	1.083601047	1.039152324	1.12995102	0.000171879
CCL2	1.113800696	1.060531915	1.169745082	1.63E-05
SRPX2	1.105197062	1.041649213	1.172621773	0.000931284
STEAP3	1.143658811	1.069289412	1.223200623	9.12E-05
ABCC3	1.122927112	1.065864225	1.183044959	1.32E-05
BCL3	1.180422066	1.091328483	1.276789047	3.43E-05
AQP9	1.108916319	1.033088543	1.190309786	0.004226491
SPP1	1.103009794	1.051612894	1.156918685	5.65E-05
SERPINB1	1.144611931	1.035025374	1.265801309	0.008528167
SPOCD1	1.095648614	1.04618302	1.147453038	0.000106456

S100A8	1.076505787	1.027632858	1.127703052	0.001872083
NFKBIZ	1.103595248	1.026985789	1.185919497	0.007244759
BACE2	1.243039385	1.130494646	1.366788351	7.02E-06
RDH10	1.147934496	1.072268536	1.228939919	7.32E-05
ANXA2	1.191467714	1.115565918	1.272533779	1.83E-07
FAM20A	1.114307901	1.035232555	1.19942335	0.003952072
CLEC5A	1.1619845	1.081109799	1.248909204	4.53E-05
CEBPD	1.141998917	1.05586295	1.235161747	0.000904953
AMPD3	1.137453005	1.032132452	1.253520647	0.009378731
OSMR	1.203179945	1.111701106	1.302186328	4.55E-06
TGFBI	1.108929973	1.048755874	1.172556661	0.000280867
BCL2A1	1.102058259	1.03386978	1.174744083	0.002862855
COL1A2	1.093837019	1.043469495	1.146635749	0.000192151
FOSL2	1.110497988	1.025866543	1.202111316	0.00955919
TNFAIP2	1.121729428	1.047953569	1.200699102	0.000935059
RUNX1	1.191951575	1.095131405	1.297331582	4.86E-05
NRP1	1.128057309	1.037322613	1.226728576	0.00485591
IL4I1	1.157967593	1.071689604	1.251191522	0.000205198
LIF	1.104394512	1.047353394	1.164542211	0.000242624
PTX3	1.099673407	1.048550443	1.153288915	9.16E-05
PLAU	1.135716971	1.069022504	1.206572391	3.76E-05
FCGBP	1.072307981	1.017028127	1.130592534	0.009731871
COL3A1	1.072052984	1.027627543	1.118398984	0.001272811
COL6A3	1.084889202	1.034813663	1.137387939	0.000726716
CD93	1.109257242	1.027697354	1.197289868	0.007787998
CP	1.15325913	1.092275141	1.217647981	2.69E-07
FAM20C	1.215551047	1.128222505	1.309639137	2.87E-07
OLFML2B	1.115147665	1.033458718	1.203293652	0.004987105
MMP19	1.111158752	1.043083938	1.183676335	0.001084424
ZDHHC22	0.900427791	0.84906418	0.954898611	0.00046531
HSPA7	1.124892615	1.057005869	1.197139422	0.000210894
PRSS23	1.159592515	1.073279195	1.252847169	0.000175503
ACSL1	1.140273036	1.038112545	1.252487124	0.006125233
THBS1	1.095225729	1.039201947	1.154269776	0.000685496
PYGL	1.208054261	1.102509396	1.323703094	5.08E-05
FN1	1.142957336	1.073628202	1.216763373	2.85E-05
CLIC1	1.201692693	1.104680919	1.307223926	1.89E-05
CAPG	1.169894795	1.073024854	1.275509906	0.000373366
FTL	1.130955033	1.046260811	1.222505205	0.001944065
S100A11	1.153276161	1.075120997	1.23711276	6.80E-05
IRF1	1.134475136	1.033527163	1.245283028	0.007965678
FSD1	0.876849029	0.801824149	0.958893819	0.003980101
MAN1C1	1.142064204	1.053829474	1.237686625	0.001203732
PLIN2	1.151210952	1.067694094	1.241260641	0.000247742

COL5A1	1.124092637	1.063258601	1.188407274	3.78E-05
GPNMB	1.109536602	1.049496487	1.173011521	0.000250266
NPC2	1.12914859	1.03283155	1.234447707	0.007583039
IBSP	1.103296632	1.048390285	1.161078537	0.000160407
RAB42	1.271146812	1.154541703	1.399528672	1.02E-06
MSN	1.214032949	1.11479628	1.322103444	8.29E-06
FCER1G	1.123637884	1.047050151	1.205827719	0.001210277
A2M	1.128941841	1.029692671	1.237757357	0.009789204
BIRC3	1.128892424	1.042398281	1.222563513	0.002873559
TMBIM1	1.172784928	1.063823769	1.292906331	0.001357513
PROS1	1.14208961	1.03928947	1.25505811	0.005766937
TPP1	1.158686377	1.048195782	1.280823816	0.003970033
SHC1	1.175836339	1.073855027	1.28750256	0.000466453
LOXL2	1.163938231	1.087593301	1.245642286	1.16E-05
KCNIP2	0.908595944	0.848662587	0.972761852	0.005902415
ANXA1	1.145450857	1.081137326	1.213590201	4.10E-06
S100A4	1.105531236	1.038888198	1.176449319	0.00156358
NNMT	1.068416244	1.025472239	1.113158627	0.001568618
GPX8	1.129238471	1.049822468	1.214662061	0.001087815
PLTP	1.138797792	1.041979113	1.244612675	0.004142922
EFEMP1	1.123317563	1.054580564	1.196534804	0.000306761
ABCA1	1.122898864	1.037401295	1.215442726	0.004121553
TIMP1	1.086812071	1.042375802	1.133142648	9.29E-05
HSPA6	1.135399687	1.065060633	1.210384093	9.95E-05
ENHO	0.889064699	0.836866969	0.944518147	0.000139532
GDAP1L1	0.92640103	0.874270462	0.981640013	0.009680087
PDPN	1.125171029	1.066950193	1.186568831	1.36E-05
WWTR1	1.200569761	1.10708031	1.301954101	9.90E-06
BHLHE40	1.154300983	1.060047917	1.256934463	0.000960873
RUNDC3A	0.90329492	0.849850541	0.960100245	0.001081242
MMP14	1.147327329	1.078685855	1.220336758	1.26E-05
ACTN1	1.226268852	1.128831689	1.332116481	1.37E-06
TMEM176B	1.113828374	1.039127616	1.193899216	0.002337798
TAGLN2	1.257233361	1.159203067	1.36355378	3.26E-08
FNDC3B	1.140591201	1.046275416	1.243409018	0.002815312
TMEM145	0.895176987	0.826002796	0.970144219	0.006962134
LUM	1.079647756	1.028228863	1.133637966	0.002083354
CHI3L2	1.088451644	1.044459943	1.134296236	5.66E-05
FZD7	1.119748842	1.040896487	1.204574599	0.002398963
FOSL1	1.117128946	1.037035261	1.203408533	0.003522563
CA12	1.107521368	1.042467828	1.17663447	0.000944377
TNFRSF12A	1.170057668	1.097852677	1.247011531	1.35E-06
TUBA1C	1.179611074	1.097935156	1.267362903	6.42E-06
ADAM12	1.130623423	1.049946358	1.217499651	0.001152662

MRC2	1.166985961	1.086709544	1.253192485	2.17E-05
RIPPLY2	0.91353615	0.855112863	0.975951051	0.007320957
CXCR4	1.137414182	1.045721304	1.237147046	0.00267791
LGALS3	1.103708553	1.040509079	1.170746699	0.001038487
MXRA5	1.187282673	1.101460277	1.279792086	7.31E-06
CASKIN1	0.897574044	0.832552947	0.967673188	0.004855882
G0S2	1.096957999	1.041773532	1.15506568	0.000441506
COL6A2	1.100028029	1.047781752	1.154879499	0.000123047
ACTA2	1.15651901	1.073015051	1.246521397	0.000142935
LTF	1.052827691	1.022358344	1.084205116	0.000591015
ADAMTS1	1.180339587	1.092050809	1.275766227	2.92E-05
RARRES1	1.113547511	1.043540691	1.188250799	0.001168591
TAGLN	1.123080571	1.054975144	1.195582641	0.000276184
VASN	1.150793657	1.070832742	1.236725391	0.000132088
SERPING1	1.11838266	1.047003608	1.19462795	0.000884184
TNC	1.143659752	1.077769338	1.213578437	9.27E-06
IL6	1.10418863	1.039817149	1.172545126	0.001220665
UPP1	1.119704172	1.034265168	1.212201157	0.005239956
COL5A2	1.120972069	1.056195524	1.189721363	0.000169749
SEZ6L	0.918345923	0.868850777	0.970660621	0.00258318
MMD2	0.881651342	0.822214114	0.945385241	0.000404558
ZFP36	1.136850627	1.058984365	1.220442332	0.000395448
SCG3	0.896803731	0.846486866	0.950111531	0.000218127
RCAN1	1.11822867	1.039080952	1.203405141	0.002849547
IGFBP7	1.140930724	1.049077068	1.240826777	0.002078732
CNN2	1.106462145	1.025034222	1.19435864	0.009488347
ITGA3	1.100911013	1.027695289	1.179342818	0.006181467
CAV1	1.121264254	1.044101459	1.204129653	0.001653539
STC1	1.192736696	1.10304936	1.289716379	9.91E-06
GBP1	1.100067542	1.026575037	1.178821376	0.006862736
VIM	1.238941888	1.141018233	1.345269478	3.39E-07
EMILIN1	1.125313201	1.055061291	1.200242877	0.000331159
EMP1	1.09057586	1.031039755	1.153549804	0.002468435
EMP3	1.164107123	1.092971273	1.239872838	2.32E-06
SERPINH1	1.195238758	1.109508973	1.287592731	2.65E-06
C6orf141	1.092860566	1.032740831	1.156480098	0.002098629
CKMT1B	0.90932604	0.84876652	0.974206484	0.006869257
MYL9	1.129879586	1.048284203	1.217826116	0.001408177
SERPINA3	1.158973979	1.094808528	1.226900092	3.83E-07
MMP11	1.155021093	1.067121967	1.250160494	0.000358882
GEM	1.158180944	1.064408149	1.260214985	0.000652178
LAMB1	1.153147313	1.072523014	1.239832346	0.00011658
PLP2	1.164533736	1.088509984	1.245867142	9.77E-06
ATP1A3	0.933060594	0.889155631	0.979133508	0.004840257

TSPAN7	0.886277339	0.824198555	0.953031908	0.001120567
BEX1	0.925774176	0.8736186	0.981043472	0.009137861
CKMT1A	0.905001827	0.842625079	0.97199612	0.006153449
OLIG1	0.922223707	0.879681046	0.966823794	0.000779081
CBLN1	0.862584397	0.79442389	0.936592985	0.000432047
INA	0.927090066	0.880823431	0.975786929	0.003750982
FSTL1	1.158881834	1.070860393	1.254138368	0.000253556
NDRG1	1.105085551	1.034803319	1.180141243	0.002878891
LGALS1	1.192999283	1.100959055	1.292734079	1.65E-05
SNAP91	0.925436648	0.874511224	0.979327613	0.007289735
PODNL1	1.152977688	1.075036343	1.236569869	6.72E-05
ALDOC	0.897671943	0.842562299	0.956386154	0.000839373
NDRG2	0.870068501	0.815756512	0.92799651	2.31E-05
PHYHIPL	0.874629412	0.819133812	0.933884792	6.20E-05
MYBPH	1.097539223	1.0384357	1.160006678	0.000982966
NEU4	0.923240128	0.873219396	0.976126204	0.004951324
FBLIM1	1.15170553	1.075546544	1.233257302	5.20E-05
RPRM	0.908031942	0.855372419	0.963933358	0.001550509
ANGPTL4	1.100037316	1.039215397	1.164418946	0.001018142
CPLX1	0.923735805	0.877564707	0.972336091	0.002426996
LINC00634	0.8574224	0.795094526	0.924636189	6.47E-05
ATP2B2	0.873649719	0.808840324	0.943652052	0.000593101
ADM	1.096436465	1.032082605	1.164803007	0.002852357
ELFN2	0.915881973	0.858142515	0.977506385	0.008175633
IL32	1.101126304	1.023539976	1.184593828	0.00976355
DLGAP1	0.836821543	0.776602695	0.901709843	2.94E-06
DIRAS3	1.103304354	1.027836357	1.184313524	0.006538963
SYP	0.897867386	0.841682482	0.957802806	0.001084529
PCDH15	0.916735815	0.863660971	0.973072284	0.004276072
COL6A1	1.149889454	1.072710307	1.232621472	8.15E-05
IGHG1	1.052663724	1.015176749	1.091534963	0.005535019
NEAT1	1.089670888	1.024152898	1.159380251	0.006641638
TMEM176A	1.113478627	1.041297702	1.190663008	0.001669985
CD248	1.126876884	1.050563161	1.208734096	0.00084189
COL4A1	1.106672211	1.051817569	1.164387646	9.32E-05
OLIG2	0.917675249	0.870452951	0.96745937	0.001436148
LOXL1	1.180957346	1.105939311	1.261064002	6.80E-07
ATP6V1G2	0.904312376	0.845223316	0.967532316	0.003530683
CRB2	1.135840614	1.060300237	1.216762813	0.0002862
UNC5A	0.902406257	0.841614644	0.967588978	0.003903047
NRXN1	0.904909686	0.847352653	0.966376321	0.002882529
C21orf62	1.10317692	1.032198844	1.17903573	0.003804073
SULF1	1.097117068	1.034496112	1.163528646	0.001995006
MMP9	1.072916038	1.030049361	1.117566659	0.000716604

IGFBP3	1.073269947	1.022103732	1.126997527	0.004551024
LINC00844	0.91252282	0.870007932	0.957115293	0.000169531
GABRB3	0.910483616	0.849125503	0.976275489	0.008426836
ESM1	1.099674293	1.036761779	1.166404448	0.001571945
TNR	0.898372146	0.851170502	0.948191356	9.95E-05
COL4A2	1.118340252	1.058324502	1.181759391	7.06E-05
FLNC	1.149254914	1.082279678	1.220374812	5.60E-06
GRIA4	0.894367176	0.839131834	0.953238352	0.000598349
CXCL2	1.106471707	1.030234487	1.188350471	0.00547398
S100A10	1.102929197	1.041424219	1.168066569	0.000818721
SDC1	1.099871488	1.030633617	1.173760754	0.004110839
CPLX2	0.944141646	0.905062695	0.984907953	0.007697707
ABCC8	0.916754593	0.860714355	0.976443553	0.006919775
GJB2	1.098086165	1.031193397	1.169318219	0.003524778
MOXD1	1.093425241	1.035856321	1.154193621	0.001209832
FMOD	1.115070823	1.056411267	1.176987579	7.81E-05
CHGB	0.91234687	0.859080134	0.968916377	0.002801219
OMG	0.920419366	0.864959051	0.979435742	0.008915719
PDLIM4	1.083027047	1.028501875	1.140442826	0.002475986
VIPR2	0.912375881	0.862898282	0.964690468	0.001265708
CHRNA9	1.099988331	1.032181023	1.17225012	0.003328244
PPP1R1A	0.918464972	0.862075312	0.978543165	0.008515475
SVOP	0.917040439	0.860405851	0.977402892	0.007751642
MAOB	1.141351986	1.074120028	1.212792166	1.97E-05
AEBP1	1.174565359	1.109130938	1.243860156	3.77E-08
CA9	1.077428832	1.027014882	1.130317494	0.002286835
POSTN	1.047150236	1.01163345	1.083913958	0.008872309
TAC1	0.917980904	0.866084276	0.972987229	0.00394837
CCK	0.936474015	0.895981057	0.97879701	0.003611666
METTL7B	1.094579851	1.037712884	1.154563144	0.000900399
ETNPPL	0.917768981	0.87258621	0.96529133	0.000864076
LUZP2	0.926660604	0.877435079	0.97864776	0.006238724
LINC00689	0.913595394	0.864868214	0.965067892	0.001231708
CRYM	0.931898192	0.884828857	0.981471427	0.00764843
VEGFA	1.090522093	1.042393984	1.140872313	0.000167965

Supplementary Table 7. Applying the "Boruta" package to screen for important genes

gene	gene	gene	gene
ZDHHC22|A	AQP9|B	LOXL2|B	SERPINA1|B
FSD1|A	SPP1|B	ANXA1|B	SIGLEC7|B
KCNIP2|A	SERPINB1|B	S100A4|B	ITGB2|B
ENHO|A	SPOCD1|B	NNMT|B	PLAUR|B
GDAP1L1|A	S100A8|B	GPX8|B	CTSZ|B

RUNDC3A|A	NFKBIZ|B	PLTP|B	SLC11A1|B
TMEM145|A	BACE2|B	EFEMP1|B	DOK3|B
RIPPLY2|A	RDH10|B	TIMP1|B	PTPN7|B
CASKIN1|A	ANXA2|B	HSPA6|B	CTSC|B
SEZ6L|A	FAM20A|B	PDPN|B	FCGR2B|B
MMD2|A	CLEC5A|B	WWTR1|B	VSIG4|B
SCG3|A	CEBPD|B	BHLHE40|B	ICAM1|B
CKMT1B|A	OSMR|B	MMP14|B	LCP2|B
ATP1A3|A	TGFBI|B	ACTN1|B	MYOF|B
TSPAN7|A	COL1A2|B	TAGLN2|B	RAC2|B
BEX1|A	FOSL2|B	FNDC3B|B	FPR3|B
CKMT1A|A	TNFAIP2|B	LUM|B	C5AR1|B
OLIG1|A	RUNX1|B	CHI3L2|B	SLAMF8|B
CBLN1|A	NRP1|B	FZD7|B	NCF2|B
INA|A	IL4I1|B	FOSL1|B	MYO1G|B
SNAP91|A	LIF|B	CA12|B	HK3|B
ALDOC|A	PTX3|B	TNFRSF12A|B	C1R|B
NDRG2|A	PLAU|B	TUBA1C|B	TLR2|B
PHYHIPL|A	FCGBP|B	ADAM12|B	CD68|B
NEU4|A	COL3A1|B	MRC2|B	SLC16A3|B
RPRM|A	COL6A3|B	LGALS3|B	CTSL|B
CPLX1|A	CD93|B	MXRA5|B	C1S|B
LINC00634|A	CP|B	G0S2|B	C1QB|B
ATP2B2|A	FAM20C|B	COL6A2|B	SOCS3|B
ELFN2|A	OLFML2B|B	ACTA2|B	THBD|B
DLGAP1|A	MMP19|B	LTF|B	DPYD|B
SYP|A	HSPA7|B	ADAMTS1|B	CEBPB|B
PCDH15|A	PRSS23|B	RARRES1|B	TREM1|B
OLIG2|A	THBS1|B	TAGLN|B	FCGR2C|B
ATP6V1G2|A	PYGL|B	VASN|B	CSTA|B
UNC5A|A	FN1|B	SERPING1|B	CDCP1|B
NRXN1|A	CLIC1|B	TNC|B	S100A9|B
LINC00844|A	CAPG|B	IL6|B	NAMPT|B
GABRB3|A	FTL|B	UPP1|B	CLCF1|B
TNR|A	S100A11|B	COL5A2|B	C1RL|B
GRIA4|A	IRF1|B	ZFP36|B	C2|B
CPLX2|A	MAN1C1|B	RCAN1|B	CHI3L1|B
ABCC8|A	PLIN2|B	CNN2|B	MVP|B
CHGB|A	COL5A1|B	ITGA3|B	COL8A1|B
OMG|A	GPNMB|B	CAV1|B	GPR84|B
VIPR2|A	IBSP|B	STC1|B	GCNT1|B
PPP1R1A|A	RAB42|B	GBP1|B	TNFRSF1A|B
SVOP|A	MSN|B	VIM|B	STAB1|B
TAC1|A	A2M|B	EMILIN1|B	CFI|B

CCK|A
ETNPPL|A
LUZP2|A
LINC00689|A

BIRC3|B
TMBIM1|B
PROS1|B
SHC1|B
LOXL1|B
IGFBP3|B
ESM1|B
COL4A2|B
S100A10|B
SDC1|B
CA9|B
BCL3|B
CD14|B
CTSB|B
FCGR3A|B
CD163|B
ARPC1B|B
IFI30|B
MSR1|B

EMP1|B
EMP3|B
SERPINH1|B
C6orf141|B
MYL9|B
MMP11|B
GEM|B
LAMB1|B
PLP2|B
FSTL1|B
LGALS1|B
PODNL1|B
MYBPH|B
ANGPTL4|B
ADM|B
COL6A1|B
CD248|B
COL4A1|B

ITGA5|B
CSF1|B
TYMP|B
SAT1|B
LILRB3|B
SERPINE1|B
SOD2|B
CD44|B
TNFAIP3|B
CASP4|B
COL1A1|B
CCL2|B
SRPX2|B
STEAP3|B
ABCC3|B
LAIR1|B
SIGLEC9|B
FCGR2A|B
